# Multi-trait polygenic scores for COPD and COPD exacerbations implicate druggable proteins

**DOI:** 10.1172/jci.insight.199951

**Published:** 2026-02-19

**Authors:** Chengyue Zhang, Iain R. Konigsberg, Yixuan He, Jingzhou Zhang, Tinashe Chikowore, William B. Feldman, Xiaowei Hu, Yi Ding, Bogdan Pasaniuc, Diana Chang, Qingwen Chen, Jessica A. Lasky-Su, Julian Hecker, Martin D. Tobin, Jing Chen, Sean Kalra, Katherine A. Pratte, Hae Kyung Im, Emily S. Wan, Ani Manichaikul, Edwin K. Silverman, Russell P. Bowler, Leslie A. Lange, Victor E. Ortega, Alicia R. Martin, Michael H. Cho, Matthew R. Moll

**Affiliations:** 1 Channing Division of Network Medicine, Mass General Brigham, Boston, Massachusetts, USA.; 2 Department of Biomedical Informatics, University of Colorado Anschutz Medical Campus, Aurora, Colorado, USA.; 3 Department of Epidemiology School of Public Health, UTHealth Houston, Houston, Texas, USA.; 4 Pulmonary Center, Boston University School of Medicine, Boston, Massachusetts, USA.; 5 Harvard Medical School, Boston, Massachusetts, USA.; 6 Division of Pharmacoepidemiology and Pharmacoeconomics, Mass General Brigham, Boston, Massachusetts, USA.; 7 Department of Public Health Genomics, University of Virginia, Charlottesville, Virginia, USA.; 8 Department of Medicine, DanaFarber Cancer Institute, Boston, Massachusetts, USA.; 9 Center for Computational Biomedicine, Institute for Biomedical Informatics, Perelman School of Medicine, University of Pennsylvania, Philadelphia, Pennsylvania, USA.; 10 Genentech, South San Francisco, California, USA.; 11 Department of Population Health Sciences, University of Leicester, Leicester, United Kingdom.; 12 Division of Biostatistics and Bioinformatics, National Jewish Health, Denver, Colorado, USA.; 13 Department of Human Genetics, Department of Medicine, University of Chicago, Chicago, Illinois, USA.; 14 Section on Pulmonary, Critical Care, Allergy, and Sleep Medicine, VA Boston Healthcare System, West Roxbury, Massachusetts, USA.; 15 Public Health Genomics Program, University of Virginia, Charlottesville, Virginia, USA.; 16 Department of Genetic Medicine, Cleveland Clinic Lerner College of Medicine, Cleveland, Ohio, USA.; 17 Division of Pulmonary and Critical Care Medicine, Mayo Clinic, Phoenix, Arizona, USA.; 18 Analytic and Translational Genetics Unit, Massachusetts General Hospital, Boston, Massachusetts, USA.; 19 Program in Medical & Population Genetics, Broad Institute, Cambridge, Massachusetts, USA.

**Keywords:** Genetics, Pulmonology, COPD, Genetic risk factors, Proteomics

## Abstract

**BACKGROUND:**

We constructed multi-trait polygenic risk scores (PRSs) predicting chronic obstructive pulmonary disease (COPD) and exacerbations, validated their performance in diverse cohorts, and identified PRS-related proteins for potential therapeutic targeting.

**METHODS:**

PRSmix^^+^^, a multi-trait PRS framework, is used to train a composite PRS (PRS__multi__) in COPDGene non-Hispanic White participants (*n* = 6,647). Associations of PRS__multi__ with COPD status (GOLD 2–4 vs. GOLD 0 or ICD) and exacerbation frequency were tested in COPDGene African American (*n* = 2,466), ECLIPSE (*n* = 1,858), Mass General Brigham Biobank (*n* = 15,152), and All of Us (*n* = 118,566). Protein prediction models were applied to GWAS summary statistics from traits contributing to PRS__multi__ and were validated with proteomic data in COPDGene (*n* = 5,173) and UK Biobank (*n* = 5,012).

**RESULTS:**

PRSmix^^+^^ selected 7 traits for PRS__multi__. In multivariable models, PRS__multi__ was associated with COPD status (meta-analysis random effects [RE] OR 1.58 [95% CI: 1.28–1.94]) and exacerbation frequency (meta-analysis RE **β** 0.21 [95% CI: 0.11–0.31]), with higher effect sizes observed in smoking-enriched cohorts. PRS__multi__ outperformed traditional single-trait PRS in all tested cohorts. Using protein prediction models, we identified 73 proteins associated with the PRSs that were also validated with measured protein levels in COPDGene and UK Biobank. Of these proteins, 25 were linked to approved or investigational drugs. Notable targets include RAGE/sRAGE, IL1RL1, and SCARF2, all implicated in COPD pathogenesis and exacerbations.

**CONCLUSIONS:**

Multi-trait PRS improves prediction of COPD and exacerbation risk. Integration with proteomic data identifies druggable protein targets, offering a promising avenue for precision medicine in COPD management.

**TRIAL REGISTRATION:**

COPDGene: ClinicalTrials.gov NCT00608764; ECLIPSE: ClinicalTrials.gov NCT00292552.

## Abstract 2

A multi-trait genetic risk score improves predictions of chronic obstructive pulmonary disease (COPD) and exacerbations and reveals protein targets for potential therapeutic targeting.

## Introduction

Chronic obstructive pulmonary disease (COPD), a leading global cause of morbidity and mortality, is marked by persistent airflow limitation and an enhanced inflammatory lung response to harmful inhaled particles or gases. COPD is highly heterogeneous in presentation, progression, therapy response, and exacerbations, the latter of which are major drivers of morbidity (1). Comorbid conditions, such as cardiovascular and metabolic diseases, are important predictors of COPD-related mortality. However, susceptibility to COPD and its comorbidities is determined by complex gene-by-environment interactions (2). Notably, only a minority of smokers develop COPD, with genetics accounting for approximately 30%–40% of the variability in susceptibility (3).

Genome-wide association studies (GWAS) have identified numerous genetic variants linked to lung function and COPD (4). Summing GWAS variants together, polygenic risk scores (PRSs) for 2 measures of lung function (forced expiratory volume at 1 second [FEV__1__] and the ratio of FEV__1__ to forced vital capacity [FEV__1__/FVC]) were combined into a COPD PRS that was highly predictive of COPD in multiple cohorts, outperforming single PRSs in predicting COPD and COPD-related phenotypes (5). Recent findings highlight that BMI genetics can predict mortality in patients with COPD, underscoring the importance of multiple-trait (multi-trait) genetic analyses in understanding COPD risk and outcomes (6).

Multi-trait genetic analyses, which identify shared mechanisms among traits, have gained traction with the rise of a multitude of PRSs for various traits. He et al. developed a multi-trait PRS for COPD that performed exceptionally well in biobanks (7). Furthermore, this multi-trait PRS and the prior lung function–based COPD PRS were associated with exacerbations. However, in the latter, the COPD PRS effects were attenuated when accounting for baseline lung function, consistent with the known clinical association between COPD severity and exacerbations. It remains unclear whether combining PRSs for spirometry and broader phenotypes can better predict COPD and exacerbations across diverse cohorts.

While shared genetics can inform phenotype prediction and reveal insights into disease mechanisms, proteins offer several advantages. First, proteins are influenced by both genetics and environmental factors, such as infections — the leading cause of exacerbations (8). Second, proteins can serve as direct therapeutic targets for small molecules (9). Third, recent advances in statistical tools can use genetics to predict protein expression levels (10), which are more likely causally related to a trait than non–genetically regulated proteins (11). We hypothesized that integrating PRSs for spirometry and comorbid traits could enhance the prediction of COPD and exacerbations in both research and biobank cohorts. Furthermore, we sought to identify and validate genetically predicted protein levels linked to shared genetic architectures, using measured protein levels to confirm key findings. Our main goal was to leverage polygenic risk to identify protein drug targets associated with exacerbations through generation of an improved multi-trait PRS and identification of proteins associated with exacerbations and genetic scores.

## Results

**Figure 1:**
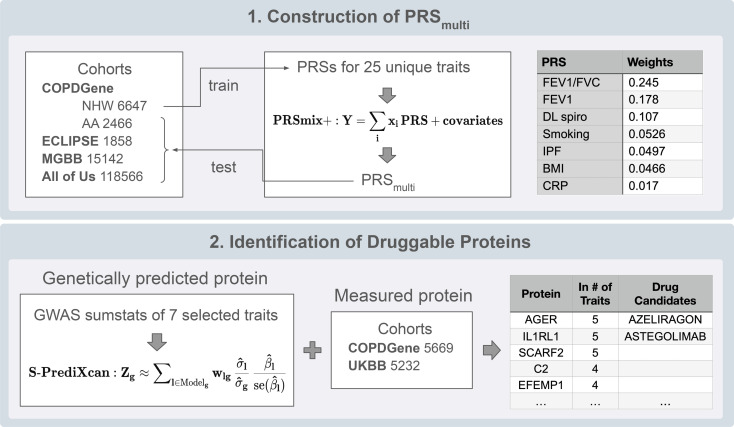
Overview of study design. Schematic representation of the study workflow. Plots included in this figure are intended for illustrative purposes only.

An overview of the study design is shown in Figure 1 and described in Methods.

### Characteristics of study participants.

A total of 144,679 individuals were included in genetic analyses (Table 1), drawn from both COPD-enriched cohorts (Genetic Epidemiology of COPD [COPDGene] and Evaluation of COPD Longitudinally to Identify Predictive Surrogate Endpoints [ECLIPSE]) and population-scale biobanks (Mass General Brigham Biobank [MGBB] and All of Us). As expected, smoking prevalence varied across cohorts.

Cohort characteristics for COPDGene and UK Biobank (UKBB) proteomic analyses are shown in Supplemental Table 1; supplemental material available online with this article; https://doi.org/10.1172/jci.insight.199951DS1 Participants had similar age, sex distribution, and spirometric measures. In COPDGene, individuals who were never smokers were excluded.

### Development of multi-trait PRSs for COPD and related traits.

We used 25 individual PRSs (Supplemental Table 2), chosen based on clinician input and literature review, to develop multi-trait PRSs for COPD and related traits. Four new PRSs were developed for this analysis (emphysema, peak expiratory flow, eosinophils, and smoking) which are available upon request. Clustering analysis revealed that PRSs for spirometry were highly correlated and that the emphysema and type 2 diabetes scores were correlated, as were asthma/allergic rhinitis and venous thromboembolism/cor pulmonale (Supplemental Figure 1).

We constructed multi-trait PRS models for COPD status (Global Initiative for Chronic Obstructive Lung Disease [GOLD] stages 2–4 vs. GOLD 0) and exacerbations, as well as computed tomography (CT) measures of quantitative emphysema (adjusted lung density) and airway wall thickness (square root of the wall area of a hypothetical airway with an internal perimeter of 10 millimeters, Pi10) using PRSmix^^+^^. Mixing weights for each model are displayed in Supplemental Table 3. Final weighted models included between 2 and 7 individual PRSs. Notably, PRSs for FEV__1__/FVC (PRS__ratio__) and a deep learning spirometry (PRS__DLspiro__) trait contribute to all 4 traits. PRSs for FEV__1__ (PRS__FEV1__), BMI (PRS__BMI__), and CRP (PRS__CRP__) contribute to both Pi10 and COPD, and all 7 PRSs, including smoking (cigarettes per day) (PRS__smoke__) and idiopathic pulmonary fibrosis (PRS__IPF__), contribute to COPD. Thus, while PRS__ratio__ is the largest contributor to COPD genetic risk, 6 additional PRSs also contribute to COPD risk.

All multi-trait PRSs showed strong associations with their respective training outcomes in multivariable models (Supplemental Table 4). The PRS__mix+__ models for emphysema (adjusted lung density) and airway thickness (Pi10) were associated with COPD and exacerbations, and inversely associated with their respective outcomes (e.g., higher PRS__mix+__ [Pi10] is associated with lower quantitative emphysema). As our a priori decision was to select the multi-trait PRS that represents the highest number of comorbid traits, the PRS for COPD derived from 7 separate PRSs was carried forward for further testing and will hereafter be referred to as the PRS__multi__. The PRS__multi__ explained 20.7% of the variance on the liability scale for COPD (12).

### Testing the multi-trait PRS for COPD.

We tested the predictive utility of PRS__multi__ for both COPD and COPD exacerbations compared with individual component PRSs. For COPD, PRS__multi__ consistently demonstrated larger absolute effect sizes in all cohorts except All of Us, where PRS__BMI__ showed the strongest effect (Supplemental Table 5). For COPD exacerbations, PRS__multi__ outperformed other scores in COPDGene non-Hispanic White (NHW) and ECLIPSE, whereas PRS__BMI__ had higher effects in the remaining cohorts (Supplemental Table 6). Notably, PRS__multi__ associations with COPD status remained significant after adjusting for baseline FEV__1__ in COPDGene, but associations with exacerbations were attenuated in FEV__1__-adjusted models.

**Figure 2:**
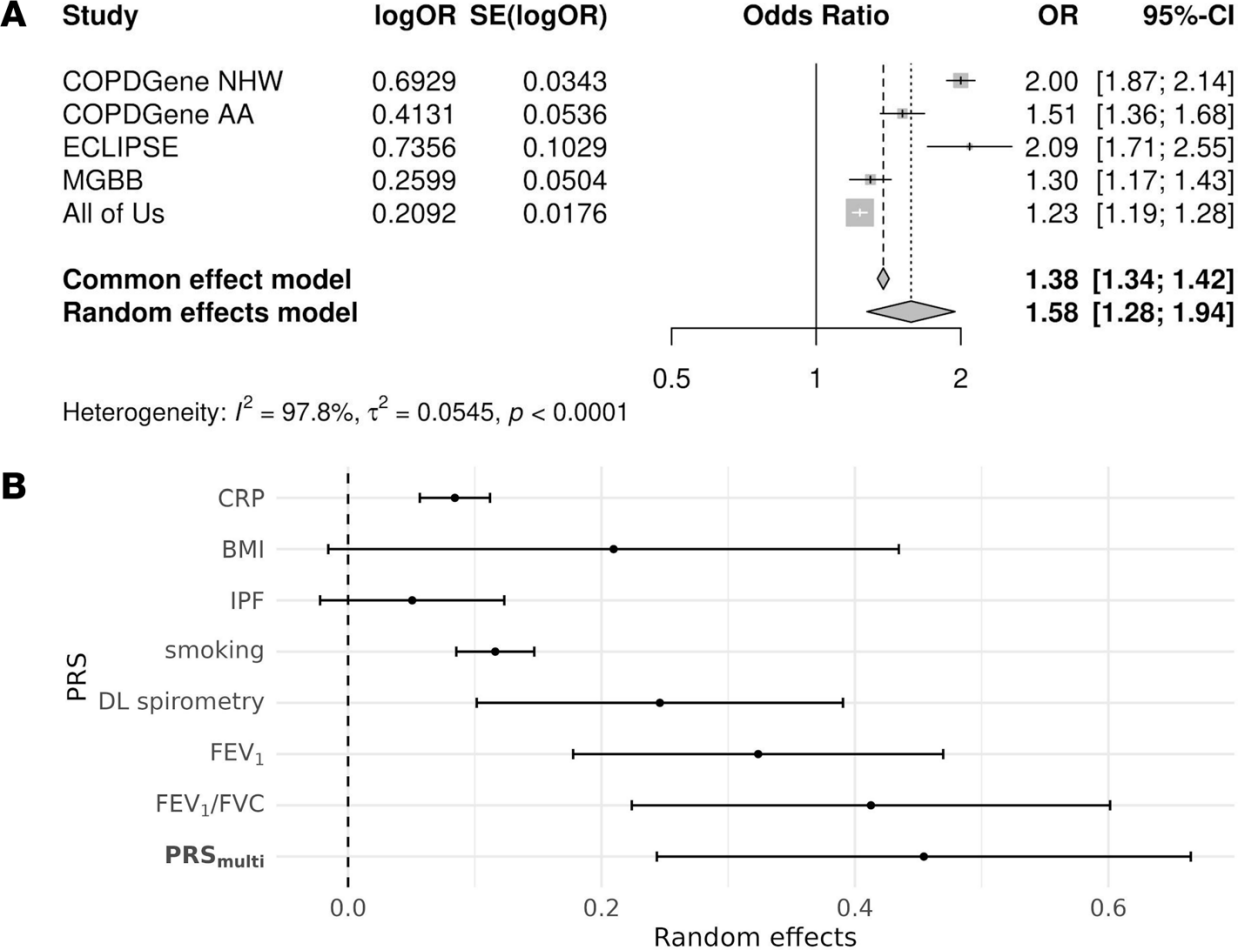
PRS_multi_ is associated with COPD. (**A**) Forest plot from a meta-analysis of the association between PRS_multi_ and COPD status across multiple cohorts. The figure presents ORs with 95% CIs for each cohort, demonstrating the overall effect size and heterogeneity (*I*^2^). (**B**) Forest plot comparing the effects of individual PRSs and PRS_multi_ on COPD risk. PRS_multi_ outperforms single-trait PRSs, particularly in smoking-enriched cohorts. CRP, C-reactive protein; BMI, body mass index; IPF, idiopathic pulmonary fibrosis; DL, deep learning; PRS, polygenic risk score. Cohort abbreviations are in the legend for Table 1.

In a random effects meta-analysis, PRS__multi__ was associated with increased odds of COPD (OR: 1.58 per SD [95% CI: 1.28–1.94]) (Figure 2A). We observed significant between-study heterogeneity (*I*^^2^^ = 0.98, *P* < 0.01), with stronger effects in smoking-enriched research cohorts. Among the component PRSs, PRS__IPF__ and PRS__BMI__ were the only scores not significantly associated with COPD, and PRS__multi__ had a larger effect on COPD status than any individual score (Figure 2B).

**Figure 3:**
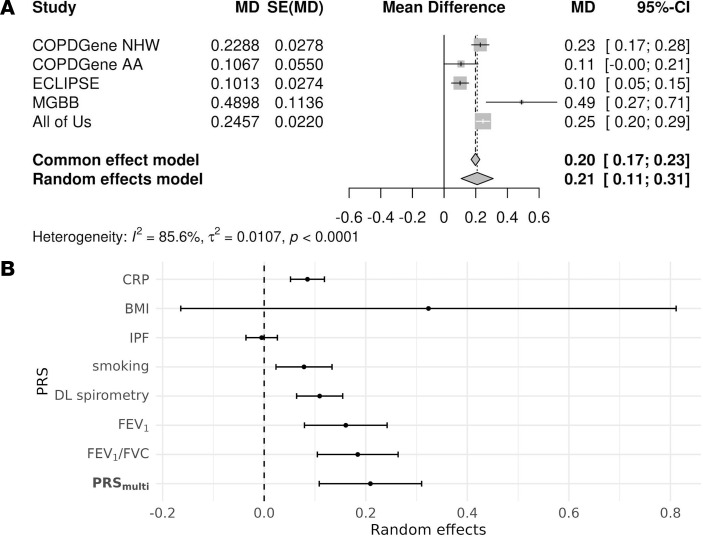
PRS is associated with COPD exacerbations. (**A**) Meta-analysis forest plot showing the association between PRS_multi_ and COPD exacerbations, with estimates from individual cohorts and the overall effect. The PRS_multi_ is significantly associated with increased exacerbation risk. (**B**) Comparison of individual PRSs and PRS_multi_ for exacerbation prediction. Effect sizes and confidence intervals are presented, highlighting PRS_multi_ as the most predictive score. Abbreviations are in the legends of Figure 2 and Table 1.

We next performed a similar analysis for COPD exacerbations but with multivariable negative binomial modeling of count data. In meta-analysis, PRS__multi__ was associated with an increase of 0.21 exacerbations per year per SD (95% CI: 0.11–0.31) (Figure 3A). However, this association was attenuated in sensitivity analyses adjusting for baseline FEV__1__. All component PRSs were significantly associated with exacerbations except for the PRS__IPF__ and PRS__BMI__ (Figure 3B). We note that PRS__BMI__ demonstrated the largest effects on exacerbations compared with other PRSs in some cohorts (Supplemental Table 6), but it was not significantly associated with exacerbations in meta-analysis (Supplemental Figure 2) due to a flipped direction of effect in COPDGene African American (AA) participants. Again, between-study heterogeneity was significant (*I*^^2^^ = 0.86, *P* < 0.0001), although less pronounced than for COPD status.

We also observed that heterogeneity in COPD status associations was driven largely by differences between research and biobank cohorts, reflecting the use of spirometry- versus International Classification of Diseases–based (ICD-based) case definitions, respectively (Supplemental Figure 3A). In contrast, heterogeneity in exacerbation outcomes was lower and appeared to be influenced by outlier results from MGBB participants (Supplemental Figure 3B). In a leave-one-out sensitivity analysis excluding COPDGene NHW participants, we observed similar results for COPD and COPD exacerbations (Supplemental Figure 4).

In AUC analyses among COPDGene NHW participants, PRS__multi__ outperformed PRS__ratio__ in predicting both COPD (AUC 0.67 vs. 0.64, *P* = 1.05 × 10^^–8^^) and frequent exacerbations (≥2 per year) (AUC 0.569 vs. 0.562, *P* = 0.000001). Both PRSs improve prediction when added to clinical covariates (Table 2 and Supplemental Figure 5). For COPD, the AUC increased from 0.756 (clinical only) to 0.8 with PRS__multi__ and 0.79 with the PRS__ratio__. For exacerbation prediction, the AUC increased from 0.599 to 0.612 and 0.609, respectively. The PRS__multi__ also demonstrated higher predictive capacity for COPD and frequent exacerbations compared with any of the other tested single-trait PRSs or the previously published PRS__FEV1+FEV1/FVC__ from Moll et al. (5) (Supplemental Table 9).

Examining other COPD-related outcomes, we found that a higher PRS__BMI__ was the PRS most associated with all-cause mortality, while the PRS__DLspiro__ was most highly associated with respiratory mortality (Supplemental Table 10). However, the PRS__multi__ had the largest effects on severe exacerbations and antibiotic or steroid use compared with other tested PRSs (Supplemental Table 10).

### Genetically predicted proteins based on shared genetic risks among traits.

Following the identification of 7 traits contributing to COPD genetic risk, we investigated shared genetic mechanisms by identifying proteins whose levels are predicted to be altered based on the genetic architecture of these traits. Using GWAS summary statistics for each trait, we applied S-PrediXcan (10) with Atherosclerosis Risk in Communities (ARIC) PredictDB protein expression models (13) to infer genetically regulated protein levels (full results available upon request). We then tested whether measured plasma protein levels were associated with COPD exacerbations using multivariable negative binomial regression models in COPDGene and UKBB. Models were adjusted for COPD case-control status and other confounders (see Methods). We examined only proteins meeting FDR-adjusted significance in S-PrediXcan, COPDGene SomaScan (measured protein levels), and UKBB Olink (measured protein levels) data (Supplemental Table 7). We further restricted the protein list to those with concordant directions of effects in COPDGene and UKBB, resulting in a final set of 73 genetically predicted proteins associated with COPD exacerbations (Table 3). The number of proteins at each filtering step for all 7 traits are detailed in Supplemental Table 11.

We ranked these proteins by the number of traits for which all criteria were met. The receptor for advanced glycation end products (RAGE) and its soluble form (sRAGE), IL-1 receptor–like 1 (IL1RL1), and scavenger receptor class F member 2 (SCARF2) were represented in 5 out of 7 tested traits. SERPINA1 was represented in 2 out of 7 tested traits. We performed gene name lookups in the OpenTargets Platform (14) to identify potential drug repurposing candidates. We identified 25 proteins with existing clinical trials for 46 drugs (Table 3 and Supplemental Table 8). Notably, we found drugs targeting RAGE/sRAGE and IL1RL1 that are being tested in clinical trials, with the latter being tested in COPD. Finally, among the 73 proteins, we identified 8 (agrin [AGRN], CD300C, CFB, GM2 ganglioside activator [GM2A], IL1RL1, INHBB, leukocyte immunoglobulin-like receptor A5 [LILRA5], and TIMP4) that demonstrated consistent associations with COPD status, frequent exacerbations, and the highest versus lowest quintile of PRS__multi__ in COPDGene NHW participants (Supplemental Figure 6). Compared with IL1RL1, RAGE/sRAGE, and SCARF2 protein levels, PRS__multi__ demonstrated higher predictive capacity for frequent exacerbations (Supplemental Figure 8; AUC 0.77, all *P* < 0.05).

## Discussion

In this study, we leveraged genetic data from over 140,000 individuals across smoking-enriched research cohorts and large-scale biobanks to develop a composite multi-trait PRS (PRS__multi__) for COPD and exacerbations. PRS__multi__ outperformed single-trait PRSs, the previously published PRS__FEV1+FEV1/FVC__ from Moll et al. (5), and protein levels of RAGE/sRAGE, IL1RL1, and SCARF2 for predicting both COPD risk and exacerbation frequency, demonstrating the advantage of leveraging multiple trait genetics to understand drivers of disease activity. Using elastic net modeling, we developed a multi-trait PRS and identified 7 component traits that share a genetic basis with COPD, including spirometry measures such as FEV__1__/FVC and FEV__1__, a deep learning–derived spirometry phenotype, cigarettes per day, pulmonary fibrosis, C-reactive protein (CRP), and BMI. Using the genetic associations for these traits, we applied protein prediction models and identified 73 genetically predicted proteins whose measured levels were associated with exacerbations in both a smoking-enriched and a biobank cohort, independent of COPD status. These findings provide critical insights into the shared genetic architecture of COPD and related traits and nominate a set of proteins as potential biomarkers and therapeutic targets.

Although COPD comorbidities are known predictors of exacerbations and mortality, their genetic contributions to COPD risk have been underexplored. We identified 7 traits contributing to the genetic risk for COPD, emphasizing its complex genetic architecture. Among these, 3 are spirometry-based, including a deep learning–derived spirometry phenotype, indicating that traditional measures like FEV__1__ and FEV__1__/FVC alone may not fully capture genetic risk or phenotypic variability. Our decision to use the COPD PRS__mix+__ model was based on an a priori principle of taking the model with the greatest number of traits in order to capture the greatest subphenotypic genetic variation. Nonetheless, the other PRS__mix+__ models demonstrated interesting findings in research cohorts. For example, the inverse association of airway- and emphysema-based PRSs with the other outcome (e.g., higher PRS__mix+__ [Pi10] is associated with lower quantitative emphysema) is consistent with mounting evidence that COPD exists along emphysema- and airway-predominant axes (15, 16). Future studies can examine how these PRSs for imaging traits can be used to dissect disease heterogeneity.

Notably, the PRSs of several traits known to be associated with exacerbations were not significant in elastic net modeling, including eosinophils and asthma, which have previously demonstrated genetic overlap with COPD (17, 18). This observation could reflect inclusion of relevant overlapping loci into other risk scores (e.g., asthma risk loci included in COPD or lung function), phenotypic specificity (e.g., data on eosinophilic COPD was not uniformly available), or other factors. These findings emphasize the need to understand the shared genetic architecture of COPD and inflammation-related traits. The emphysema PRS was developed from the largest GWAS of quantitative CT emphysema (19) but was not chosen in any of our PRS__mix+__ models. The reason for this result is unclear, but when tuned to quantitative emphysema, PRS__ratio__ and PRS__DLspiro__ were selected, suggesting that the effects of emphysema might be largely captured by the genetics of low lung function.

The PRS__BMI__ was most associated with all-cause mortality, which likely reflects higher cardiovascular risks in individuals with a higher BMI; as a corollary, it was the PRS__DLspiro__ that was actually most highly associated with respiratory mortality, suggesting that variants associated with low lung function beyond FEV__1__ and FEV__1__/FVC are important predictors of COPD mortality. The PRS__multi__ was also associated with severe exacerbations, suggesting that it could be used to enrich a population for severe exacerbators, although further validation is needed.

Although the PRS__multi__ outperformed single-trait PRSs and the previously published PRS__FEV1+FEV1/FVC__ from a statistical perspective, we acknowledge that this improved performance likely translates to incremental clinical gains; similar observations have been made in PRSs for cardiovascular diseases, yet the utility for both coronary disease and COPD seem to be for earlier age ranges (20, 21) and for case finding (22). In addition to this clinical context, the purpose of the current study was not to develop a better genetic prediction tool, but rather to leverage the shared genetic architecture across multiple traits to gain biological insights into risk of COPD and exacerbations. Indeed, there is broad interest in both multi-trait genetics (23, 24) and genetic-protein prediction (25, 26), and here we apply these principles and domain knowledge in the case of COPD.

To investigate shared genetic mechanisms between these 7 traits, we used genetic protein prediction models derived from protein quantitative trait loci from the ARIC study (13). Using genetics to predict other omics has been shown to enhance prediction of complex traits; for example, polygenic transcriptome risk scores for spirometry using PrediXcan demonstrated greater portability across self-identified race and ethnicity groups compared with a standard PRS (27).

Instead of focusing on a single trait, we applied a genetic protein prediction approach across 7 traits and validated our findings using measured protein levels in 2 cohorts. As a result, we identified 73 genetically predicted proteins associated with exacerbations in both cohorts after adjusting for COPD case-control status. To find the most relevant potential biomarkers for exacerbations, we analyzed proteins that were elevated in COPD cases, frequent exacerbators (≥2 exacerbations per year), and individuals in the highest quintile of PRS__multi__; we identified 8 key proteins: AGRN, CD300C, CFB, GM2A, IL1RL1, INHBB, LILRA5, and TIMP4. These findings suggest that PRS__multi__ and these proteins could be measured concurrently to predict COPD exacerbation risk. Developing an integrated prediction model and subsequent prospective validation are needed.

As drugs with genetically backed targets lead to approval rates twice that of non–genetically backed compounds, and drug repurposing agents achieve markedly higher approval rates (30% compared with 10% for de novo drugs), we used the genetically predicted proteins associated with exacerbations to identify drug repurposing candidates (28–30). Our analysis revealed 25 proteins with trials involving 46 drugs.

Based on 5 out of 7 traits, 3 proteins were genetically predicted to affect COPD and exacerbation risk: IL1RL1, RAGE/sRAGE, and SCARF2. IL1RL1 is targeted by astegolimab, which is already in phase III trials for COPD. This finding offers a proof-of-concept that our method can highlight potential drug repurposing candidates for COPD exacerbations. *AGER* (RAGE/sRAGE), a highly replicable GWAS locus and well-established COPD biomarker (31), is expressed in alveolar epithelial cells and appears to have a broad role in regulating immunity and inflammation. The proteomic assays used quantify sRAGE rather than membrane-bound RAGE. RAGE in plasma arises from 2 sources: (a) the secretory isoform generated by alternative splicing, and (b) ectodomain shedding of RAGE by protease activity, which are not distinguished by the analyzed assays. Furthermore, as full-length RAGE can be cleaved by multiple proteases (e.g., MMP9, ADAM17), changes in protease pathways are not captured in the current study. RAGE/sRAGE has been implicated in several diseases, including glioblastoma multiforme (ClinicalTrials.gov NCT05986851), triple-negative breast cancer (32), and Alzheimer disease (33). Azeliragon is an antagonist of RAGE/sRAGE and was well tolerated in a phase III trial of Alzheimer disease, but the trial (ClinicalTrials.gov NCT02080364) was terminated due to lack of efficacy. The complicated issue in using azeliragon in COPD is that the rs2070600 C→T variant, associated with higher COPD risk, is associated with lower sRAGE levels, and lower sRAGE levels are associated with more emphysema (34). The effects of this variant on exacerbations are less clear, and further investigation needs to be done on which patients might benefit from repurposing azeliragon for COPD exacerbations.

SCARF2 polymorphisms have been implicated in COPD risk based on Mendelian randomization (35), and we now report that this genetically predicted protein is associated with exacerbations at the measured protein level in 2 cohorts. We did not identify any drug repurposing candidates in OpenTargets based on SCARF2, making this protein a potential novel therapeutic target for exacerbations.

Strengths of this study are that it combines many participants from both research and biobank cohorts with carefully defined COPD and COPD exacerbation criteria. The use of PRSmix^^+^^ facilitated the identification of key traits contributing to genetic risk and the discovery of genetically predicted protein biomarkers. While individual S-PrediXcan studies have been published as stand-alone analyses (10), our study extends this framework by performing 7 proteome-wide association analyses and validating key signals with measured protein levels in 2 independent cohorts, which identified several promising biomarkers and therapeutic targets.

There are several limitations. PRSmix^^+^^ studies typically aim to improve prediction, and while including more PRSs as inputs may enhance predictive power, we are approaching the upper bound of heritability for COPD, limiting further gains. The list of traits analyzed is not exhaustive, and translating findings from European to non-European cohorts remains a challenge. Cohort design limitations, such as recruiting only NHW and AA individuals in COPDGene, may also influence results. However, we found similar results in All of Us when including all groups, emphasizing the value of diverse representative cohorts. Definitions of COPD and exacerbations differ between research cohorts and biobanks, and parameters that would help reduce heterogeneity and improve uniformity of these definitions, such as exacerbation type (36) and lung function (in biobanks), was not readily available. However, the fact that the PRS__multi__ can predict COPD and exacerbations across both research and biobank cohorts suggests that our genetic and proteomic signals generalize across case definitions rather than being confined to a single operationalization of COPD; that is, our findings support generalizability and transferability. We previously tested the PRS__FEV1+FEV1/FVC__ in biobanks using validated machine learning phenotypes for COPD, and observed smaller effect sizes than we found in the current study (17). Future studies with additional phenotyping may allow increased effect size or specificity of our results.

Importantly, PRS__multi__ associations with exacerbations were attenuated when adjusting for baseline FEV__1__ in research cohorts, suggesting the effects are primarily driven by disease severity; by contrast, measured protein associations were adjusted for COPD status, suggesting that these genetically predicted protein targets are also important for disease activity and exacerbation risk. Finally, the integration of genetic and protein biomarkers, coupled with prospective validation and clinical implementation studies, is crucial to establish the practical utility and clinical relevance of these findings.

In conclusion, multi-trait PRSs for COPD and exacerbations identify genetic contributions to disease heterogeneity and druggable protein targets. These findings suggest that genetic risk prediction can be linked to specific therapeutic strategies for COPD precision medicine.

## Methods

Additional cohort details, genotyping information, and proteomic details are in the Supplemental Methods.

### Sex as a biological variable

Our study included both male and female participants and analyses were conducted jointly with sex included as an adjustment variable.

### Study cohorts

#### COPDGene.

The COPDGene study (ClinicalTrials.gov NCT00608764) is a smoking-enriched cohort of self-identified NHW and AA participants aged 45–80 years with 10 or more pack-years of smoking history (37). COPDGene had enrollment and 5- and 10-year visits. Proteomic data were collected at visit 2 (5-year follow-up).

#### ECLIPSE.

The ECLIPSE study (ClinicalTrials.gov NCT00292552) was a multicenter cohort study designed to explore biomarkers and clinical phenotypes of COPD (38). Participants include individuals with moderate to severe COPD (GOLD stages 2–4), aged 40–75 years, recruited across 12 countries.

#### MGBB.

The MGBB is a large-scale biorepository linked to electronic health records (EHRs) of over 117,000 participants from the Mass General Brigham healthcare system since 2009. Participants aged 18 years or older were genotyped using the Illumina Global Screening Array.

#### All of Us.

The All of Us Research Program is a population-scale biobank designed to advance precision medicine by collecting genetic, environmental, and health data from over 1 million participants in the United States. Whole-genome sequencing was conducted using the Illumina NovaSeq platform, as previously described (39). We utilized the allele call/allele frequency (ACAF) dataset for analyses.

### Statistics

#### Overview of study design.

We integrated genetic data from COPDGene, ECLIPSE, MGBB, UKBB, and All of Us to develop and validate a PRS__multi__ for both COPD and exacerbations. Following genotype imputation and PRS construction, we applied PRSmix^^+^^ to combine trait-specific PRSs relevant to COPD susceptibility and exacerbation risk (40). We assessed associations with COPD case-control status and exacerbations. In COPDGene, NHW and AA participants were analyzed separately due to differences in demographic and disease characteristics, partly shaped by recruitment strategies. In other cohorts, racial/ethnic groups were analyzed together, adjusting for self-identified race and principal components (PCs) of genetic ancestry.

To identify underlying biological mechanisms, we integrated genetically predicted protein levels (via S-PrediXcan) with measured plasma proteomic data from COPDGene and UKBB to identify proteins linked to COPD and exacerbations (10). These candidate proteins were then cross-referenced with drug databases to highlight potential targets for therapeutic repurposing.

### Outcomes

### COPD

#### COPD in research cohorts.

COPD was defined using the GOLD classification system. Two groups were identified: GOLD 2–4, representing those with moderate to very severe airflow limitation (FEV__1__/FVC < 0.7 and FEV__1__ < 80% predicted), and GOLD 0, referring to those with normal spirometry (FEV__1__/FVC ≥ 0.7 and FEV__1__ ≥ 80% predicted).

#### COPD in biobanks.

For genetic analyses, we included individuals aged 40 years or older with available smoking data. In All of Us and MGBB, we excluded people with ICD codes for interstitial lung disease (J84) and heart failure (I50). The index date was defined as the date of the first COPD exacerbation or the first recorded observation for controls. COPD was defined by 1 or more inpatient code or 3 or more outpatient diagnostic codes within the past 3 years (41) to ensure identification of individuals with active disease. We identified COPD patients using ICD-9-CM codes 491.xx, 492.xx, and 496 and ICD-10-CM codes J41.x, J42, J43.x, and J44.x (42).

In UKBB, COPD case-control status was defined using spirometry to align with the definition in research cohorts (see below). A CONSORT diagram of participant inclusion and exclusion is shown in Supplemental Figure 7.

### COPD exacerbations

#### Exacerbations in research cohorts.

In COPDGene, annualized exacerbation rates were calculated from the first study visit using data from the Longitudinal Follow-up Program, which was designed to continuously collect data on clinical outcomes, including exacerbations, comorbidities, and mortality, through surveys and electronic health records (43). As COPDGene was enriched for individuals who smoke, and individuals without COPD in this cohort experience respiratory events and increased mortality, we included these individuals in analyses (44). In ECLIPSE, exacerbation data were collected at each study visit using standardized questionnaires that recorded the number of exacerbations and use of steroids or antibiotics since the prior visit. As a result of study inclusion and exclusion criteria, nearly all participants with genetic data had had moderate-to-severe COPD.

In COPDGene, we additionally assessed quantitative CT measures of emphysema (volume-noise-bias-adjusted lung density) (45) and airway pathology (Pi10).

#### Exacerbations in biobanks.

COPD exacerbation numbers were tracked for 1 year following the index date. Events were identified using ICD-9-CM codes 491.21 and 493.22 and ICD-10-CM codes J44.0 and J44.1. Multiple events within a 15-day period were considered a single exacerbation. For proteomic analysis, only exacerbations occurring after proteomic sampling were included in the analysis.

### Predictors

#### Single-trait PRSs.

PRSs were computed using GWAS summary statistics or obtained from the Polygenic Score Catalog (46, 47). Selection of PRSs was based on biological relevance according to clinician input and literature review, available summary statistics, and the exclusion of testing cohorts from the original GWAS to avoid overfitting.

Details of the GWAS used for PRS construction are in Supplemental Table 2 and the Supplemental Methods. In cases where a Polygenic Score Catalog accession number is not listed, we developed our own PRS either in a prior study or for the current study. For the current study, we developed PRSs for emphysema, deep learning spirometry, peak expiratory flow, smoking, and eosinophils. Variant weights are available upon request.

#### Development of a multi-trait PRS.

Prior to constructing a multi-trait PRS, we oriented directions of effects of individual PRSs such that a higher PRS is associated with higher COPD risk (e.g., higher PRS__FEV1__ is associated with higher COPD risk) since it is not clear what the expected effects are for all traits. Several methods exist for calculating multi-trait PRSs. A detailed description of PRSmix and PRSmix^^+^^ (40) is in the Supplemental Methods.

We trained PRSmix^^+^^ using COPDGene NHW participants, as most component PRSs were originally developed in populations with high genetic similarity to European reference panels. Models were trained to predict COPD, COPD exacerbations, Pi10, and emphysema, adjusting for age, sex, smoking pack-years, BMI, 5 genetic PCs, and CT scanner model (as appropriate). We selected the model that incorporated the largest number of PRSs and used the resulting weights to construct the final multi-trait PRS (PRS__multi__). All PRSs were centered and scaled prior to analysis, ensuring a mean of 0 and a standard deviation (SD) of 1.

### Testing of the multi-trait PRS

We tested the association of PRS__multi__ with COPD and exacerbations using multivariable logistic regression models and negative binomial models, respectively. All models were adjusted for age, sex, smoking pack-years, BMI, and 5 genetic PCs. Exacerbation models included a log-offset for follow-up time. For comparison, we performed these same regression analyses using each individual component PRS included in PRS__multi__. To account for multiple comparisons, statistical significance was defined as a Benjamini-Hochberg–adjusted (BH-adjusted) *P* value of less than 0.05. As a sensitivity analysis, we further adjusted models for baseline FEV__1__ in research cohorts. We performed area under the receiver operating characteristic curve (AUC) analyses, which are detailed in the Supplemental Methods.

We examined the multivariable associations of single-trait PRSs, the previously published PRS__FEV1+FEV1/FVC__, and the PRS__multi__ with all-cause and respiratory mortality (Cox models), severe exacerbations requiring ER visit or hospitalization and antibiotic or steroid use (negative binomial models).

### Meta-analysis

Following multivariable regression analyses, we performed random effects meta-analyses using the meta R package for PRS__multi__ and component PRSs (48). Heterogeneity was assessed using the *I*^^2^^ statistic, and funnel plots were generated to visualize intercohort variability and assess selection bias.

### Protein prediction

We applied the S-PrediXcan framework, which leverages GWAS summary statistics to infer associations between predicted protein expression and complex traits or diseases, to evaluate genetically predicted protein levels. Summary statistics from the PRSs contributing to PRS__multi__ (10) served as inputs. S-PrediXcan combines protein-wide association models with GWAS summary statistics, enabling the identification of protein-trait associations without requiring individual-level data. Prior to analysis, we harmonized GWAS variants to the GTEx v8 reference panel (49) and imputed summary statistics to address ambiguous or missing variants. We then applied multi-ancestry protein prediction models from the ARIC study (13) to estimate genetically regulated proteins with significantly altered levels.

For significant proteins (BH FDR–adjusted *P* < 0.1), we assessed whether directly measured protein levels were associated with COPD status and exacerbations in both COPDGene and UKBB. In COPDGene, models were adjusted for age, sex, race, pack-years of smoking, COPD case-control status, and log offset of time. In UKBB, analyses used propensity score matching on these variables, except the log offset of time since exacerbations were followed for 1 year, to account for case-control imbalance.

### Drug repurposing analysis

Based on the above analyses, we identified a list of targetable proteins that satisfied the following criteria: (a) significantly predicted to have altered levels based on GWAS/S-PrediXcan results, (b) significant association with COPD exacerbations, and (c) concordant direction of effect between UKBB and COPDGene. From the resulting list of proteins, we further prioritized proteins by the number of PRS__multi__-associated traits for which all 3 criteria were met. See the Supplemental Methods for further details.

### Data availability

All code used in this study is available at https://github.com/CynthiaCZ/PRSmix.git Additional data are available in the supplemental material and Supporting Data Values file. Weights for newly developed PRSs and full S-PrediXcan results are available upon request.

## Author contributions

Study design: CZ, IRK, MHC, and MRM. Acquisition, analysis, or interpretation of the data: CZ, IRK, YH, JZ, YD, BP, QC, JALS, SK, KAP, RPB, MHC, and MRM. Critical revision of the manuscript for important intellectual content: All authors. Statistical analysis: CZ, IRK, MHC, and MRM. Obtained funding: MHC, EKS, and MRM. Order of co-first authorship: CZ led and carried out the analyses, and CZ and IRK co-wrote the manuscript WBF, XH, DC, JH, MDT, JC, HKI, ESW, AM, LAL, VEO, TC, and ARM helped with interpretation of the data and critical manuscript revisions.

## Funding support

This work is the result of NIH funding, in whole or in part, and is subject to the NIH Public Access Policy. Through acceptance of this federal funding, the NIH has been given a right to make the work publicly available in PubMed Central.

- NIH grant K08 HL159318 (to MM).
- NIH grants R01 HL152728, P01 HL114501, U01 HL089856, R01 HL147148, and R01 HL133135 (to EKS).
- NIH grants R01 HL153248, R01 HL135142, R01 HL149861, R01 HL137927, and R01 HL137148 (to MHC).
- The COPD Foundation (through contributions made to an Industry Advisory Board that has included AstraZeneca, Bayer Pharmaceuticals, Boehringer-Ingelheim, Genentech, GlaxoSmithKline, Novartis, Pfizer, and Sunovion) to the COPDGene study.

## Supplementary Material

Supplemental data

ICMJE disclosure forms

Supplemental tables 1-11

Supporting data values

## Figures and Tables

**Table 1:**
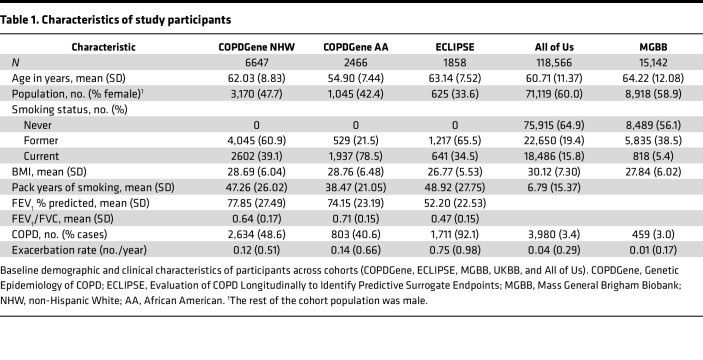
Characteristics of study participants

**Table 2:**
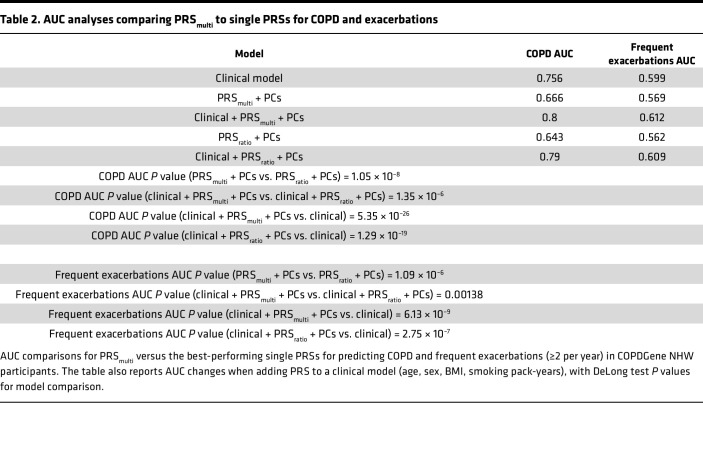
AUC analyses comparing PRS_multi_ to single PRSs for COPD and exacerbations

**Table 3:**
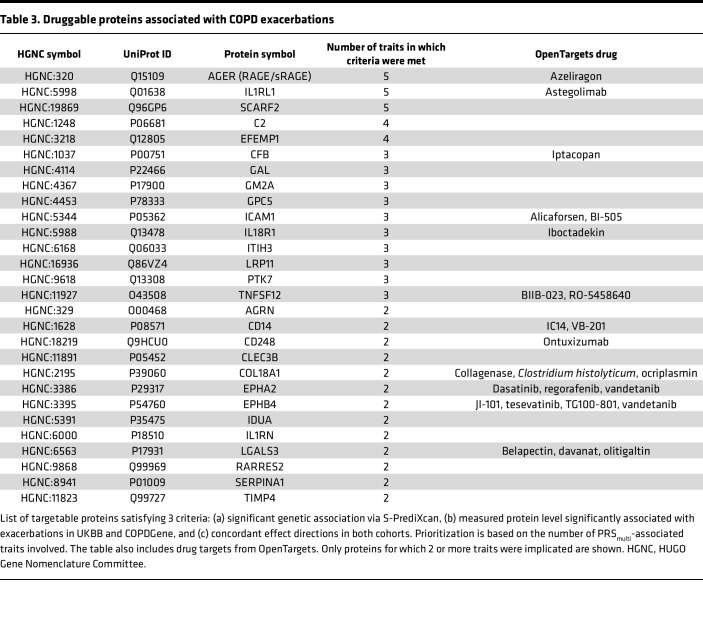
Druggable proteins associated with COPD exacerbations

## Version 1 02/19/2026

In-Press Preview

## Version 2 04/08/2026

Electronic publication
