# Supplementary material for: Multi-trait polygenic scores for COPD and COPD exacerbations implicate druggable proteins
Source: JCI Insight. 2026 Feb 19;11(7):e199951. doi: 10.1172/jci.insight.199951 (PMC13134720; doi:10.1172/jci.insight.199951)
Supplement: Supplemental data [file jciinsight-11-199951-s060.pdf]

Supplementary Appendix to “Multi-Trait Polygenic Scores for COPD and COPD Exacerbations Implicate Druggable Proteins”

Funding and Acknowledgements 2

Supplementary Methods 6

Step-by-step outline of PRS and proteomic analysis 6

Additional cohort details 7

COPDGene 7

ECLIPSE 8

Proteomic data 8

COPDGene SomaScan proteomic data 8

UK Biobank Olink proteomic data 8

Single-trait polygenic risk scores 9

Development of a multi-trait polygenic risk score 9

Meta-analysis 10

Drug repurposing analysis 11

Supplementary Discussion 11

References 12

Supplementary Figures 16

Supplementary Tables 27

Funding and Acknowledgements

COPDGene Phase 3 Grant Support and Disclaimer

The project described was supported by Award Number U01 HL089897 and Award Number U01 HL089856 from the National Heart, Lung, and Blood Institute. The content is solely the responsibility of the authors and does not necessarily represent the official views of the National Heart, Lung, and Blood Institute or the National Institutes of Health.

COPD Foundation Funding

COPDGene is also supported by the COPD Foundation through contributions made to an Industry Advisory Board that has included AstraZeneca, Bayer Pharmaceuticals, Boehringer- Ingelheim, Genentech, GlaxoSmithKline, Novartis, Pfizer, and Sunovion.

COPDGene® Investigators – Core Units

Administrative Center: James D. Crapo, MD (PI); Edwin K. Silverman, MD, PhD (PI); Barry J. Make, MD; Elizabeth A. Regan, MD, PhD

Genetic Analysis Center: Terri H. Beaty, PhD; Peter J. Castaldi, MD, MSc; Michael H. Cho, MD, MPH; Dawn L. DeMeo, MD, MPH; Adel El Boueiz, MD, MMSc; Marilyn G. Foreman, MD, MS; Auyon Ghosh, MD; Lystra P. Hayden, MD, MMSc; Craig P. Hersh, MD, MPH; Jacqueline Hetmanski, MS; Brian D. Hobbs, MD, MMSc; John E. Hokanson, MPH, PhD; Wonji Kim, PhD; Nan Laird, PhD; Christoph Lange, PhD; Sharon M. Lutz, PhD; Merry-Lynn McDonald, PhD; Dmitry Prokopenko, PhD; Matthew Moll, MD, MPH; Jarrett Morrow, PhD; Dandi Qiao, PhD;

Elizabeth A. Regan, MD, PhD; Aabida Saferali, PhD; Phuwanat Sakornsakolpat, MD; Edwin K. Silverman, MD, PhD; Emily S. Wan, MD; Jeong Yun, MD, MPH

Imaging Center: Juan Pablo Centeno; Jean-Paul Charbonnier, PhD; Harvey O. Coxson, PhD; Craig J. Galban, PhD; MeiLan K. Han, MD, MS; Eric A. Hoffman, Stephen Humphries, PhD; Francine L. Jacobson, MD, MPH; Philip F. Judy, PhD; Ella A. Kazerooni, MD; Alex Kluiber; David A. Lynch, MB; Pietro Nardelli, PhD; John D. Newell, Jr., MD; Aleena Notary; Andrea Oh, MD; Elizabeth A. Regan, MD, PhD; James C. Ross, PhD; Raul San Jose Estepar, PhD; Joyce Schroeder, MD; Jered Sieren; Berend C. Stoel, PhD; Juerg Tschirren, PhD; Edwin Van Beek, MD, PhD; Bram van Ginneken, PhD; Eva van Rikxoort, PhD; Gonzalo Vegas Sanchez- Ferrero, PhD; Lucas Veitel; George R. Washko, MD; Carla G. Wilson, MS;

PFT QA Center, Salt Lake City, UT: Robert Jensen, PhD

Data Coordinating Center and Biostatistics, *National Jewish Health, Denver, CO*: Douglas Everett, PhD; Jim Crooks, PhD; Katherine Pratte, PhD; Matt Strand, PhD; Carla G. Wilson, MS

Epidemiology Core, *University of Colorado Anschutz Medical Campus, Aurora, CO*: John E. Hokanson, MPH, PhD; Erin Austin, PhD; Gregory Kinney, MPH, PhD; Sharon M. Lutz, PhD; Kendra A. Young, PhD

Mortality Adjudication Core: Surya P. Bhatt, MD; Jessica Bon, MD; Alejandro A. Diaz, MD, MPH; MeiLan K. Han, MD, MS; Barry Make, MD; Susan Murray, ScD; Elizabeth Regan, MD; Xavier Soler, MD; Carla G. Wilson, MS

Biomarker Core: Russell P. Bowler, MD, PhD; Katerina Kechris, PhD; Farnoush Banaei- Kashani, PhD

COPDGene® Investigators – Clinical Centers

Ann Arbor VA: Jeffrey L. Curtis, MD; Perry G. Pernicano, MD

Baylor College of Medicine, Houston, TX: Nicola Hanania, MD, MS; Mustafa Atik, MD; Aladin Boriek, PhD; Kalpatha Guntupalli, MD; Elizabeth Guy, MD; Amit Parulekar, MD;

Brigham and Women's Hospital, Boston, MA: Dawn L. DeMeo, MD, MPH; Craig Hersh, MD, MPH; Francine L. Jacobson, MD, MPH; George Washko, MD

Columbia University, New York, NY: R. Graham Barr, MD, DrPH; John Austin, MD; Belinda D'Souza, MD; Byron Thomashow, MD

Duke University Medical Center, Durham, NC: Neil MacIntyre, Jr., MD; H. Page McAdams, MD; Lacey Washington, MD

HealthPartners Research Institute, Minneapolis, MN: Charlene McEvoy, MD, MPH; Joseph Tashjian, MD

Johns Hopkins University, Baltimore, MD: Robert Wise, MD; Robert Brown, MD; Nadia N. Hansel, MD, MPH; Karen Horton, MD; Allison Lambert, MD, MHS; Nirupama Putcha, MD, MHS

Lundquist Institute for Biomedical Innovation at Harbor UCLA Medical Center, Torrance, CA: Richard Casaburi, PhD, MD; Alessandra Adami, PhD; Matthew Budoff, MD; Hans Fischer, MD; Janos Porszasz, MD, PhD; Harry Rossiter, PhD; William Stringer, MD

Michael E. DeBakey VAMC, Houston, TX: Amir Sharafkhaneh, MD, PhD; Charlie Lan, DO

Minneapolis VA: Christine Wendt, MD; Brian Bell, MD; Ken M. Kunisaki, MD, MS

Morehouse School of Medicine, Atlanta, GA: Eric L. Flenaugh, MD; Hirut Gebrekristos, PhD; Mario Ponce, MD; Silanath Terpenning, MD; Gloria Westney, MD, MS

National Jewish Health, Denver, CO: Russell Bowler, MD, PhD; David A. Lynch, MB *Reliant Medical Group, Worcester, MA*: Richard Rosiello, MD; David Pace, MD

Temple University, Philadelphia, PA: Gerard Criner, MD; David Ciccolella, MD; Francis Cordova, MD; Chandra Dass, MD; Gilbert D'Alonzo, DO; Parag Desai, MD; Michael Jacobs, PharmD; Steven Kelsen, MD, PhD; Victor Kim, MD; A. James Mamary, MD; Nathaniel Marchetti, DO; Aditi Satti, MD; Kartik Shenoy, MD; Robert M. Steiner, MD; Alex Swift, MD; Irene Swift, MD; Maria Elena Vega-Sanchez, MD

University of Alabama, Birmingham, AL: Mark Dransfield, MD; William Bailey, MD; Surya P. Bhatt, MD; Anand Iyer, MD; Hrudaya Nath, MD; J. Michael Wells, MD

University of California, San Diego, CA: Douglas Conrad, MD; Xavier Soler, MD, PhD; Andrew Yen, MD

University of Iowa, Iowa City, IA: Alejandro P. Comellas, MD; Karin F. Hoth, PhD; John Newell, Jr., MD; Brad Thompson, MD

University of Michigan, Ann Arbor, MI: MeiLan K. Han, MD MS; Ella Kazerooni, MD MS; Wassim Labaki, MD MS; Craig Galban, PhD; Dharshan Vummidi, MD

University of Minnesota, Minneapolis, MN: Joanne Billings, MD; Abbie Begnaud, MD; Tadashi Allen, MD

University of Pittsburgh, Pittsburgh, PA: Frank Sciurba, MD; Jessica Bon, MD; Divay Chandra, MD, MSc; Joel Weissfeld, MD, MPH

University of Texas Health, San Antonio, San Antonio, TX: Antonio Anzueto, MD; Sandra Adams, MD; Diego Maselli-Caceres, MD; Mario E. Ruiz, MD; Harjinder Singh

Supplementary Methods

Step-by-step outline of PRS and proteomic analysis

- PRS analysis
    - Develop PRS_{multi}:
        - Calculate 25 pre-selected single-trait PRSs in COPDGene non-Hispanic White (NHW)
        - Train PRSmix+ for 4 traits: COPD, COPD exacerbations, Pi10, and emphysema in COPDGene NHW

        - Select the COPD-trained model (7 PRSs, the largest among all models) as PRS_{multi}
    - Test association of PRS_{multi} in COPDGene African American, ECLIPSE, All of Us, and MGBB and summarize via meta-analysis

- Proteomic analysis
    - Identify genetically associated proteins
        - Gather GWAS summary statistics for the 7 PRSs included in PRS_{multi}
        - Apply S-PrediXcan ARIC PredictDB protein prediction model to each GWAS
    - Identify overlapping genetically associated and measured proteins
        - For each trait, test significant S-PrediXcan proteins in COPDGene and UKB measured protein data
        - Filter for proteins significantly associated with COPD exacerbation in both cohorts and concordant in direction of effect
    - Query OpenTargets database to identify drug repurposing candidates

Additional cohort details

COPDGene

COPDGene has three completed study visits approximately 5 years apart. Pulmonary function tests were conducted at baseline enrollment as well as at 5- and 10-year follow-up visits. Genotyping was carried out using the Illumina HumanOmniExpress array, and genotyping for the Z and S alleles of the *SERPINA1* gene (which encodes alpha-1 antitrypsin, a known monogenic cause of COPD) was performed for all participants. Those with severe alpha-1 antitrypsin deficiency were excluded from the study. Imputation was conducted using the Michigan Imputation Server[1], applying the Haplotype Reference Consortium panel[2] for white participants and the 1000 Genomes Phase I v3 Cosmopolitan reference panel for African American participants[3]. Variants with an imputation quality score (r^2) ≤ 0.3 were excluded from further analysis.

ECLIPSE

Longitudinal assessments include spirometry, chest CT imaging, blood sample collection, and exacerbation tracking over a three-year period. Spirometry and exacerbation data were collected every six months during the study. Genotyping was performed using the Illumina HumanHap 550 V3 array with imputation to the Haplotype Reference Consortium panel[4].

Proteomic data

COPDGene SomaScan proteomic data

SomaScan 5K v4.0 has been measured on 5,670 participants from frozen plasma from the five-year follow-up study visit; 4,776 proteins were analyzed. Plate hybridization, median signal normalization, and plate scaling and calibration of SOMAmers were performed to control for variability across array signals, inter-run variability, inter-assay variation between analytes and batch differences between plates. Median normalization to a reference using adaptive normalization by maximum likelihood is applied within SOMAmer dilution group to quality control replicates and on individual samples to remove edge effect and technical variance. Data were log2-transformed prior to statistical analysis. Further details regarding preparation of SomaScan data has been previously published[5].

UK Biobank Olink proteomic data

Olink Explore 3072 platform has been collected on 54,219 UK Biobank participants as part of the UK Biobank Pharma Proteomics Project[6]. We included individuals from UK Biobank ≥ 40 years of age with proteomic and spirometry data. We excluded individuals with GOLD 1 COPD or

preserved ratio impaired spirometry (PRISm)[7]. To address the class imbalance between COPD cases and controls, we then performed 1:1 propensity score matching on age, sex, and pack-years of smoking using the MatchIt R package[8].

Single-trait polygenic risk scores

PRSs were obtained from the PGS Catalog for C-reactive protein (PGS003527)[9], venous thromboembolism (PGS003332)[10], type 2 diabetes (PGS000014)[11], heart failure (PGS004462)[12], hypertension (PGS002765)[13], platelet count (PGS002343)[14], obstructive sleep apnea (PGS003479)[15], neutrophil (PGS000105)[16], anxiety disorder (PGS004451)[12], cor pulmonale (PGS002049)[17], major depressive disorder (PGS002789)[18], allergic respiratory disease (PGS001109)[19], albumin (PGS002099)[17], coronary artery disease (PGS000013)[11], and respiratory infections (PGS000925)[20].

We also calculated several existing PRSs including for a deep learning model of raw spirometry data (Cosentino et al.[21]), FEV_1 and FEV_1/FVC[22], body-mass index (BMI)[23], idiopathic pulmonary fibrosis[24], and asthma[25]. For this study, we also constructed additional PRSs using the lassosum penalized regression method[26]. These included scores for emphysema[27], peak expiratory flow[28], eosinophils[29], and smoking (i.e. cigarettes per day)[30].

Development of a multi-trait polygenic risk score

PRSmix provides a framework to construct polygenic risk scores tailored for complex traits with sub-phenotypic variation[31]. PRSmix+ extends this approach by incorporating phenotype

stratification and optimizing score construction through meta-modeling, addressing challenges such as phenotypic heterogeneity and population diversity. By incorporating both primary and secondary phenotypes, PRSmix+ has demonstrated improved performance for multifactorial diseases[31]. This approach is especially suited to COPD, a condition with heterogeneous clinical presentations and phenotypic overlap.

We calculated Pearson correlation coefficients between PRSs (including PRS_{multi}) in COPD NHW individuals and performed hierarchical clustering and visualized results with a heatmap to visually assess correlation structure amongst PRSs.

We performed area under the receiver operating characteristic curve (AUC) analyses using the pROC R package[32]. We compared a clinical model (age, sex, pack-years of smoking, BMI, and genetic principal components), PRS_{multi}, and the highest weighted individual PRS identified by PRSmix+, and combined models with PRSs and clinical covariates. For each single-trait PRS and the $PRS_{FEV1+FEV1/FVC}$ from Moll et al., we compared the AUC for predicting COPD status and frequent exacerbations to the PRS_{multi}. We also compared AUCs for the protein biomarkers implicated by the most number of traits contributing to PRS_{multi} (Table 3, column 4) for predicting frequent exacerbations in multivariable models. Statistical comparisons between models were performed using DeLong's test, with p-values < 0.05 considered significant.

Meta-analysis

As an additional sensitivity analysis, we conducted a leave-one-out meta-analysis excluding COPDGene NHW, since this cohort was used to develop the PRS_{multi} weights and we aimed to ensure overfitting was not the primary driver of observed effects.

Drug repurposing analysis

For example, a protein meeting criterion for both FEV_1 and FEV_1/FVC would be ranked higher than one associated with only a single trait. Each protein was cross-referenced with the OpenTargets database to identify corresponding druggable targets[33], existing drugs, and ongoing or completed clinical trials. We also identified proteins in which the association of each protein level with COPD affection status, frequent exacerbations ($\geq$ 2 exacerbations/year vs. no exacerbations), and highest vs. lowest quintile of the PRS_{multi} in COPDGene NHW participants was consistent across all three outcomes.

Supplementary Discussion

Previously, we showed that individuals with low BMI disproportionate to their genetically-predicted BMI (e.g., COPD patients with cachexia) have higher mortality risk[23]. In the current study, PRS_{BMI} was strongly associated with exacerbations in biobank cohorts, though it did not reach significance in meta-analyses, largely due to a flipped effect in COPDGene African Americans. Whether these discrepancies reflect differences in COPD definitions, ascertainment, or cross ancestry genetic prediction remains unclear. Given the links between BMI, mortality, and exacerbations, further investigation is needed.

The inclusion of PRS_{IPF} in PRS_{multi} is consistent with prior evidence that IPF and COPD share overlapping and distinct genetic loci[34]. Smoking and C-reactive protein, both associated with COPD progression and inflammation, explain their inclusion in PRS_{multi}[35-37].

This study builds upon prior work in several key ways. Unlike our prior work on PRSs for COPD, which focused on just two spirometry traits without incorporating negative binomial modeling for

exacerbations or expanding analyses to biobanks, our work addresses these gaps. He et al. applied a multi-trait GWAS framework that included genetic associations for asthma, COPD, lung cancer, smoking, and spirometry traits[38]; we significantly expanded this list including 25 traits based on clinician input and literature review of COPD comorbidities, risk factors for exacerbations, and existing biomarkers. We then utilized PRSmix+ to identify relevant traits. In one of these two prior studies, either spirometry-defined COPD in research cohorts or ICD-defined COPD in biobank cohorts were tested, but we tested both definitions together and observed robust but highly heterogeneous associations across cohorts. Finally, we placed a stronger emphasis on exacerbations, carefully defining our phenotype and utilized more advanced negative binomial models compared to simple linear or logistic regression models. Taken together, our study provides a more robust foundation for understanding the shared genetic architecture of COPD and the relationship to exacerbations.

References

1. Das, S., Forer, L., Schönherr, S., Sidore, C., Locke, A.E., Kwong, A., Vrieze, S.I., Chew, E.Y., Levy, S., McGue, M., et al. (2016). Next-generation genotype imputation service and methods. Nature Genetics *48*, 1284-1287. 10.1038/ng.3656.
2. A reference panel of 64,976 haplotypes for genotype imputation. (2016). Nature Genetics *48*, 1279-1283. 10.1038/ng.3643.
3. Fairley, S., Lowy-Gallego, E., Perry, E., and Flicek, P. (2020). The International Genome Sample Resource (IGSR) collection of open human genomic variation resources. Nucleic Acids Research *48*, D941-D947. 10.1093/nar/gkz836.
4. Loh, P.-R., Danecek, P., Palamara, P.F., Fuchsberger, C., A Reshef, Y., K Finucane, H., Schoenherr, S., Forer, L., McCarthy, S., Abecasis, G.R., et al. (2016). Reference-based phasing using the Haplotype Reference Consortium panel. Nature Genetics *48*, 1443-1448. 10.1038/ng.3679.
5. Hill, A.C., Guo, C., Litkowski, E.M., Manichaikul, A.W., Yu, B., Konigsberg, I.R., Gorbet, B.A., Lange, L.A., Pratte, K.A., Kechris, K.J., et al. (2023). Large scale proteomic studies create novel privacy considerations. Sci Rep *13*, 9254. 10.1038/s41598-023-34866-6.
6. Sun, B.B., Chiou, J., Traylor, M., Benner, C., Hsu, Y.-H., Richardson, T.G., Surendran, P., Mahajan, A., Robins, C., Vasquez-Grinnell, S.G., et al. (2023). Plasma proteomic

associations with genetics and health in the UK Biobank. Nature *622*, 329-338. 10.1038/s41586-023-06592-6.

7. Wan, E.S., Castaldi, P.J., Cho, M.H., Hokanson, J.E., Regan, E.A., Make, B.J., Beaty, T.H., Han, M.K., Curtis, J.L., Curran-Everett, D., et al. (2014). Epidemiology, genetics, and subtyping of preserved ratio impaired spirometry (PRISm) in COPDGene. Respir Res *15*, 89. 10.1186/s12931-014-0089-y.
8. Ho, D.E., Imai, K., King, G., and Stuart, E.A. (2011). MatchIt: Nonparametric Preprocessing for Parametric Causal Inference. Journal of Statistical Software *42*. 10.18637/jss.v042.i08.
9. Ding, Y., Hou, K., Xu, Z., Pimplaskar, A., Petter, E., Boulier, K., Privé, F., Vilhjálmsson, B.J., Olde Loohuis, L.M., and Pasaniuc, B. (2023). Polygenic scoring accuracy varies across the genetic ancestry continuum. Nature *618*, 774-781. 10.1038/s41586-023-06079-4.
10. Ghouse, J., Tragante, V., Ahlberg, G., Rand, S.A., Jespersen, J.B., Leinøe, E.B., Vissing, C.R., Trudsø, L., Jonsdottir, I., Banasik, K., et al. (2023). Genome-wide meta-analysis identifies 93 risk loci and enables risk prediction equivalent to monogenic forms of venous thromboembolism. Nature Genetics *55*, 399-409. 10.1038/s41588-022-01286-7.
11. Khera, A.V., Chaffin, M., Aragam, K.G., Haas, M.E., Roselli, C., Choi, S.H., Natarajan, P., Lander, E.S., Lubitz, S.A., Ellinor, P.T., and Kathiresan, S. (2018). Genome-wide polygenic scores for common diseases identify individuals with risk equivalent to monogenic mutations. Nat Genet *50*, 1219-1224. 10.1038/s41588-018-0183-z.
12. Jung, H., Jung, H.-U., Baek, E.J., Kwon, S.Y., Kang, J.-O., Lim, J.E., and Oh, B. (2024). Integration of risk factor polygenic risk score with disease polygenic risk score for disease prediction. Communications Biology *7*. 10.1038/s42003-024-05874-7.
13. Mars, N., Lindbohm, J.V., della Briotta Parolo, P., Widén, E., Kaprio, J., Palotie, A., and Ripatti, S. (2022). Systematic comparison of family history and polygenic risk across 24 common diseases. The American Journal of Human Genetics *109*, 2152-2162. 10.1016/j.ajhg.2022.10.009.
14. Weissbrod, O., Kanai, M., Shi, H., Gazal, S., Peyrot, W.J., Khera, A.V., Okada, Y., Matsuda, K., Yamanashi, Y., Furukawa, Y., et al. (2022). Leveraging fine-mapping and multipopulation training data to improve cross-population polygenic risk scores. Nature Genetics *54*, 450-458. 10.1038/s41588-022-01036-9.
15. Zhang, Y., Elgart, M., Kurniansyah, N., Spitzer, B.W., Wang, H., Kim, D., Shah, N., Daviglus, M., Zee, P.C., Cai, J., et al. (2022). Genetic determinants of cardiometabolic and pulmonary phenotypes and obstructive sleep apnoea in HCHS/SOL. eBioMedicine *84*. 10.1016/j.ebiom.2022.104288.
16. Xu, Y., Vuckovic, D., Ritchie, S.C., Akbari, P., Jiang, T., Grealey, J., Butterworth, A.S., Ouwehand, W.H., Roberts, D.J., Di Angelantonio, E., et al. (2022). Machine learning optimized polygenic scores for blood cell traits identify sex-specific trajectories and genetic correlations with disease. Cell Genomics *2*. 10.1016/j.xgen.2021.100086.
17. Prive, F., Aschard, H., Carmi, S., Folkersen, L., Hoggart, C., O'Reilly, P.F., and Vilhjalmsson, B.J. (2022). Portability of 245 polygenic scores when derived from the UK Biobank and applied to 9 ancestry groups from the same cohort. Am J Hum Genet *109*, 373. 10.1016/j.ajhg.2022.01.007.
18. Gui, Y., Zhou, X., Wang, Z., Zhang, Y., Wang, Z., Zhou, G., Zhao, Y., Liu, M., Lu, H., and Zhao, H. (2022). Sex-specific genetic association between psychiatric disorders and

cognition, behavior and brain imaging in children and adults. Translational Psychiatry *12*. 10.1038/s41398-022-02041-6.

19. Ripatti, S., Tanigawa, Y., Qian, J., Venkataraman, G., Justesen, J.M., Li, R., Tibshirani, R., Hastie, T., and Rivas, M.A. (2022). Significant sparse polygenic risk scores across 813 traits in UK Biobank. PLOS Genetics *18*. 10.1371/journal.pgen.1010105.
20. Lambert, S.A., Gil, L., Jupp, S., Ritchie, S.C., Xu, Y., Buniello, A., McMahon, A., Abraham, G., Chapman, M., Parkinson, H., et al. (2021). The Polygenic Score Catalog as an open database for reproducibility and systematic evaluation. Nat Genet *53*, 420-425. 10.1038/s41588-021-00783-5.
21. Cosentino, J., Behsaz, B., Alipanahi, B., McCaw, Z.R., Hill, D., Schwantes-An, T.-H., Lai, D., Carroll, A., Hobbs, B.D., Cho, M.H., et al. (2023). Inference of chronic obstructive pulmonary disease with deep learning on raw spirograms identifies new genetic loci and improves risk models. Nature Genetics *55*, 787-795. 10.1038/s41588-023-01372-4.
22. Moll, M., Sakornsakolpat, P., Shrine, N., Hobbs, B.D., DeMeo, D.L., John, C., Guyatt, A.L., McGeachie, M.J., Gharib, S.A., Obeidat, M., et al. (2020). Chronic obstructive pulmonary disease and related phenotypes: polygenic risk scores in population-based and case-control cohorts. Lancet Respir Med *8*, 696-708. 10.1016/S2213-2600(20)30101-6.
23. Zhang, J., Moll, M., Hobbs, B.D., Bakke, P., Regan, E.A., Xu, H., Dupuis, J., Chiles, J.W., McDonald, M.-L.N., Divo, M.J., et al. (2024). Genetically Predicted Body Mass Index and Mortality in Chronic Obstructive Pulmonary Disease. American Journal of Respiratory and Critical Care Medicine *210*, 890-899. 10.1164/rccm.202308-1384OC.
24. Moll, M., Peljto, A.L., Kim, J.S., Xu, H., Debban, C.L., Chen, X., Menon, A., Putman, R.K., Ghosh, A.J., Saferali, A., et al. (2023). A Polygenic Risk Score for Idiopathic Pulmonary Fibrosis and Interstitial Lung Abnormalities. American Journal of Respiratory and Critical Care Medicine *208*, 791-801. 10.1164/rccm.202212-2257OC.
25. Moll, M., Sordillo, J.E., Ghosh, A.J., Hayden, L.P., McDermott, G., McGeachie, M.J., Dahlin, A., Tiwari, A., Manmadkar, M.G., Abston, E.D., et al. (2023). Polygenic risk scores identify heterogeneity in asthma and chronic obstructive pulmonary disease. Journal of Allergy and Clinical Immunology *152*, 1423-1432. 10.1016/j.jaci.2023.08.002.
26. Mak, T.S.H., Porsch, R.M., Choi, S.W., Zhou, X., and Sham, P.C. (2017). Polygenic scores via penalized regression on summary statistics. Genetic Epidemiology *41*, 469-480. 10.1002/gepi.22050.
27. Cho, M.H., McDonald, M.L., Zhou, X., Mattheisen, M., Castaldi, P.J., Hersh, C.P., Demeo, D.L., Sylvia, J.S., Ziniti, J., Laird, N.M., et al. (2014). Risk loci for chronic obstructive pulmonary disease: a genome-wide association study and meta-analysis. Lancet Respir Med *2*, 214-225. 10.1016/S2213-2600(14)70002-5.
28. Shrine, N., Guyatt, A.L., Erzurumluoglu, A.M., Jackson, V.E., Hobbs, B.D., Melbourne, C.A., Batini, C., Fawcett, K.A., Song, K., Sakornsakolpat, P., et al. (2019). New genetic signals for lung function highlight pathways and chronic obstructive pulmonary disease associations across multiple ancestries. Nat Genet *51*, 481-493. 10.1038/s41588-018-0321-7.
29. Astle, W.J., Elding, H., Jiang, T., Allen, D., Ruklisa, D., Mann, A.L., Mead, D., Bouman, H., Riveros-Mckay, F., Kostadima, M.A., et al. (2016). The Allelic Landscape of Human Blood Cell Trait Variation and Links to Common Complex Disease. Cell *167*, 1415-1429 e1419. 10.1016/j.cell.2016.10.042.

30. Liu, M., Jiang, Y., Wedow, R., Li, Y., Brazel, D.M., Chen, F., Datta, G., Davila-Velderrain, J., McGuire, D., Tian, C., et al. (2019). Association studies of up to 1.2 million individuals yield new insights into the genetic etiology of tobacco and alcohol use. Nature Genetics *51*, 237-244. 10.1038/s41588-018-0307-5.
31. Truong, B., Hull, L.E., Ruan, Y., Huang, Q.Q., Hornsby, W., Martin, H., van Heel, D.A., Wang, Y., Martin, A.R., Lee, S.H., and Natarajan, P. (2024). Integrative polygenic risk score improves the prediction accuracy of complex traits and diseases. Cell Genomics *4*. 10.1016/j.xgen.2024.100523.
32. Robin, X., Turck, N., Hainard, A., Tiberti, N., Lisacek, F., Sanchez, J.-C., and Müller, M. (2011). pROC: an open-source package for R and S+ to analyze and compare ROC curves. BMC Bioinformatics *12*. 10.1186/1471-2105-12-77.
33. Ochoa, D., Hercules, A., Carmona, M., Suveges, D., Baker, J., Malangone, C., Lopez, I., Miranda, A., Cruz-Castillo, C., Fumis, L., et al. (2023). The next-generation Open Targets Platform: reimagined, redesigned, rebuilt. Nucleic Acids Research *51*, D1353-D1359. 10.1093/nar/gkac1046.
34. Hobbs, B.D., de Jong, K., Lamontagne, M., Bosse, Y., Shrine, N., Artigas, M.S., Wain, L.V., Hall, I.P., Jackson, V.E., Wyss, A.B., et al. (2017). Genetic loci associated with chronic obstructive pulmonary disease overlap with loci for lung function and pulmonary fibrosis. Nat Genet *49*, 426-432. 10.1038/ng.3752.
35. Viniol, C., and Vogelmeier, C.F. (2018). Exacerbations of COPD. Eur Respir Rev *27*. 10.1183/16000617.0103-2017.
36. Agusti, A., Edwards, L.D., Rennard, S.I., MacNee, W., Tal-Singer, R., Miller, B.E., Vestbo, J., Lomas, D.A., Calverley, P.M., Wouters, E., et al. (2012). Persistent systemic inflammation is associated with poor clinical outcomes in COPD: a novel phenotype. PLoS One *7*, e37483. 10.1371/journal.pone.0037483.
37. Chen, Y.W., Leung, J.M., and Sin, D.D. (2016). A Systematic Review of Diagnostic Biomarkers of COPD Exacerbation. PLoS One *11*, e0158843. 10.1371/journal.pone.0158843.
38. He, Y., Lu, W., Jee, Y.H., Wang, Y., Tsuo, K., Qian, D.C., Diao, J.A., Huang, H., Patel, C.J., Byun, J., et al. (2024). 10.1101/2024.08.25.24312558.

Supplementary Figures

Supplementary Figure 1. Heatmap showing hierarchical clustering of Pearson correlation coefficients between PRSs input into PRSmix+.

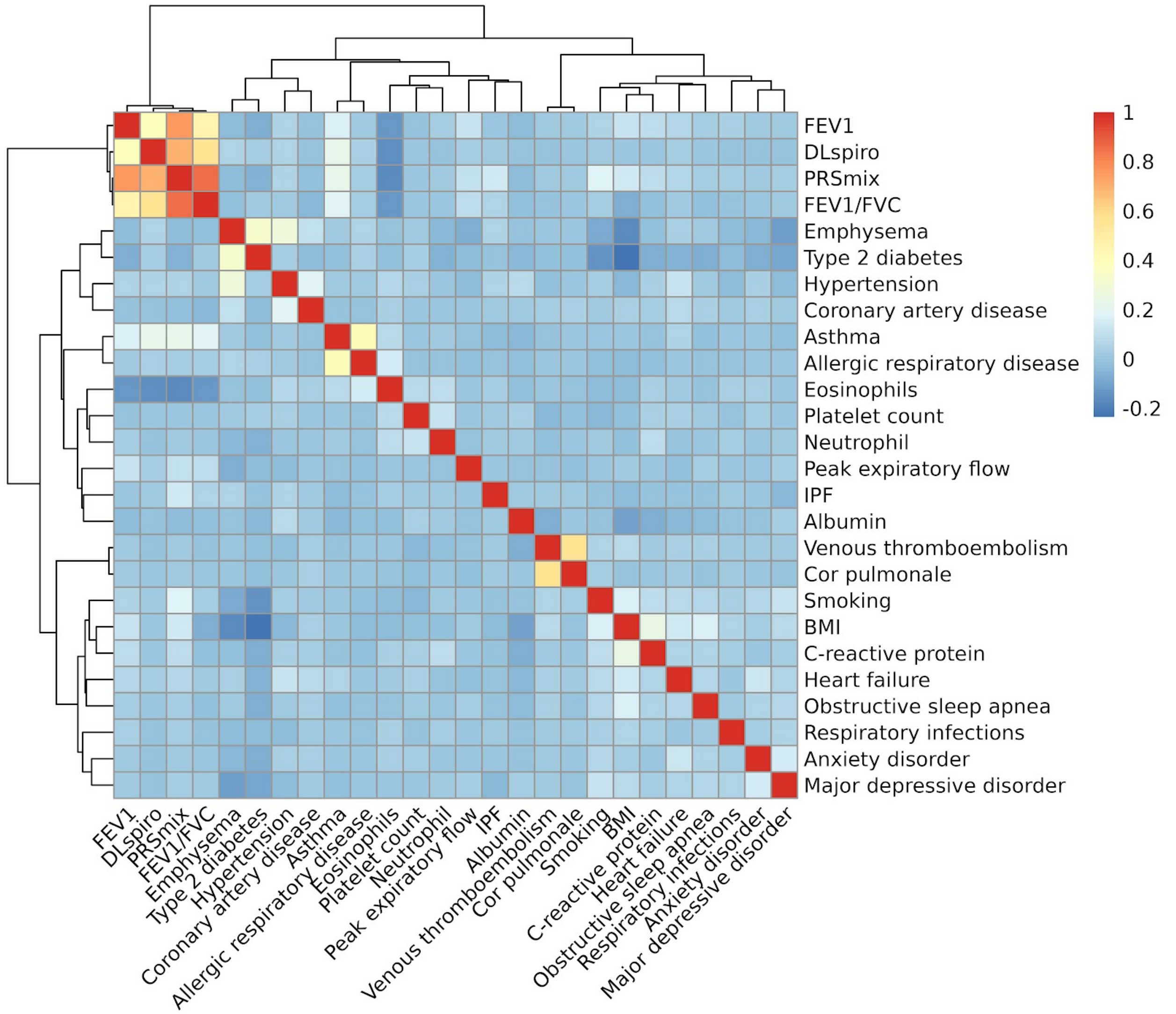

Supplementary Figure 2. Forest plot showing the random effects meta-analysis of multivariable negative binomial associations of PRS_{BMI} on exacerbations.

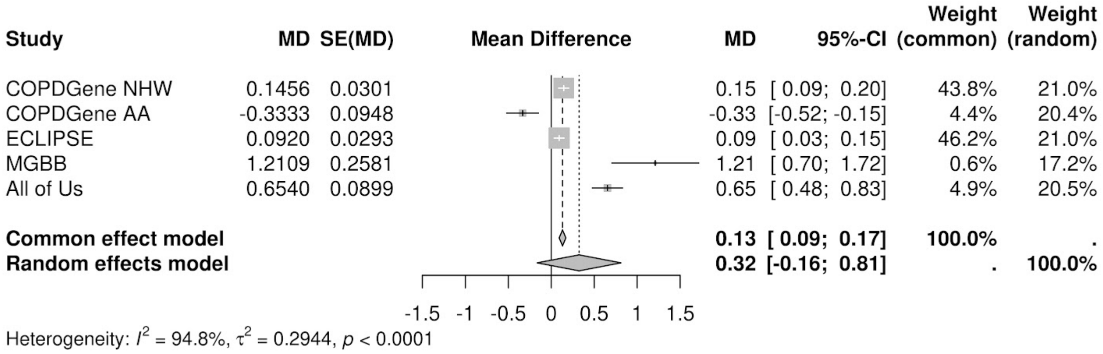

Supplementary Figure 3. Funnel plots of meta-analysis results for COPD and exacerbations. Funnel plots displaying meta-analysis results for (A) COPD and (B) COPD exacerbations.

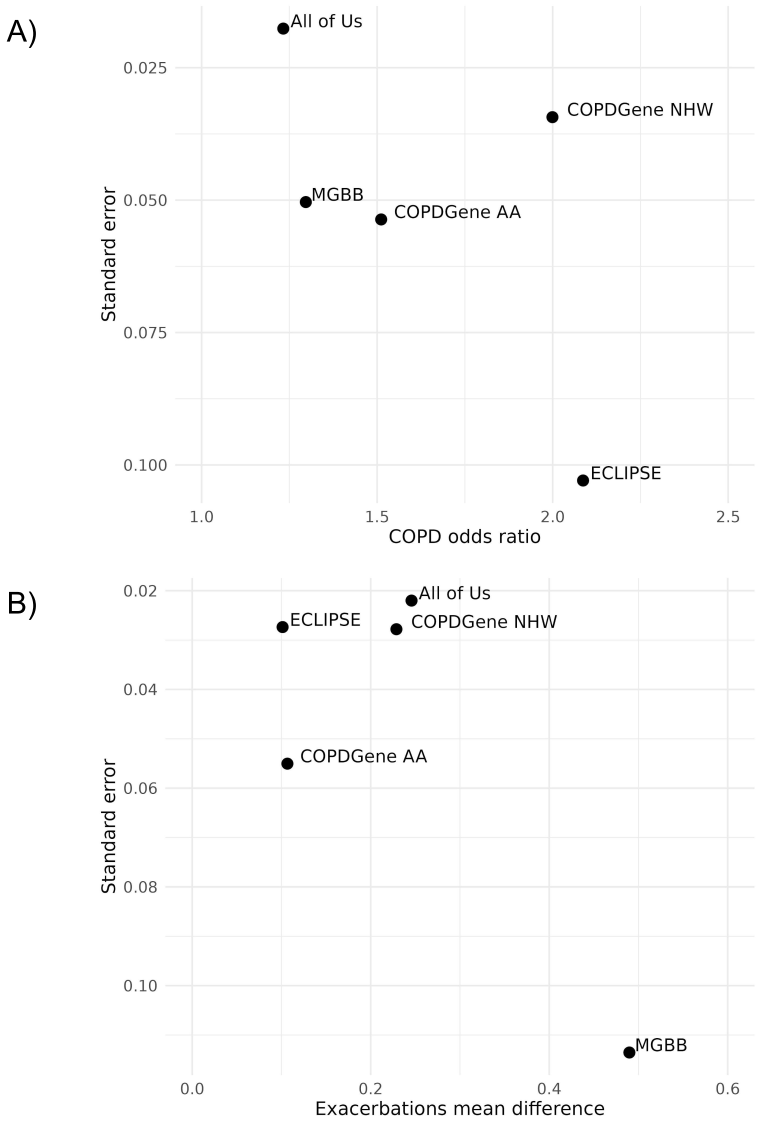

Supplementary Figure 4. Leave-one-out meta-analysis for COPD (A) and exacerbations (B). Meta-analyses excluding COPDGene NHW participants were performed as this cohort was used to tune PRS_{multi} weights and could be prone to overfitting.

A) Leave-one-out meta-analysis: COPD outcome

Study	logOR	SE(logOR)	OR	95%-CI	Weight (common)	Weight (random)
COPDGene AA	0.4131	0.0536	1.51	[1.36; 1.68]	8.6%	25.5%
ECLIPSE	0.7356	0.1029	2.09	[1.71; 2.55]	2.3%	22.0%
MGBB	0.2599	0.0504	1.30	[1.17; 1.43]	9.7%	25.7%
All of Us	0.2092	0.0176	1.23	[1.19; 1.28]	79.4%	26.9%
**Common effect model**			**1.28**	**[1.24; 1.32]**	**100.0%**	**.**
**Random effects model**			**1.48**	**[1.19; 1.84]**	**.**	**100.0%**

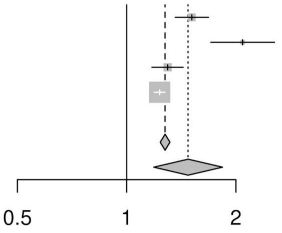

Heterogeneity: $I^2 = 91.8\%$, $\tau^2 = 0.0458$, $p < 0.0001$

B) Leave-one-out meta-analysis: COPD Exacerbations Outcome

Study	MD	SE(MD)	MD	95%-CI	Weight (common)	Weight (random)
COPDGene AA	0.1067	0.0550	0.11	[-0.00; 0.21]	8.7%	25.4%
ECLIPSE	0.1013	0.0274	0.10	[0.05; 0.15]	35.1%	28.3%
MGBB	0.4898	0.1136	0.49	[0.27; 0.71]	2.0%	17.6%
All of Us	0.2457	0.0220	0.25	[0.20; 0.29]	54.2%	28.7%
**Common effect model**			**0.19**	**[0.16; 0.22]**	**100.0%**	**.**
**Random effects model**			**0.21**	**[0.07; 0.36]**	**.**	**100.0%**

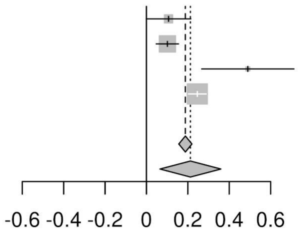

Heterogeneity: $I^2 = 88.5\%$, $\tau^2 = 0.0192$, $p < 0.0001$

Supplementary Figure 5. ROC curves for PRS-based models of COPD and exacerbations. Receiver operating characteristic (ROC) curves for models predicting (A) COPD and (B) exacerbations. Models include combinations of clinical covariates, PRS_{multi}, PRS_{ratio}, and genetic principal components.

A)

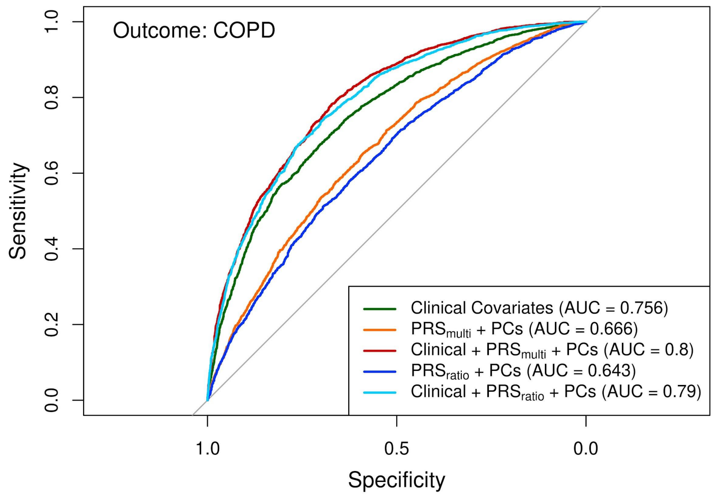

B)

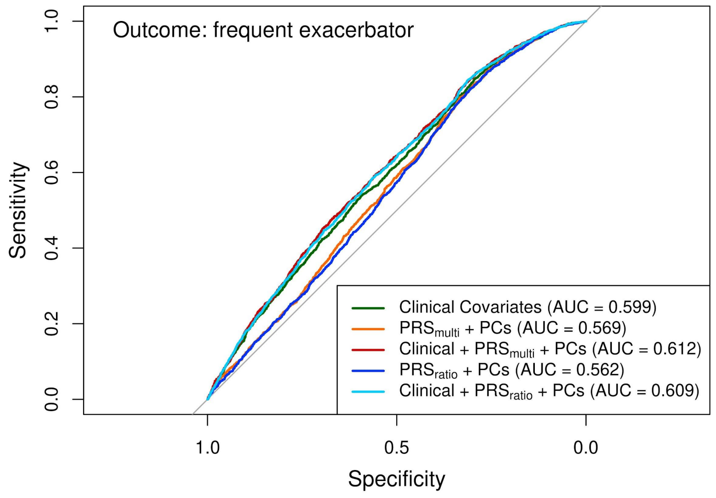

Supplementary Figure 6. Protein Associations with COPD Outcomes. Of the 73 genetically-predicted proteins identified to be associated with COPD exacerbations, eight proteins were associated with COPD affection status (left), frequent exacerbations (≥ 2 exacerbations/year vs. no exacerbations) (middle), and significantly differentially expressed between the top and bottom quintile of the PRS_{multi} (right) in COPDGene NHW participants. Boxplots for each association with interquartile ranges are displayed. P-values for Student t-tests are also displayed.

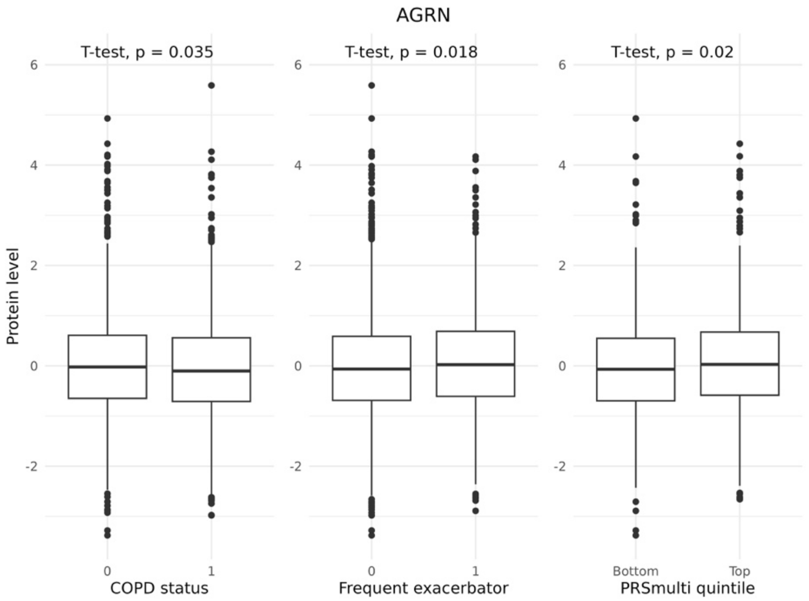

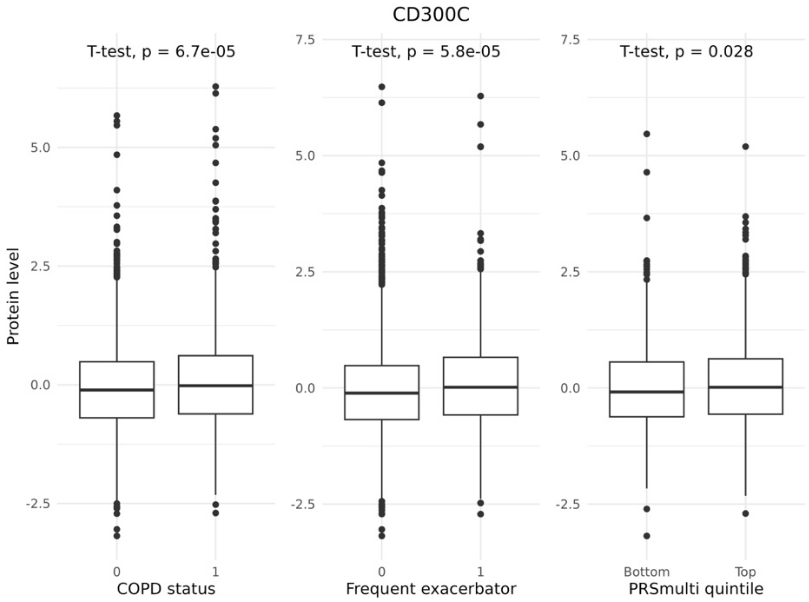

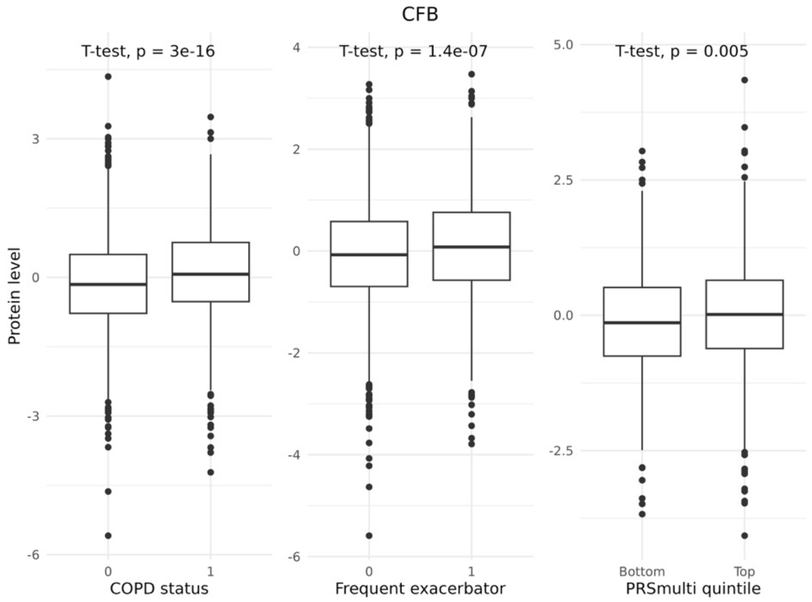
CFB
T-test, p = 3e-16
T-test, p = 1.4e-07
T-test, p = 0.005
Protein level
COPD status
Frequent exacerbator
PRSmulti quintile
Bottom
Top

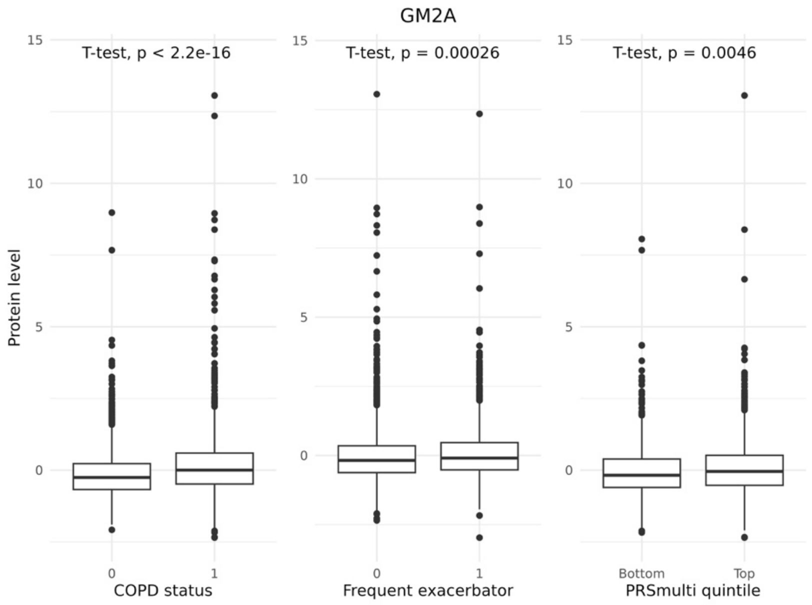
GM2A
T-test, p < 2.2e-16
T-test, p = 0.00026
T-test, p = 0.0046
Protein level
COPD status
Frequent exacerbator
PRSmulti quintile
Bottom
Top

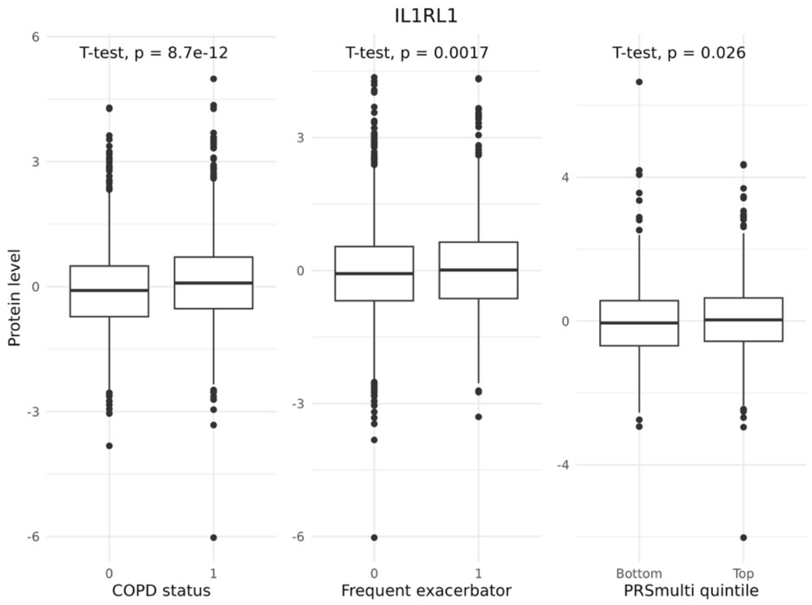
IL1RL1
T-test, p = 8.7e-12
T-test, p = 0.0017
T-test, p = 0.026
Protein level
COPD status
Frequent exacerbator
PRSmulti quintile
Bottom
Top

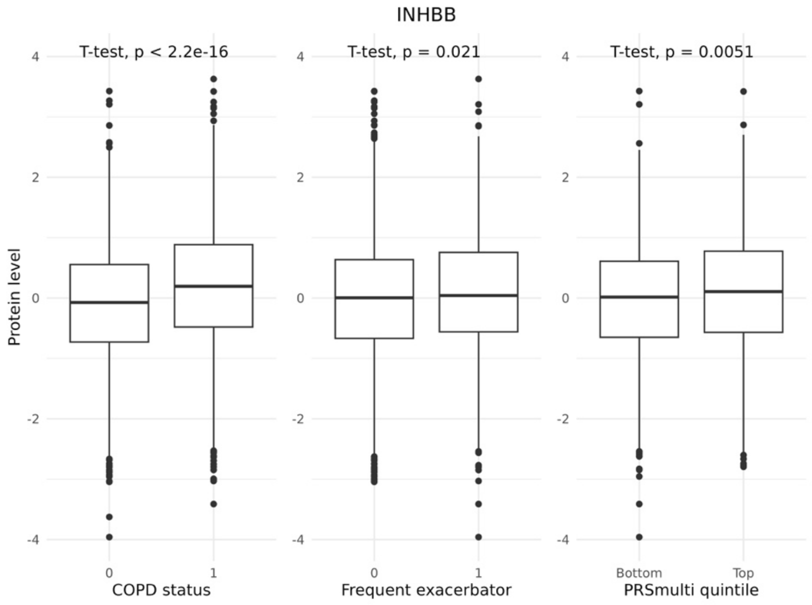
INHBB
T-test, p < 2.2e-16
T-test, p = 0.021
T-test, p = 0.0051
Protein level
COPD status
Frequent exacerbator
PRSmulti quintile
Bottom
Top

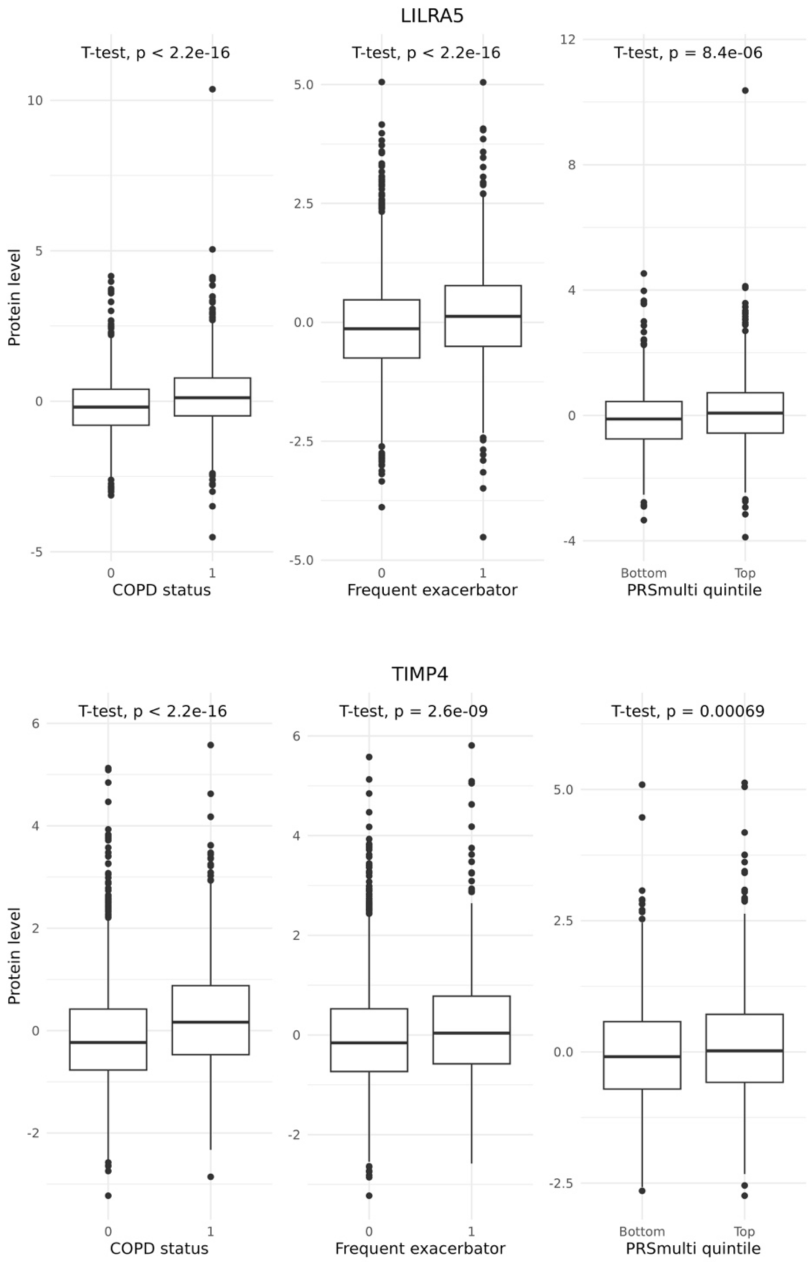

LILRA5
T-test, p < 2.2e-16
T-test, p < 2.2e-16
T-test, p = 8.4e-06
Protein level
COPD status
Frequent exacerbator
PRSmulti quintile
Bottom
Top
TIMP4
T-test, p < 2.2e-16
T-test, p = 2.6e-09
T-test, p = 0.00069
Protein level
COPD status
Frequent exacerbator
PRSmulti quintile
Bottom
Top

Supplementary Figure 7. UK Biobank CONSORT diagram.

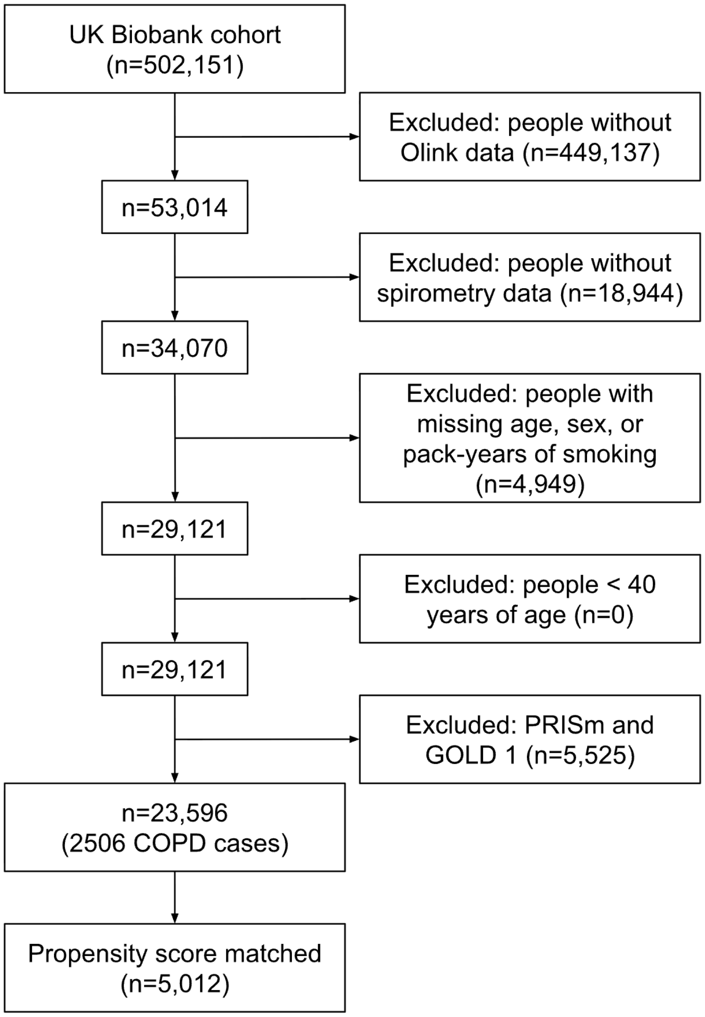

Supplementary Figure 8. ROC curves comparing multivariable models predicting frequent exacerbators (≥2 exacerbations per year) for the PRS_{multi} and SomaScan aptamer levels of IL1RL1, RAGE/sRAGE, and SCARF2 in COPDGene NHW participants. Models were adjusted for age, sex, race, smoking pack-years, BMI, and COPD case-control status.

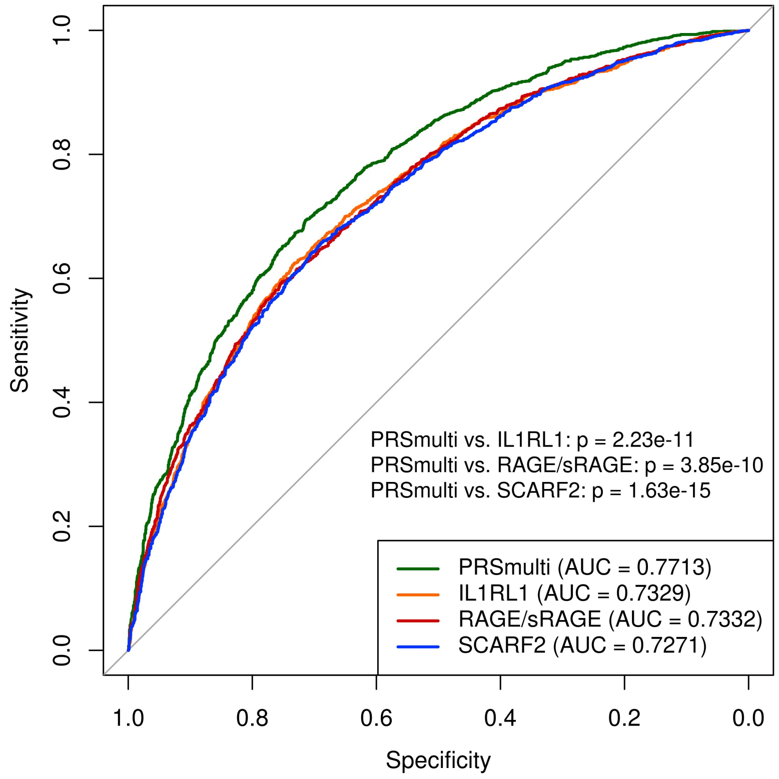

Supplementary Tables

Supplementary Table 1. Characteristics of study participants with proteomics data. Summary statistics for participants with available plasma proteomics data from COPDGene and UKBB.

Characteristic	*COPDGene*	*UK Biobank*
N	5173	5012
Age in years (mean (sd))	65.42 (8.81)	59.87 (7.17)
Sex (No. % Female)	2626 (50.8)	2178 (43.5)
Race (No. %)		
Non-Hispanic White	3828 (74.0)	4990 (99.6)
African American	1345 (26.0)	22 (0.4)
smoking status (No. %)		
Never	0	1993 (39.8)
Former	3448 (66.7)	2070 (41.3)
Current	1723 (33.3)	949 (18.9)
Pack years of smoking (mean (sd))	40.65 (25.35)	19.41 (22.74)
FEV1 % predicted (mean (sd))	80.34 (25.55)	81.49 (20.39)
FEV1/FVC (mean (sd))	0.68 (0.15)	0.70 (0.09)
Exacerbation rate (No./year)	0.11 (0.55)	0.10 (0.42)
COPD (No. % Cases)	1615 (41.1)	2506 (50.0)

Supplementary Table 2. PRSs included in PRSmix+ analysis. List of PRSs used in PRSmix+ modeling, along with their source (GWAS, PGS Catalog, or newly developed) and references.

	Trait	PGS catalog ID	PRS or GWAS PubMed link
1	FEV1/FVC	NA	https://pubmed.ncbi.nlm.nih.gov/32649918/
2	FEV1	NA	https://pubmed.ncbi.nlm.nih.gov/32649918/
3	Emphysema	NA	https://pubmed.ncbi.nlm.nih.gov/24621683/
4	Body-mass index	NA	https://pubmed.ncbi.nlm.nih.gov/38471013/
5	Deep learning spirometry (DLspiro)	NA	https://www.nature.com/articles/s41588-023-01388-w
6	Idiopathic pulmonary fibrosis	NA	https://pubmed.ncbi.nlm.nih.gov/37523715/
7	Peak expiratory flow	NA	https://pmc.ncbi.nlm.nih.gov/articles/PMC10011137/
8	Smoking (cigarettes per day)	NA	https://pmc.ncbi.nlm.nih.gov/articles/PMC6358542/
9	C-reactive protein	PGS003527	https://pubmed.ncbi.nlm.nih.gov/37198491/
10	Venous thromboembolism	PGS003332	https://pubmed.ncbi.nlm.nih.gov/36658437/
11	Type 2 diabetes	PGS000014	https://pubmed.ncbi.nlm.nih.gov/30104762/
12	Heart failure	PGS004462	https://pubmed.ncbi.nlm.nih.gov/38351177/
13	Hypertension	PGS002765	https://pubmed.ncbi.nlm.nih.gov/36347255/
14	Platelet count	PGS002343	https://pubmed.ncbi.nlm.nih.gov/35393596/
15	Obstructive sleep apnea	PGS003479	https://pubmed.ncbi.nlm.nih.gov/36174398/
16	Neutrophil	PGS000105	https://www.ncbi.nlm.nih.gov/pubmed/?term=27863252
17	Anxiety disorder	PGS004451	https://pubmed.ncbi.nlm.nih.gov/38351177/
18	Asthma	NA	https://pubmed.ncbi.nlm.nih.gov/37595761/
19	Cor pulmonale	PGS002049	https://pubmed.ncbi.nlm.nih.gov/34995502/
20	Major depressive disorder	PGS002789	https://pubmed.ncbi.nlm.nih.gov/36028495/
21	Allergic respiratory disease	PGS001109	https://pubmed.ncbi.nlm.nih.gov/35324888/
22	Albumin	PGS002099	https://pubmed.ncbi.nlm.nih.gov/34995502/
23	Coronary artery disease	PGS000013	https://pubmed.ncbi.nlm.nih.gov/30104762/
24	Eosinophils	NA	https://www.ncbi.nlm.nih.gov/pubmed/?term=27863252
25	Respiratory infections	PGS000925	https://pubmed.ncbi.nlm.nih.gov/35324888/

Supplementary Table 3. PRS-mix weights for COPDGene, weighted by different phenotypes.

Summary of PRS-mix weights assigned to different traits in the construction of PRS_{multi}. This table presents the contribution of each PRS to the final model predicting COPD and exacerbations.

**PRS**	**Trait used to weight PRS-mix**			
	Exacerbations	*Emphysema (% LAA < -950 HU)*	*Airway thickness measure (Pi10)*	*COPD case-control status*
FEV1/FVC	0.0599	-0.105	0.105	0.245
FEV1	0	0	0.129	0.178
Deep learning spirometry (DLspiro)	0.0212	-0.0183	0.0402	0.107
Smoking (cigarettes per day)	0	0	0	0.0526
Idiopathic pulmonary fibrosis	0	0	0	0.0497
BMI	0	0	0.094	0.0466
C-reactive protein	0	0	0.0299	0.017
Emphysema	0	0	0	0
Peak expiratory flow	0	0	0	0
Venous thromboembolism	0	0	0	0
Type 2 diabetes	0	0	0	0
Heart failure	0	0	0	0
Hypertension	0	0	0	0
Platelet count	0	0	0	0
Obstructive sleep apnea	0	0	0	0
Neutrophil	0	0	0	0
Anxiety disorder	0	0	0	0
Asthma	0	0	0	0
Cor pulmonale	0	0	0	0
Major depressive disorder	0	0	0	0
Allergic respiratory disease	0	0	0	0
Albumin	0	0	0	0
Coronary artery disease	0	0	0	0
Eosinophil	0	0	0	0
Respiratory infections	0	0	0	0

Supplementary Table 4. Association of PRSmix+ values with different weights in COPDGene. Results of multivariable models testing PRSmix+ scores with different weights for their association with COPD and exacerbations.

PRS	Outcome							
	**Exacerbations**		**Adjusted lung density**		**Pi10**		**COPD**	
	beta (95% CI)	*p-value*	*beta (95% CI)*	*p-value*	*beta (95% CI)*	*p-value*	*beta (95% CI)*	*p-value*
PRS mix (Exacerbation weights)	0.195 (0.135, 0.254)	2.47E-12	-2.259 (-2.893, -1.626)	3.19E-12	0.113 (0.098, 0.127)	8.66E-54	0.621 (0.556, 0.688)	1.86E-75
PRS mix (Adjust lung density weights)	-0.193 (-0.252, -0.133)	3.82E-12	2.263 (1.629, 2.896)	2.96E-12	-0.111 (-0.125, -0.097)	2.47E-52	-0.618 (-0.684, -0.552)	1.54E-74
PRS mix (Pi10 weights)	0.245 (0.186, 0.305)	2.61E-18	-1.919 (-2.568, -1.270)	7.20E-09	0.148 (0.133, 0.162)	4.84E-90	0.668 (0.602, 0.736)	5.31E-85
PRS mix (COPD weights)	0.229 (0.170, 0.288)	1.86E-16	-2.336 (-2.974, -1.697)	9.10E-13	0.138 (0.124, 0.152)	4.54E-80	0.693 (0.626, 0.761)	1.47E-90

Supplementary Table 5. Multivariable associations of PRSs in PRSmix+ with COPD in each cohort. Logistic regression results for PRSs in PRSmix+ predicting COPD across individual cohorts. Blue shading indicates the highest performing PRS in a cohort.

**PRS**	**COPDGene NHW**		**COPDGene AA**		**ECLIPSE**		**All of Us**		**MGBB**	
	beta (95% CI)	*adj. p-value*	*beta (95% CI)*	*adj. p-value*	*beta (95% CI)*	*adj. p-value*	*beta (95% CI)*	*adj. p-value*	*beta (95% CI)*	*adj. p-value*
PRS FEV1/FVC	0.602 (0.536, 0.668)	9.45E-71	0.400 (0.296, 0.506)	2.98E-13	0.686 (0.487, 0.891)	1.16E-10	0.186 (0.150, 0.222)	2.93E-23	0.236 (0.137, 0.336)	1.33E-05
PRS FEV1	0.506 (0.443, 0.571)	5.23E-54	0.281 (0.180, 0.383)	1.58E-07	0.513 (0.325, 0.707)	3.38E-07	0.175 (0.139, 0.211)	8.76E-21	0.183 (0.084, 0.283)	7.82E-04
PRS DLspiro	0.447 (0.385, 0.510)	1.77E-44	0.189 (0.086, 0.292)	6.27E-04	0.407 (0.220, 0.597)	4.71E-05	0.103 (0.069, 0.138)	8.43E-09	0.117 (0.018, 0.217)	3.44E-02
PRS smoking	0.113 (0.049, 0.176)	5.57E-04	0.152 (0.048, 0.257)	0.00696	0.168 (-0.025, 0.363)	0.142	0.117 (0.076, 0.158)	3.34E-08	0.074 (-0.029, 0.177)	1.82E-01
PRS IPF	0.115 (0.056, 0.175)	2.09E-04	-0.053 (-0.161, 0.055)	0.387	0.141 (-0.040, 0.325)	0.171	-0.009 (-0.057, 0.040)	0.725	0.101 (-0.010, 0.212)	9.98E-02
PRS BMI	0.177 (0.113, 0.242)	1.05E-07	-0.084 (-0.254, 0.085)	0.387	0.088 (-0.107, 0.282)	4.31E-01	0.602 (0.458, 0.746)	5.01E-16	0.255 (0.038, 0.472)	3.44E-02
PRS CRP	0.096 (0.036, 0.155)	1.66E-03	0.034 (-0.066, 0.135)	0.502	-0.056 (-0.240, 0.127)	5.50E-01	0.097 (0.062, 0.133)	9.59E-08	0.043 (-0.056, 0.142)	0.398
PRS multi	0.693 (0.626, 0.761)	1.17E-89	0.413 (0.309, 0.519)	1.08E-13	0.736 (0.537, 0.941)	7.16E-12	0.209 (0.175, 0.244)	1.23E-31	0.260 (0.161, 0.359)	1.96E-06

Supplementary Table 6. Multivariable associations of PRSs in PRSmix+ with exacerbations in each cohort. Negative binomial regression results for PRSs in PRSmix+ predicting exacerbation frequency across individual cohorts. Blue shading indicates the highest performing PRS in a cohort.

**PRS**	**COPDGene NHW**		**COPDGene AA**		**ECLIPSE**		**All of Us**		**MGBB**	
	beta (95% CI)	*adj. p-value*	*beta (95% CI)*	*adj. p-value*	*beta (95% CI)*	*adj. p-value*	*beta (95% CI)*	*adj. p-value*	*beta (95% CI)*	*adj. p-value*
PRS FEV1/FVC	0.188 (0.128, 0.248)	5.27E-11	0.142 (0.024, 0.261)	0.0327	0.087 (0.032, 0.142)	4.48E-03	0.203 (0.158, 0.248)	3.24E-18	0.482 (0.266, 0.709)	6.86E-05
PRS FEV1	0.168 (0.109, 0.227)	4.48E-09	0.123 (0.013, 0.233)	0.049	0.047 (-0.007, 0.101)	0.113	0.218 (0.173, 0.263)	1.27E-20	0.374 (0.141, 0.613)	2.50E-03
PRS DLspiro	0.154 (0.095, 0.213)	6.72E-08	0.017 (-0.095, 0.130)	0.86	0.075 (0.021, 0.129)	0.0134	0.131 (0.088, 0.174)	4.09E-09	0.163 (-0.059, 0.388)	0.176
PRS smoking	0.059 (-0.003, 0.121)	0.0513	0.007 (-0.114, 0.128)	0.905	0.044 (-0.012, 0.099)	0.138	0.145 (0.094, 0.195)	3.27E-08	0.211 (-0.019, 0.444)	1.05E-01
PRS IPF	0.014 (-0.046, 0.073)	6.21E-01	-0.145 (-0.270, -0.022)	0.0327	0.002 (-0.052, 0.056)	9.48E-01	0.008 (-0.052, 0.068)	7.93E-01	-0.091 (-0.342, 0.157)	4.74E-01
PRS BMI	0.146 (0.083, 0.208)	2.09E-06	-0.333 (-0.534, -0.132)	0.00351	0.092 (0.033, 0.151)	4.48E-03	0.654 (0.478, 0.831)	6.98E-13	1.211 (0.709, 1.736)	2.17E-05
PRS CRP	0.083 (0.023, 0.143)	3.65E-03	0.023 (-0.099, 0.144)	0.86	0.054 (-0.001, 0.108)	0.0855	0.109 (0.066, 0.153)	9.51E-07	0.355 (0.152, 0.566)	3.31E-03
PRS multi	0.229 (0.170, 0.288)	1.49E-15	0.107 (-0.005, 0.218)	0.0842	0.101 (0.047, 0.156)	1.72E-03	0.246 (0.203, 0.289)	4.52E-28	0.490 (0.276, 0.714)	6.43E-05

Supplementary Table 7. S-PrediXcan proteins significantly associated with COPD and exacerbations. List of proteins with significant associations based on S-PrediXcan using GWAS summary statistics for PRS_{multi} traits that were also significant in measuring proteomic association analysis using COPDGene SomaScan and UKBB Olink data.

FEV1/FVC									
**HGNC symbol**	**UniProt ID**	**Protein name**	**S-PrediXcan result**		**COPDGene NHW SomaScan result**		**UK Biobank Olink result**		*concordance*
			Z score	*adj. p-value*	*beta (95% CI)*	*adj. p-value*	*beta (95% CI)*	*adj. p-value*	
HGNC:14631	Q86TH1	ADAMTSL2	-2.41	9.34E-02	0.207 (0.139, 0.277)	3.01E-08	0.351 (0.230, 0.473)	1.87E-09	1
HGNC:320	Q15109	AGER (RAGE/sRAGE)	-27.1	1.75E-158	-0.158 (-0.236, -0.081)	5.41E-04	-0.315 (-0.427, -0.205)	2.83E-07	1
HGNC:329	O00468	AGRN	2.79	4.22E-02	0.078 (0.009, 0.147)	6.53E-02	0.324 (0.215, 0.435)	3.44E-09	1
HGNC:1248	P06681	C2	-7.19	5.25E-11	0.108 (0.043, 0.171)	1.25E-02	0.308 (0.176, 0.447)	2.08E-05	1
HGNC:10616	P55774	CCL18	-2.45	8.81E-02	0.212 (0.148, 0.276)	3.17E-09	0.360 (0.241, 0.481)	1.18E-09	1
HGNC:10627	P10147	CCL3	-2.45	8.81E-02	0.129 (0.059, 0.201)	4.06E-04	0.386 (0.256, 0.522)	2.64E-12	1
HGNC:1628	P08571	CD14	2.59	6.63E-02	0.100 (0.035, 0.165)	1.52E-02	0.354 (0.241, 0.469)	3.90E-09	1
HGNC:1037	P00751	CFB	-2.38	9.78E-02	0.082 (0.013, 0.152)	7.62E-02	0.479 (0.344, 0.617)	6.03E-11	1
HGNC:2195	P39060	COL18A1	-4.74	5.81E-05	0.141 (0.072, 0.211)	6.73E-04	0.480 (0.365, 0.597)	1.06E-15	1
HGNC:2213	P12111	COL6A3	-4.98	2.03E-05	-0.078 (-0.141, -0.014)	5.66E-02	0.322 (0.220, 0.428)	2.36E-09	0
HGNC:3061	P23919	DTYMK	-2.83	3.89E-02	-0.104 (-0.200, -0.008)	8.66E-02	0.142 (0.015, 0.271)	5.39E-02	0
HGNC:3218	Q12805	EFEMP1	8.52	2.04E-15	0.200 (0.129, 0.273)	7.86E-07	0.298 (0.186, 0.413)	1.16E-06	1
HGNC:3386	P29317	EPHA2	3.08	2.07E-02	0.144 (0.070, 0.219)	1.11E-03	0.259 (0.151, 0.369)	5.03E-06	1
HGNC:18506	Q96P31	FCRL3	2.68	5.50E-02	0.104 (0.026, 0.184)	1.36E-02	-0.139 (-0.260, -0.020)	5.11E-02	0
HGNC:3695	Q08830	FGL1	6.97	2.31E-10	0.132 (0.065, 0.199)	1.11E-03	0.428 (0.309, 0.550)	7.59E-11	1
HGNC:4114	P22466	GAL	3.33	1.04E-02	-0.076 (-0.144, -0.006)	8.66E-02	-0.208 (-0.323, -0.093)	1.35E-03	1
HGNC:4243	P56159	GFRA1	2.78	4.33E-02	0.081 (0.005, 0.158)	6.27E-02	0.367 (0.257, 0.478)	3.22E-10	1
HGNC:4367	P17900	GM2A	-6.27	1.90E-08	0.196 (0.123, 0.271)	3.31E-07	0.294 (0.175, 0.419)	1.78E-08	1
HGNC:4453	P78333	GPC5	-3.4	8.86E-03	-0.159 (-0.231, -0.088)	3.28E-04	-0.221 (-0.337, -0.107)	5.19E-04	1
HGNC:5344	P05362	ICAM1	2.76	4.48E-02	0.123 (0.055, 0.191)	1.84E-03	0.504 (0.392, 0.619)	3.89E-19	1

HGNC:5988	Q13478	IL18R1	3.33	1.04E-02	0.089 (0.018, 0.160)	5.16E-02	0.268 (0.153, 0.386)	1.51E-05	1
HGNC:5998	Q01638	IL1RL1	6.89	3.62E-10	0.179 (0.112, 0.247)	5.30E-06	0.195 (0.078, 0.313)	3.28E-03	1
HGNC:6168	Q06033	ITIH3	2.59	6.63E-02	0.129 (0.063, 0.194)	1.11E-03	0.357 (0.235, 0.480)	7.11E-08	1
HGNC:6563	P17931	LGALS3	-3.36	9.71E-03	0.114 (0.045, 0.183)	8.34E-03	0.322 (0.207, 0.440)	1.25E-07	1
HGNC:16309	A6NI73	LILRA5	2.82	3.96E-02	0.163 (0.092, 0.234)	4.92E-05	0.553 (0.434, 0.676)	1.46E-18	1
HGNC:6606	Q8N423	LILRB2	3.22	1.44E-02	0.125 (0.060, 0.188)	1.19E-03	0.188 (0.071, 0.306)	6.82E-03	1
HGNC:6710	P09960	LTA4H	3.26	1.29E-02	0.085 (0.016, 0.155)	5.48E-02	0.236 (0.127, 0.345)	4.63E-05	1
HGNC:7036	Q08431	MFGE8	-3.39	9.05E-03	0.095 (0.016, 0.174)	5.77E-02	0.234 (0.122, 0.347)	1.17E-04	1
HGNC:19869	Q96GP6	SCARF2	9.42	6.40E-19	0.082 (0.011, 0.152)	7.94E-02	0.153 (0.032, 0.274)	2.74E-02	1
HGNC:1228	P05155	SERPING1	6.44	6.82E-09	-0.076 (-0.147, -0.006)	8.66E-02	0.315 (0.195, 0.438)	2.35E-06	0
**FEV1**									
**HGNC symbol**	**UniProt ID**	**Protein name**	**MetaXcan result**		**COPDGene NHW SomaScan result**		**UK Biobank Olink result**		*concordance*
			Z score	*adj. p-value*	*beta (95% CI)*	*adj. p-value*	*beta (95% CI)*	*adj. p-value*	
HGNC:259	P35318	ADM	2.48	8.36E-02	-0.080 (-0.152, -0.011)	8.60E-02	0.676 (0.550, 0.806)	5.12E-24	0
HGNC:320	Q15109	AGER (RAGE/sRAGE)	-4.57	1.90E-04	-0.158 (-0.236, -0.081)	4.07E-04	-0.315 (-0.427, -0.205)	3.71E-07	1
HGNC:489	O95841	ANGPTL1	2.84	4.14E-02	0.114 (0.051, 0.178)	3.08E-03	0.183 (0.069, 0.299)	6.23E-03	1
HGNC:21265	Q9NZP8	C1RL	-3.26	1.53E-02	0.143 (0.080, 0.206)	2.36E-04	0.386 (0.256, 0.520)	1.15E-07	1
HGNC:1248	P06681	C2	-8.86	2.65E-16	0.108 (0.043, 0.171)	1.03E-02	0.308 (0.176, 0.447)	2.35E-05	1
HGNC:1318	P01024	C3	3.39	1.09E-02	0.083 (0.011, 0.156)	5.53E-02	0.160 (0.024, 0.298)	2.56E-02	1
HGNC:10624	O15444	CCL25	2.72	5.20E-02	0.079 (0.010, 0.149)	6.02E-02	0.261 (0.149, 0.375)	4.32E-05	1
HGNC:1628	P08571	CD14	4.35	4.23E-04	0.100 (0.035, 0.165)	1.27E-02	0.354 (0.241, 0.469)	5.76E-09	1
HGNC:6953	P15529	CD46	4.18	7.43E-04	0.087 (0.019, 0.156)	4.01E-02	0.164 (0.053, 0.276)	8.82E-03	1
HGNC:1037	P00751	CFB	-3.1	2.16E-02	0.082 (0.013, 0.152)	5.92E-02	0.479 (0.344, 0.617)	5.20E-11	1
HGNC:2213	P12111	COL6A3	-3.33	1.30E-02	-0.078 (-0.141, -0.014)	4.41E-02	0.322 (0.220, 0.428)	3.37E-09	0
HGNC:3061	P23919	DTYMK	-2.55	7.06E-02	-0.104 (-0.200, -0.008)	6.77E-02	0.142 (0.015, 0.271)	4.85E-02	0
HGNC:3218	Q12805	EFEMP1	-7.17	1.05E-10	0.200 (0.129, 0.273)	1.88E-06	0.298 (0.186, 0.413)	1.36E-06	1
HGNC:3395	P54760	EPHB4	2.44	9.18E-02	0.087 (0.015, 0.160)	5.25E-02	0.151 (0.052, 0.252)	1.22E-02	1
HGNC:4114	P22466	GAL	5.25	7.35E-06	-0.076 (-0.144, -0.006)	6.79E-02	-0.208 (-0.323, -0.093)	1.43E-03	1
HGNC:4367	P17900	GM2A	-6.07	9.49E-08	0.196 (0.123, 0.271)	1.26E-06	0.294 (0.175, 0.419)	2.38E-08	1

HGNC:4453	P78333	GPC5	-3.28	1.48E-02	-0.159 (-0.231, -0.088)	2.46E-04	-0.221 (-0.337, -0.107)	5.55E-04	1
HGNC:4462	Q14956	GPNMB	-2.95	3.09E-02	0.067 (0.005, 0.129)	9.10E-02	0.158 (0.040, 0.277)	1.87E-02	1
HGNC:5344	P05362	ICAM1	2.9	3.53E-02	0.123 (0.055, 0.191)	1.62E-03	0.504 (0.392, 0.619)	2.56E-19	1
HGNC:5476	Q16270	IGFBP7	-2.48	8.36E-02	0.152 (0.086, 0.219)	1.00E-04	0.266 (0.159, 0.375)	1.59E-06	1
HGNC:5998	Q01638	IL1RL1	3.83	2.70E-03	0.179 (0.112, 0.247)	5.90E-06	0.195 (0.078, 0.313)	3.40E-03	1
HGNC:6000	P18510	IL1RN	3.78	3.02E-03	0.170 (0.101, 0.240)	4.22E-06	0.320 (0.203, 0.438)	1.67E-08	1
HGNC:16936	Q86VZ4	LRP11	-3.01	2.71E-02	0.140 (0.071, 0.210)	4.07E-04	0.153 (0.046, 0.261)	1.87E-02	1
HGNC:9104	O15031	PLXNB2	-5.13	1.30E-05	0.179 (0.110, 0.248)	5.65E-06	0.222 (0.110, 0.337)	4.26E-04	1
HGNC:16753	P30041	PRDX6	2.69	5.55E-02	-0.176 (-0.267, -0.085)	7.39E-04	-0.163 (-0.282, -0.046)	1.64E-02	1
HGNC:9618	Q13308	PTK7	-2.97	2.90E-02	0.165 (0.099, 0.233)	2.41E-05	0.194 (0.082, 0.305)	2.78E-03	1
HGNC:19869	Q96GP6	SCARF2	6.28	2.94E-08	0.082 (0.011, 0.152)	6.25E-02	0.153 (0.032, 0.274)	2.45E-02	1
HGNC:8941	P01009	SERPINA1	-2.78	4.62E-02	0.072 (0.006, 0.139)	6.76E-02	0.332 (0.199, 0.467)	8.57E-07	1
HGNC:8824	P36955	SERPINF1	-3.59	6.07E-03	0.133 (0.067, 0.199)	5.00E-04	0.146 (0.032, 0.262)	4.49E-02	1
HGNC:1228	P05155	SERPING1	3.94	1.82E-03	-0.076 (-0.147, -0.006)	6.77E-02	0.315 (0.195, 0.438)	2.50E-06	0
HGNC:11120	P17405	SMPD1	-3.85	2.50E-03	0.090 (0.021, 0.159)	3.07E-02	0.364 (0.264, 0.466)	5.30E-12	1
HGNC:11252	Q9HCB6	SPON1	-2.72	5.20E-02	0.137 (0.065, 0.210)	7.79E-04	0.379 (0.266, 0.494)	6.84E-11	1
HGNC:11823	Q99727	TIMP4	4.17	7.81E-04	0.165 (0.098, 0.233)	1.63E-05	0.376 (0.272, 0.483)	2.71E-12	1
HGNC:11927	O43508	TNFSF12	3.27	1.50E-02	-0.142 (-0.201, -0.080)	5.00E-04	-0.263 (-0.380, -0.147)	2.62E-05	1
**DL spirometry**									
**HGNC symbol**	**UniProt ID**	**Protein name**	**MetaXcan result**		**COPDGene NHW SomaScan result**		**UK Biobank Olink result**		*concordance*
			Z score	*adj. p-value*	*beta (95% CI)*	*adj. p-value*	*beta (95% CI)*	*adj. p-value*	
HGNC:320	Q15109	AGER (RAGE/sRAGE)	16.5	1.22E-58	-0.158 (-0.236, -0.081)	3.33E-04	-0.315 (-0.427, -0.205)	3.63E-07	1
HGNC:1248	P06681	C2	9.31	1.16E-18	0.108 (0.043, 0.171)	8.07E-03	0.308 (0.176, 0.447)	2.16E-05	1
HGNC:4367	P17900	GM2A	2.91	3.24E-02	0.196 (0.123, 0.271)	3.48E-07	0.294 (0.175, 0.419)	9.05E-08	1
HGNC:4453	P78333	GPC5	5.79	1.83E-07	-0.159 (-0.231, -0.088)	1.90E-04	-0.221 (-0.337, -0.107)	4.79E-04	1
HGNC:5988	Q13478	IL18R1	-2.82	4.15E-02	0.089 (0.018, 0.160)	3.06E-02	0.268 (0.153, 0.386)	1.67E-05	1
HGNC:5998	Q01638	IL1RL1	-6.94	1.85E-10	0.179 (0.112, 0.247)	4.88E-06	0.195 (0.078, 0.313)	2.99E-03	1
HGNC:6168	Q06033	ITIH3	-3.83	1.66E-03	0.129 (0.063, 0.194)	6.29E-04	0.357 (0.235, 0.480)	1.04E-07	1
HGNC:16820	Q14162	SCARF1	3.61	3.56E-03	0.093 (0.007, 0.180)	5.72E-02	0.235 (0.126, 0.346)	7.15E-05	1
HGNC:19869	Q96GP6	SCARF2	-9.08	8.50E-18	0.082 (0.011, 0.152)	5.55E-02	0.153 (0.032, 0.274)	2.56E-02	1
HGNC:1228	P05155	SERPING1	-2.59	7.50E-02	-0.076 (-0.147, -0.006)	5.72E-02	0.315 (0.195, 0.438)	2.83E-06	0
HGNC:11927	O43508	TNFSF12	-3.06	2.21E-02	-0.142 (-0.201, -0.080)	3.77E-04	-0.263 (-0.380, -0.147)	2.22E-05	1
**Smoking**									
		**Protein name**	**MetaXcan result**		**COPDGene NHW SomaScan result**		**UK Biobank Olink result**		*concordance*

HGNC symbol	UniProt ID		Z score	adj. p-value	beta (95% CI)	adj. p-value	beta (95% CI)	adj. p-value	
HGNC:16936	Q86VZ4	LRP11	-3.95	2.49E-02	0.140 (0.071, 0.210)	1.16E-04	0.153 (0.046, 0.261)	1.88E-02	1
**IPF**									
**HGNC symbol**	**UniProt ID**	**Protein name**	**MetaXcan result**		**COPDGene NHW SomaScan result**		**UK Biobank Olink result**		*concordance*
			Z score	*adj. p-value*	*beta (95% CI)*	*adj. p-value*	*beta (95% CI)*	*adj. p-value*	
**BMI**									
**HGNC symbol**	**UniProt ID**	**Protein name**	**MetaXcan result**		**COPDGene NHW SomaScan result**		**UK Biobank Olink result**		*concordance*
			Z score	*adj. p-value*	*beta (95% CI)*	*adj. p-value*	*beta (95% CI)*	*adj. p-value*	
HGNC:320	Q15109	AGER (RAGE/sRAGE)	-2.79	3.50E-02	-0.158 (-0.236, -0.081)	6.13E-04	-0.315 (-0.427, -0.205)	3.25E-07	1
HGNC:1248	P06681	C2	5.92	1.84E-07	0.108 (0.043, 0.171)	1.28E-02	0.308 (0.176, 0.447)	2.06E-05	1
HGNC:1380	P23280	CA6	2.53	5.99E-02	-0.271 (-0.341, -0.200)	2.98E-13	-0.460 (-0.578, -0.344)	1.18E-13	1
HGNC:1438	P10092	CALCB	5.13	9.01E-06	0.127 (0.051, 0.206)	2.19E-03	0.280 (0.159, 0.403)	2.39E-06	1
HGNC:10632	P13501	CCL5	2.69	4.38E-02	-0.125 (-0.195, -0.056)	3.09E-03	0.182 (0.061, 0.305)	5.96E-03	0
HGNC:18219	Q9HCU0	CD248	4.67	6.67E-05	-0.081 (-0.149, -0.013)	7.32E-02	-0.180 (-0.303, -0.060)	1.04E-02	1
HGNC:1037	P00751	CFB	-2.68	4.42E-02	0.082 (0.013, 0.152)	7.96E-02	0.479 (0.344, 0.617)	3.50E-11	1
HGNC:11891	P05452	CLEC3B	2.74	3.95E-02	-0.154 (-0.222, -0.086)	1.35E-04	-0.311 (-0.445, -0.181)	1.53E-05	1
HGNC:2195	P39060	COL18A1	-2.61	5.15E-02	0.141 (0.072, 0.211)	7.31E-04	0.480 (0.365, 0.597)	9.42E-16	1
HGNC:2336	P20023	CR2	-2.98	2.15E-02	-0.091 (-0.153, -0.028)	2.75E-02	-0.138 (-0.247, -0.029)	3.52E-02	1
HGNC:3218	Q12805	EFEMP1	4.81	3.89E-05	0.200 (0.129, 0.273)	1.78E-06	0.298 (0.186, 0.413)	1.33E-06	1
HGNC:3395	P54760	EPHB4	-2.84	3.11E-02	0.087 (0.015, 0.160)	7.32E-02	0.151 (0.052, 0.252)	1.04E-02	1
HGNC:4114	P22466	GAL	5.99	1.21E-07	-0.076 (-0.144, -0.006)	9.29E-02	-0.208 (-0.323, -0.093)	1.20E-03	1
HGNC:30142	Q99988	GDF15	4.57	1.04E-04	0.295 (0.221, 0.370)	2.98E-13	0.736 (0.626, 0.848)	6.51E-50	1
HGNC:5344	P05362	ICAM1	3.74	2.37E-03	0.123 (0.055, 0.191)	2.27E-03	0.504 (0.392, 0.619)	2.42E-19	1
HGNC:5348	Q9UMF0	ICAM5	-3.36	7.47E-03	0.079 (0.008, 0.151)	9.73E-02	0.391 (0.280, 0.504)	2.39E-10	1
HGNC:5391	P35475	IDUA	-3.26	1.02E-02	0.090 (0.019, 0.161)	4.89E-02	0.303 (0.184, 0.424)	3.90E-06	1
HGNC:5471	P18065	IGFBP2	-2.37	8.08E-02	0.094 (0.024, 0.164)	3.17E-02	0.316 (0.196, 0.438)	2.01E-06	1
HGNC:5998	Q01638	IL1RL1	2.63	4.91E-02	0.179 (0.112, 0.247)	8.39E-06	0.195 (0.078, 0.313)	2.89E-03	1
HGNC:6168	Q06033	ITIH3	7.8	1.11E-12	0.129 (0.063, 0.194)	1.33E-03	0.357 (0.235, 0.480)	9.26E-08	1
HGNC:6563	P17931	LGALS3	4.19	4.80E-04	0.114 (0.045, 0.183)	9.26E-03	0.322 (0.207, 0.440)	1.55E-07	1
HGNC:7218	P05164	MPO	2.73	4.09E-02	0.179 (0.110, 0.249)	6.85E-06	0.178 (0.065, 0.291)	2.93E-03	1
HGNC:7650	P41271	NBL1	4.87	3.10E-05	0.166 (0.092, 0.242)	1.01E-04	0.265 (0.157, 0.374)	5.12E-06	1
HGNC:7656	P13591	NCAM1	3.91	1.31E-03	-0.158 (-0.224, -0.092)	8.99E-05	-0.210 (-0.327, -0.095)	9.90E-04	1
HGNC:9618	Q13308	PTK7	2.69	4.40E-02	0.165 (0.099, 0.233)	3.61E-05	0.194 (0.082, 0.305)	2.34E-03	1

HGNC:9868	Q99969	RARRES2	-3.32	8.54E-03	0.105 (0.037, 0.173)	1.58E-02	0.512 (0.390, 0.636)	4.15E-16	1
HGNC:19869	Q96GP6	SCARF2	2.55	5.70E-02	0.082 (0.011, 0.152)	8.54E-02	0.153 (0.032, 0.274)	2.23E-02	1
HGNC:10732	Q92854	SEMA4D	-3.52	4.84E-03	0.109 (0.040, 0.178)	1.26E-02	0.120 (0.003, 0.239)	8.01E-02	1
HGNC:1228	P05155	SERPING1	-3.04	1.86E-02	-0.076 (-0.147, -0.006)	9.29E-02	0.315 (0.195, 0.438)	2.55E-06	0
HGNC:11823	Q99727	TIMP4	-3.74	2.37E-03	0.165 (0.098, 0.233)	2.32E-05	0.376 (0.272, 0.483)	1.55E-12	1
HGNC:11927	O43508	TNFSF12	3.21	1.18E-02	-0.142 (-0.201, -0.080)	7.31E-04	-0.263 (-0.380, -0.147)	2.32E-05	1
HGNC:17761	Q9NZC2	TREM2	4.19	4.80E-04	0.080 (0.009, 0.150)	9.63E-02	0.546 (0.437, 0.659)	3.29E-22	1
HGNC:21073	Q8NBS9	TXNDC5	-2.82	3.21E-02	0.159 (0.078, 0.241)	7.31E-04	0.211 (0.095, 0.328)	8.17E-04	1

CRP

HGNC symbol	UniProt ID	Protein name	MetaXcan result		COPDGene NHW SomaScan result		UK Biobank Olink result		*concordance*
			Z score	*adj. p-value*	*beta (95% CI)*	*adj. p-value*	*beta (95% CI)*	*adj. p-value*	
HGNC:320	Q15109	AGER (RAGE/sRAGE)	-7.23	4.31E-11	-0.158 (-0.236, -0.081)	3.74E-04	-0.315 (-0.427, -0.205)	3.14E-07	1
HGNC:329	O00468	AGRN	-2.79	5.16E-02	0.078 (0.009, 0.147)	5.87E-02	0.324 (0.215, 0.435)	4.18E-09	1
HGNC:491	Q9Y5C1	ANGPTL3	4.84	5.93E-05	0.074 (0.006, 0.142)	8.93E-02	0.246 (0.132, 0.362)	1.22E-04	1
HGNC:18219	Q9HCU0	CD248	3.64	5.53E-03	-0.081 (-0.149, -0.013)	6.07E-02	-0.180 (-0.303, -0.060)	1.12E-02	1
HGNC:19320	Q08708	CD300C	2.61	8.01E-02	0.177 (0.108, 0.246)	5.18E-06	0.181 (0.066, 0.297)	5.57E-03	1
HGNC:11891	P05452	CLEC3B	2.91	4.12E-02	-0.154 (-0.222, -0.086)	1.12E-04	-0.311 (-0.445, -0.181)	1.56E-05	1
HGNC:2300	Q96IY4	CPB2	-3.73	4.08E-03	-0.141 (-0.207, -0.075)	2.93E-04	0.163 (0.035, 0.293)	2.65E-02	0
HGNC:3218	Q12805	EFEMP1	-2.55	9.28E-02	0.200 (0.129, 0.273)	2.72E-06	0.298 (0.186, 0.413)	1.20E-06	1
HGNC:3386	P29317	EPHA2	-2.85	4.51E-02	0.144 (0.070, 0.219)	9.02E-04	0.259 (0.151, 0.369)	5.28E-06	1
HGNC:4696	P08236	GUSB	3.48	8.66E-03	0.123 (0.052, 0.196)	3.16E-03	0.249 (0.132, 0.368)	6.98E-05	1
HGNC:5391	P35475	IDUA	2.89	4.22E-02	0.090 (0.019, 0.161)	4.06E-02	0.303 (0.184, 0.424)	4.07E-06	1
HGNC:5987	O95998	IL18BP	-2.69	6.73E-02	0.122 (0.047, 0.197)	7.54E-03	0.339 (0.230, 0.450)	7.28E-09	1
HGNC:5988	Q13478	IL18R1	5.3	6.32E-06	0.089 (0.018, 0.160)	4.46E-02	0.268 (0.153, 0.386)	1.56E-05	1
HGNC:5998	Q01638	IL1RL1	3.49	8.38E-03	0.179 (0.112, 0.247)	5.18E-06	0.195 (0.078, 0.313)	2.98E-03	1
HGNC:6000	P18510	IL1RN	-20.1	3.44E-87	0.170 (0.101, 0.240)	4.22E-06	0.320 (0.203, 0.438)	1.34E-08	1
HGNC:6067	P09529	INHBB	5.64	1.01E-06	0.079 (0.007, 0.152)	7.90E-02	0.315 (0.204, 0.428)	2.37E-07	1
HGNC:16936	Q86VZ4	LRP11	-3.59	6.39E-03	0.140 (0.071, 0.210)	3.74E-04	0.153 (0.046, 0.261)	1.73E-02	1
HGNC:2465	O14594	NCAN	-8.16	3.74E-14	-0.123 (-0.195, -0.051)	4.27E-03	-0.249 (-0.358, -0.141)	5.06E-05	1
HGNC:9618	Q13308	PTK7	2.96	3.76E-02	0.165 (0.099, 0.233)	2.24E-05	0.194 (0.082, 0.305)	2.48E-03	1
HGNC:9630	P21246	PTN	-2.95	3.83E-02	0.093 (0.007, 0.180)	9.70E-02	0.221 (0.111, 0.333)	2.21E-04	1
HGNC:9868	Q99969	RARRES2	-4.09	1.14E-03	0.105 (0.037, 0.173)	1.20E-02	0.512 (0.390, 0.636)	3.93E-16	1
HGNC:19869	Q96GP6	SCARF2	2.65	7.39E-02	0.082 (0.011, 0.152)	7.45E-02	0.153 (0.032, 0.274)	2.29E-02	1
HGNC:8941	P01009	SERPINA1	3	3.29E-02	0.072 (0.006, 0.139)	7.90E-02	0.332 (0.199, 0.467)	7.23E-07	1

HGNC:11559	P37837	TALDO1	-2.54	9.46E-02	0.159 (0.084, 0.235)	3.54E-04	0.199 (0.069, 0.331)	2.98E-03	1
HGNC:11909	O00300	TNFRSF11B	3.25	1.61E-02	0.117 (0.045, 0.190)	6.77E-03	0.429 (0.317, 0.544)	9.37E-14	1
HGNC:11916	P19438	TNFRSF1A	7.82	5.45E-13	0.162 (0.087, 0.237)	2.93E-04	0.340 (0.233, 0.449)	3.88E-10	1
HGNC:11929	Q9Y275	TNFSF13B	-2.91	4.12E-02	0.105 (0.031, 0.182)	8.49E-03	0.449 (0.347, 0.554)	1.69E-18	1

Supplementary Table 8. Clinical trial information from OpenTargets for drugs targeting S-PrediXcan proteins. Summary of drugs targeting proteins identified via S-PrediXcan analysis. Includes drug names, indications, and clinical trial phase information.

protein name	*drug name*	*disease name*	*clinical trial phase*	*source*
AGER (RAGE/sRAGE)	AZELIRAGON	Alzheimer disease	3	https://clinicaltrials.gov/study/NCT02080364,https://clinicaltrials.gov/study/NCT02916056
AGER (RAGE/sRAGE)	AZELIRAGON	COVID-19	2	https://clinicaltrials.gov/study/NCT05815485
AGER (RAGE/sRAGE)	AZELIRAGON	diabetic nephropathy	2	https://clinicaltrials.gov/study/NCT00287183
AGER (RAGE/sRAGE)	AZELIRAGON	glioblastoma multiforme	2	https://clinicaltrials.gov/study/NCT05986851
ANGPTL3	EVINACUMAB	Hypercholesterolemia	4	https://www.ema.europa.eu/en/medicines/human/EPAR/evkeeza
ANGPTL3	EVINACUMAB	Hypertriglyceridemia	2	https://clinicaltrials.gov/study/NCT03452228
ANGPTL3	EVINACUMAB	cardiovascular disease	4	https://www.whocc.no/atc_ddd_index/?code=C10AX17
ANGPTL3	EVINACUMAB	familial hypercholesterolemia	4	https://dailymed.nlm.nih.gov/dailymed/drugInfo.cfm?setid=73412138-6d8f-4ea6-bb72-a740190470ff
ANGPTL3	EVINACUMAB	homozygous familial hypercholesterolemia	4	https://www.accessdata.fda.gov/drugsatfda_docs/label/2021/761181s000lbl.pdf
ANGPTL3	EVINACUMAB	metabolic disease	1	https://clinicaltrials.gov/study/NCT02107872
ANGPTL3	VUPANORSEN	familial partial lipodystrophy	2	https://clinicaltrials.gov/study/NCT03514420
ANGPTL3	VUPANORSEN	homozygous familial hypercholesterolemia	2	https://clinicaltrials.gov/study/NCT03455777

C3	AL-78898A	age-related macular degeneration	2	https://clinicaltrials.gov/study/NCT01157065
C3	AL-78898A	atrophic macular degeneration	2	https://clinicaltrials.gov/study/NCT01603043
C3	AMY-101	gingivitis	1	https://clinicaltrials.gov/study/NCT03694444
C3	PEGCETACOPLAN	age-related macular degeneration	1	https://clinicaltrials.gov/study/NCT02461771
C3	PEGCETACOPLAN	amyotrophic lateral sclerosis	2	https://clinicaltrials.gov/study/NCT04579666
C3	PEGCETACOPLAN	atrophic macular degeneration	3	https://clinicaltrials.gov/study/NCT04770545
C3	PEGCETACOPLAN	cold agglutinin disease	3	https://clinicaltrials.gov/study/NCT05096403
C3	PEGCETACOPLAN	dense deposit disease	2	https://clinicaltrials.gov/study/NCT04572854
C3	PEGCETACOPLAN	immune system disease	4	https://www.whocc.no/atc_ddd_index/?code=L04AA54
C3	PEGCETACOPLAN	paroxysmal nocturnal hemoglobinuria	4	https://www.accessdata.fda.gov/drugsatfda_docs/label/2021/215014s000lbl.pdf,https://www.ema.europa.eu/en/medicines/human/EPAR/aspaveli
C3	PEGCETACOPLAN	thrombotic microangiopathy	2	https://clinicaltrials.gov/study/NCT05148299
CA6	SULTHIAME	epilepsy	4	https://www.whocc.no/atc_ddd_index/?code=N03AX03
CA6	SULTHIAME	obstructive sleep apnea	2	https://clinicaltrials.gov/study/NCT05236842
CALCB	EPTINEZUMAB	diabetic polyneuropathy	2	https://clinicaltrials.gov/study/NCT05937152
CALCB	EPTINEZUMAB	migraine disorder	4	https://clinicaltrials.gov/study/NCT05284019
CALCB	EPTINEZUMAB	pain	3	https://clinicaltrials.gov/study/NCT04688775,https://clinicaltrials.gov/study/NCT05064397
CALCB	FREMANEZUMAB	fibromyalgia	2	https://clinicaltrials.gov/study/NCT03965091
CALCB	FREMANEZUMAB	interstitial cystitis	2	https://clinicaltrials.gov/study/NCT04447729
CALCB	FREMANEZUMAB	migraine disorder	4	https://dailymed.nlm.nih.gov/dailymed/drugInfo.cfm?setid=98e344ea-5916-4947-b6f2-4a76ccc04b6b,https://www.ema.europa.eu/en/medicines/human/EPAR/ajovy,https://www.accessdata.fda.gov/drugsatfda_docs/label/2018/761089s000lbl.pdf,https://www.whocc.no/atc_ddd_index/?code=N02CD03
CALCB	FREMANEZUMAB	pain	3	https://clinicaltrials.gov/study/NCT02945046,https://clinicaltrials.gov/study/NCT02964338,https://clinicaltrials.gov/study/NCT03107052
CALCB	GALCANEZUMAB	migraine disorder	4	https://clinicaltrials.gov/study/NCT05127486,https://clinicaltrials.gov/study/NCT04294147
CALCB	GALCANEZUMAB	osteoarthritis, knee	2	https://clinicaltrials.gov/study/NCT02192190
CALCB	GALCANEZUMAB	pain	3	https://clinicaltrials.gov/study/NCT02797951,https://clinicaltrials.gov/study/NCT02397473

CD14	IC14	COVID-19	2	https://clinicaltrials.gov/study/NCT04488081
CD14	IC14	acute respiratory distress syndrome	2	https://clinicaltrials.gov/study/NCT03017547
CD14	IC14	adult acute respiratory distress syndrome	2	https://clinicaltrials.gov/study/NCT00233207
CD14	IC14	amyotrophic lateral sclerosis	2	https://clinicaltrials.gov/study/NCT03508453,https://clinicaltrials.gov/study/NCT03474263
CD14	IC14	dengue disease	2	https://clinicaltrials.gov/study/NCT03875560
CD14	IC14	motor neuron disease	2	https://clinicaltrials.gov/study/NCT03508453
CD14	VB-201	COVID-19	2	https://clinicaltrials.gov/study/NCT04733833
CD14	VB-201	psoriasis	2	https://clinicaltrials.gov/study/NCT01001468,https://clinicaltrials.gov/study/NCT01837420
CD14	VB-201	ulcerative colitis	2	https://clinicaltrials.gov/study/NCT01839214
CD248	ONTUXIZUMAB	metastatic colorectal cancer	2	https://clinicaltrials.gov/study/NCT01507545
CD248	ONTUXIZUMAB	metastatic melanoma	2	https://clinicaltrials.gov/study/NCT01335009
CD248	ONTUXIZUMAB	neoplasm	1	https://clinicaltrials.gov/study/NCT00847054,https://clinicaltrials.gov/study/NCT01773434
CD248	ONTUXIZUMAB	soft tissue sarcoma	2	https://clinicaltrials.gov/study/NCT01574716
CFB	IPTACOPAN	IGA glomerulonephritis	3	https://clinicaltrials.gov/study/NCT04557462
CFB	IPTACOPAN	age-related macular degeneration	2	https://clinicaltrials.gov/study/NCT05230537
CFB	IPTACOPAN	atypical hemolytic-uremic syndrome	3	https://clinicaltrials.gov/study/NCT05935215,https://clinicaltrials.gov/study/NCT04889430
CFB	IPTACOPAN	glomerulonephritis	2	https://clinicaltrials.gov/study/NCT03832114
CFB	IPTACOPAN	liver disease	1	https://clinicaltrials.gov/study/NCT05078580
CFB	IPTACOPAN	lupus nephritis	2	https://clinicaltrials.gov/study/NCT05268289
CFB	IPTACOPAN	membranous glomerulonephritis	2	https://clinicaltrials.gov/study/NCT04154787
CFB	IPTACOPAN	non-immunoglobulin-mediated membranoproliferative glomerulonephritis	3	https://clinicaltrials.gov/study/NCT03955445
CFB	IPTACOPAN	paroxysmal nocturnal hemoglobinuria	3	https://clinicaltrials.gov/study/NCT04558918,https://clinicaltrials.gov/study/NCT04820530
CFB	IPTACOPAN	primary membranoproliferative glomerulonephritis	3	https://clinicaltrials.gov/study/NCT05755386
COL18A1	COLLAGENASE CLOSTRIDIUM HISTOLYTICUM	Abnormality of connective tissue	4	https://dailymed.nlm.nih.gov/dailymed/drugInfo.cfm?setid=805cecd0-fd1f-11dd-87af-0800200c9a66

COL18A1	COLLAGENASE CLOSTRIDIUM HISTOLYTICUM	Dupuytren Contracture	4	https://clinicaltrials.gov/study/NCT02476461,https://clinicaltrials.gov/study/NCT03000114
COL18A1	COLLAGENASE CLOSTRIDIUM HISTOLYTICUM	Edema	3	https://clinicaltrials.gov/study/NCT03526549
COL18A1	COLLAGENASE CLOSTRIDIUM HISTOLYTICUM	Palmar Fibromatosis	4	https://www.ema.europa.eu/en/medicines/human/EPAR/xiapex
COL18A1	COLLAGENASE CLOSTRIDIUM HISTOLYTICUM	Penile Fibromatosis	4	https://dailymed.nlm.nih.gov/dailymed/drugInfo.cfm?setid=805cecd0-fd1f-11dd-87af-0800200c9a66
COL18A1	COLLAGENASE CLOSTRIDIUM HISTOLYTICUM	Peyronie disease	3	https://clinicaltrials.gov/study/NCT01685437,https://clinicaltrials.gov/study/NCT01243411,https://clinicaltrials.gov/study/NCT02267460,https://clinicaltrials.gov/study/NCT01221597,https://clinicaltrials.gov/study/NCT01221623
COL18A1	COLLAGENASE CLOSTRIDIUM HISTOLYTICUM	Skin ulcer	4	https://www.whocc.no/atc_ddd_index/?code=D03BA02,https://www.whocc.no/atc_ddd_index/?code=D03BA52
COL18A1	COLLAGENASE CLOSTRIDIUM HISTOLYTICUM	Tendinopathy	1	https://clinicaltrials.gov/study/NCT00261209
COL18A1	COLLAGENASE CLOSTRIDIUM HISTOLYTICUM	Urethral stricture	2	https://clinicaltrials.gov/study/NCT02948842
COL18A1	COLLAGENASE CLOSTRIDIUM HISTOLYTICUM	contracture	2	https://clinicaltrials.gov/study/NCT01237964
COL18A1	COLLAGENASE CLOSTRIDIUM HISTOLYTICUM	decubitus ulcer	3	https://clinicaltrials.gov/study/NCT02004626
COL18A1	COLLAGENASE CLOSTRIDIUM HISTOLYTICUM	diabetic foot	1	https://clinicaltrials.gov/study/NCT02131961
COL18A1	COLLAGENASE CLOSTRIDIUM HISTOLYTICUM	frozen shoulder	2	https://clinicaltrials.gov/study/NCT01483963,https://clinicaltrials.gov/study/NCT02006719
COL18A1	COLLAGENASE CLOSTRIDIUM HISTOLYTICUM	lipoma	2	https://clinicaltrials.gov/study/NCT02249052,https://clinicaltrials.gov/study/NCT01613313

COL18A1	COLLAGENASE CLOSTRIDIUM HISTOLYTICUM	ulcer disease	4	https://dailymed.nlm.nih.gov/dailymed/drugInfo.cfm?setid=6b6fbfc6-98fa-46aa-88ef-ab00fbb08ffd
COL18A1	OCRIPLASMIN	Abnormal retinal morphology	4	https://www.ema.europa.eu/en/medicines/human/EPAR/jetrea
COL18A1	OCRIPLASMIN	deep vein thrombosis	2	https://clinicaltrials.gov/study/NCT00428129
COL18A1	OCRIPLASMIN	diabetic macular edema	2	https://clinicaltrials.gov/study/NCT00412451
COL18A1	OCRIPLASMIN	eye disease	4	https://www.whocc.no/atc_ddd_index/?code=S01XA22
COL18A1	OCRIPLASMIN	macular degeneration	2	https://clinicaltrials.gov/study/NCT00996684
COL18A1	OCRIPLASMIN	macular holes	3	https://clinicaltrials.gov/study/NCT01429441
COL18A1	OCRIPLASMIN	retinal vein occlusion	1	https://clinicaltrials.gov/study/NCT02747030
COL18A1	OCRIPLASMIN	stroke	2	https://clinicaltrials.gov/study/NCT00123266
COL18A1	OCRIPLASMIN	uveitis	1	https://clinicaltrials.gov/study/NCT01194674
EPHA2	DASATINIB	Alzheimer disease	1	https://clinicaltrials.gov/study/NCT04063124
EPHA2	DASATINIB	Blast Phase Chronic Myelogenous Leukemia, BCR-ABL1 Positive	4	https://www.ema.europa.eu/en/medicines/human/EPAR/dasatinib-accordpharma
EPHA2	DASATINIB	Burkitts lymphoma	1	https://clinicaltrials.gov/study/NCT00608361
EPHA2	DASATINIB	COVID-19	2	https://clinicaltrials.gov/study/NCT04830735
EPHA2	DASATINIB	Central Nervous System Neoplasm	1	https://clinicaltrials.gov/study/NCT00869401
EPHA2	DASATINIB	HIV infection	2	https://clinicaltrials.gov/study/NCT05780073
EPHA2	DASATINIB	HIV-1 infection	2	https://clinicaltrials.gov/study/NCT05527418
EPHA2	DASATINIB	Hodgkins lymphoma	1	https://clinicaltrials.gov/study/NCT01643603
EPHA2	DASATINIB	MALT lymphoma	1	https://clinicaltrials.gov/study/NCT00608361
EPHA2	DASATINIB	Mantle cell lymphoma	1	https://clinicaltrials.gov/study/NCT00608361
EPHA2	DASATINIB	Paraganglioma	2	https://clinicaltrials.gov/study/NCT03352427
EPHA2	DASATINIB	Pineoblastoma	2	https://clinicaltrials.gov/study/NCT02596828
EPHA2	DASATINIB	Sezary's disease	1	https://clinicaltrials.gov/study/NCT00608361
EPHA2	DASATINIB	Splenic Marginal Zone Lymphoma	1	https://clinicaltrials.gov/study/NCT00608361
EPHA2	DASATINIB	T-cell large granular lymphocyte leukemia	1	https://clinicaltrials.gov/study/NCT00608361
EPHA2	DASATINIB	Waldenstrom macroglobulinemia	1	https://clinicaltrials.gov/study/NCT00608361
EPHA2	DASATINIB	acute lymphoblastic leukemia	4	https://clinicaltrials.gov/study/NCT02690922
EPHA2	DASATINIB	acute myeloid leukemia	3	https://clinicaltrials.gov/study/NCT02013648
EPHA2	DASATINIB	adult B acute lymphoblastic leukemia	1	https://clinicaltrials.gov/study/NCT00608361

EPHA2	DASATINIB	adult T acute lymphoblastic leukemia	1	https://clinicaltrials.gov/study/NCT00608361
EPHA2	DASATINIB	adult T-cell leukemia/lymphoma	1	https://clinicaltrials.gov/study/NCT00608361
EPHA2	DASATINIB	adult acute lymphoblastic leukemia	1	https://clinicaltrials.gov/study/NCT02819804
EPHA2	DASATINIB	adult glioblastoma	2	https://clinicaltrials.gov/study/NCT00423735
EPHA2	DASATINIB	adult hepatocellular carcinoma	2	https://clinicaltrials.gov/study/NCT00459108
EPHA2	DASATINIB	aging	2	https://clinicaltrials.gov/study/NCT04946383
EPHA2	DASATINIB	anaplastic large cell lymphoma	1	https://clinicaltrials.gov/study/NCT00608361
EPHA2	DASATINIB	angioimmunoblastic T-cell lymphoma	1	https://clinicaltrials.gov/study/NCT00608361
EPHA2	DASATINIB	brain disease	2	https://clinicaltrials.gov/study/NCT02661113
EPHA2	DASATINIB	brain neoplasm	2	https://clinicaltrials.gov/study/NCT00423735
EPHA2	DASATINIB	breast cancer	2	https://clinicaltrials.gov/study/NCT00817531,https://clinicaltrials.gov/study/NCT00780676
EPHA2	DASATINIB	breast carcinoma	1	https://clinicaltrials.gov/study/NCT05198843
EPHA2	DASATINIB	breast neoplasm	2	https://clinicaltrials.gov/study/NCT02720185
EPHA2	DASATINIB	cancer	2	https://clinicaltrials.gov/study/NCT02750514
EPHA2	DASATINIB	central nervous system leukemia	3	https://clinicaltrials.gov/study/NCT02883049
EPHA2	DASATINIB	childhood acute lymphoblastic leukemia	1	https://clinicaltrials.gov/study/NCT02819804
EPHA2	DASATINIB	childhood cancer	2	https://clinicaltrials.gov/study/NCT04733534
EPHA2	DASATINIB	cholangiocarcinoma	2	https://clinicaltrials.gov/study/NCT02428855
EPHA2	DASATINIB	chronic kidney disease	2	https://clinicaltrials.gov/study/NCT02848131
EPHA2	DASATINIB	chronic lymphocytic leukemia	2	https://clinicaltrials.gov/study/NCT01051115
EPHA2	DASATINIB	chronic myelogenous leukemia	4	https://dailymed.nlm.nih.gov/dailymed/drugInfo.cfm?setid=0e7f054c-7a27-4192-bd1c-6115d8be858f,https://dailymed.nlm.nih.gov/dailymed/drugInfo.cfm?setid=4764f37b-c9e6-4ede-bcc2-8a03b7c521df
EPHA2	DASATINIB	colorectal cancer	1	https://clinicaltrials.gov/study/NCT00501410
EPHA2	DASATINIB	core binding factor acute myeloid leukemia	2	https://clinicaltrials.gov/study/NCT01238211
EPHA2	DASATINIB	cutaneous melanoma	2	https://clinicaltrials.gov/study/NCT00700882
EPHA2	DASATINIB	diffuse intrinsic pontine glioma	2	https://clinicaltrials.gov/study/NCT02233049
EPHA2	DASATINIB	diffuse large B-cell lymphoma	2	https://clinicaltrials.gov/study/NCT00918463
EPHA2	DASATINIB	endometrial cancer	0.5	https://clinicaltrials.gov/study/NCT01482728

EPHA2	DASATINIB	extranodal nasal NK/T cell lymphoma	1	https://clinicaltrials.gov/study/NCT00608361
EPHA2	DASATINIB	fallopian tube cancer	1	https://clinicaltrials.gov/study/NCT00672295
EPHA2	DASATINIB	follicular lymphoma	1	https://clinicaltrials.gov/study/NCT00608361
EPHA2	DASATINIB	gastrointestinal stromal tumor	2	https://clinicaltrials.gov/study/NCT00568750
EPHA2	DASATINIB	glioblastoma multiforme	2	https://clinicaltrials.gov/study/NCT02661113
EPHA2	DASATINIB	gliosarcoma	2	https://clinicaltrials.gov/study/NCT00423735
EPHA2	DASATINIB	head and neck malignant neoplasia	1	https://clinicaltrials.gov/study/NCT00882583
EPHA2	DASATINIB	head and neck squamous cell carcinoma	2	https://clinicaltrials.gov/study/NCT01488318
EPHA2	DASATINIB	hepatosplenic T-cell lymphoma	1	https://clinicaltrials.gov/study/NCT00608361
EPHA2	DASATINIB	idiopathic pulmonary fibrosis	1	https://clinicaltrials.gov/study/NCT02874989
EPHA2	DASATINIB	leukemia	3	https://clinicaltrials.gov/study/NCT03020030
EPHA2	DASATINIB	liver cancer	2	https://clinicaltrials.gov/study/NCT00459108
EPHA2	DASATINIB	lung cancer	2	https://clinicaltrials.gov/study/NCT00858403
EPHA2	DASATINIB	lymphoblastic lymphoma	2	https://clinicaltrials.gov/study/NCT02043587
EPHA2	DASATINIB	lymphoid leukemia	4	https://www.ema.europa.eu/en/medicines/human/EPAR/dasatinib-accordpharma
EPHA2	DASATINIB	lymphoma	2	https://clinicaltrials.gov/study/NCT00364286
EPHA2	DASATINIB	major salivary gland cancer	2	https://clinicaltrials.gov/study/NCT00859937
EPHA2	DASATINIB	malignant colon neoplasm	2	https://clinicaltrials.gov/study/NCT00504153
EPHA2	DASATINIB	malignant glioma	2	https://clinicaltrials.gov/study/NCT03352427
EPHA2	DASATINIB	malignant pleural mesothelioma	1	https://clinicaltrials.gov/study/NCT00652574
EPHA2	DASATINIB	melanoma	2	https://clinicaltrials.gov/study/NCT00436605
EPHA2	DASATINIB	mesothelioma	2	https://clinicaltrials.gov/study/NCT00509041
EPHA2	DASATINIB	metastatic colorectal cancer	2	https://clinicaltrials.gov/study/NCT05725200
EPHA2	DASATINIB	metastatic malignant neoplasm	2	https://clinicaltrials.gov/study/NCT00410813
EPHA2	DASATINIB	metastatic melanoma	2	https://clinicaltrials.gov/study/NCT01876212
EPHA2	DASATINIB	metastatic prostate cancer	2	https://clinicaltrials.gov/study/NCT04925648
EPHA2	DASATINIB	multiple myeloma	2	https://clinicaltrials.gov/study/NCT00429949
EPHA2	DASATINIB	mycosis fungoides	1	https://clinicaltrials.gov/study/NCT00608361
EPHA2	DASATINIB	myelodysplastic syndrome	1	https://clinicaltrials.gov/study/NCT00892190
EPHA2	DASATINIB	myeloid leukemia	1	https://clinicaltrials.gov/study/NCT01643603
EPHA2	DASATINIB	neoplasm	4	https://www.whocc.no/atc_ddd_index/?code=L01EA02

EPHA2	DASATINIB	neuroblastoma	2	https://clinicaltrials.gov/study/NCT01467986
EPHA2	DASATINIB	nodal marginal zone B-cell lymphoma	1	https://clinicaltrials.gov/study/NCT00608361
EPHA2	DASATINIB	non-Hodgkins lymphoma	1	https://clinicaltrials.gov/study/NCT01643603
EPHA2	DASATINIB	non-small cell lung carcinoma	2	https://clinicaltrials.gov/study/NCT00459342
EPHA2	DASATINIB	obesity	2	https://clinicaltrials.gov/study/NCT05653258
EPHA2	DASATINIB	ovarian cancer	1	https://clinicaltrials.gov/study/NCT00672295
EPHA2	DASATINIB	pancreatic adenocarcinoma	2	https://clinicaltrials.gov/study/NCT00474812
EPHA2	DASATINIB	pancreatic carcinoma	2	https://clinicaltrials.gov/study/NCT00474812,https://clinicaltrials.gov/study/NCT01395017,https://clinicaltrials.gov/study/NCT01652976
EPHA2	DASATINIB	peritoneum cancer	1	https://clinicaltrials.gov/study/NCT00672295
EPHA2	DASATINIB	polycythemia vera	2	https://clinicaltrials.gov/study/NCT00538980
EPHA2	DASATINIB	prostate adenocarcinoma	3	https://clinicaltrials.gov/study/NCT00744497
EPHA2	DASATINIB	prostate cancer	2	https://clinicaltrials.gov/study/NCT01990196
EPHA2	DASATINIB	rectum cancer	2	https://clinicaltrials.gov/study/NCT00504153
EPHA2	DASATINIB	refractory hairy cell leukemia	1	https://clinicaltrials.gov/study/NCT00608361
EPHA2	DASATINIB	rhabdomyosarcoma	1	https://clinicaltrials.gov/study/NCT03041701
EPHA2	DASATINIB	salivary gland adenoid cystic carcinoma	2	https://clinicaltrials.gov/study/NCT00859937
EPHA2	DASATINIB	scleroderma	1	https://clinicaltrials.gov/study/NCT00764309
EPHA2	DASATINIB	small cell lung carcinoma	2	https://clinicaltrials.gov/study/NCT00470054
EPHA2	DASATINIB	small intestine lymphoma	1	https://clinicaltrials.gov/study/NCT00608361
EPHA2	DASATINIB	squamous cell carcinoma	2	https://clinicaltrials.gov/study/NCT05724329
EPHA2	DASATINIB	squamous cell lung carcinoma	2	https://clinicaltrials.gov/study/NCT01491633
EPHA2	DASATINIB	systemic mastocytosis	2	https://clinicaltrials.gov/study/NCT00979160
EPHA2	DASATINIB	therapy related acute myeloid leukemia and myelodysplastic syndrome	2	https://clinicaltrials.gov/study/NCT01238211
EPHA2	DASATINIB	triple-negative breast cancer	2	https://clinicaltrials.gov/study/NCT06355037
EPHA2	DASATINIB	unspecified peripheral T-cell lymphoma	1	https://clinicaltrials.gov/study/NCT05559008
EPHA2	REGORAFENIB	Digestive System Carcinoma	1	https://clinicaltrials.gov/study/NCT02386397
EPHA2	REGORAFENIB	Malignant Bone Neoplasm	1	https://clinicaltrials.gov/study/NCT05830084
EPHA2	REGORAFENIB	acute myeloid leukemia	1	https://clinicaltrials.gov/study/NCT03042689
EPHA2	REGORAFENIB	adenoid cystic carcinoma	2	https://clinicaltrials.gov/study/NCT02098538

EPHA2	REGORAFENIB	angiosarcoma	2	https://clinicaltrials.gov/study/NCT02048722
EPHA2	REGORAFENIB	bile duct carcinoma	2	https://clinicaltrials.gov/study/NCT02115542
EPHA2	REGORAFENIB	biliary tract cancer	2	https://clinicaltrials.gov/study/NCT05820906
EPHA2	REGORAFENIB	cancer	2	https://clinicaltrials.gov/study/NCT02241720
EPHA2	REGORAFENIB	cholangiocarcinoma	2	https://clinicaltrials.gov/study/NCT02162914
EPHA2	REGORAFENIB	colon adenocarcinoma	2	https://clinicaltrials.gov/study/NCT02368886
EPHA2	REGORAFENIB	colorectal adenocarcinoma	2	https://clinicaltrials.gov/study/NCT01189903
EPHA2	REGORAFENIB	colorectal cancer	3	https://clinicaltrials.gov/study/NCT06199973,https://clinicaltrials.gov/study/NCT05425940
EPHA2	REGORAFENIB	colorectal carcinoma	2	https://clinicaltrials.gov/study/NCT03844620
EPHA2	REGORAFENIB	colorectal neoplasm	4	https://www.ema.europa.eu/en/medicines/human/EPAR/stivarga
EPHA2	REGORAFENIB	cutaneous melanoma	2	https://clinicaltrials.gov/study/NCT02587650
EPHA2	REGORAFENIB	digestive system cancer	2	https://clinicaltrials.gov/study/NCT06099821
EPHA2	REGORAFENIB	esophageal cancer	3	https://clinicaltrials.gov/study/NCT02773524
EPHA2	REGORAFENIB	fallopian tube cancer	2	https://clinicaltrials.gov/study/NCT02278783
EPHA2	REGORAFENIB	gastric cancer	2	https://clinicaltrials.gov/study/NCT03627728
EPHA2	REGORAFENIB	gastrointestinal stromal tumor	3	https://clinicaltrials.gov/study/NCT01271712,https://clinicaltrials.gov/study/NCT03465722
EPHA2	REGORAFENIB	glioblastoma multiforme	2	https://clinicaltrials.gov/study/NCT03970447
EPHA2	REGORAFENIB	grade III meningioma	2	https://clinicaltrials.gov/study/NCT06275919
EPHA2	REGORAFENIB	hepatocellular carcinoma	3	https://clinicaltrials.gov/study/NCT04777851
EPHA2	REGORAFENIB	intrahepatic cholangiocarcinoma	2	https://clinicaltrials.gov/study/NCT05535647
EPHA2	REGORAFENIB	liposarcoma	2	https://clinicaltrials.gov/study/NCT02048371
EPHA2	REGORAFENIB	liver neoplasm	2	https://clinicaltrials.gov/study/NCT04864054
EPHA2	REGORAFENIB	macular degeneration	2	https://clinicaltrials.gov/study/NCT02222207
EPHA2	REGORAFENIB	malignant colon neoplasm	3	https://clinicaltrials.gov/study/NCT02664077
EPHA2	REGORAFENIB	malignant renal pelvis neoplasm	2	https://clinicaltrials.gov/study/NCT02459119
EPHA2	REGORAFENIB	melanoma	2	https://clinicaltrials.gov/study/NCT02501551
EPHA2	REGORAFENIB	metastatic colorectal cancer	3	https://clinicaltrials.gov/study/NCT01103323
EPHA2	REGORAFENIB	myelodysplastic syndrome	1	https://clinicaltrials.gov/study/NCT06454409
EPHA2	REGORAFENIB	neoplasm	4	https://www.whocc.no/atc_ddd_index/?code=L01EX05
EPHA2	REGORAFENIB	osteosarcoma	2	https://clinicaltrials.gov/study/NCT04803877
EPHA2	REGORAFENIB	ovarian cancer	2	https://clinicaltrials.gov/study/NCT02278783
EPHA2	REGORAFENIB	ovarian carcinoma	2	https://clinicaltrials.gov/study/NCT02584465

EPHA2	REGORAFENIB	ovarian neoplasm	2	https://clinicaltrials.gov/study/NCT02736305
EPHA2	REGORAFENIB	pancreatic adenocarcinoma	2	https://clinicaltrials.gov/study/NCT02383433
EPHA2	REGORAFENIB	pancreatic carcinoma	2	https://clinicaltrials.gov/study/NCT02080260
EPHA2	REGORAFENIB	primary peritoneal carcinoma	2	https://clinicaltrials.gov/study/NCT02278783
EPHA2	REGORAFENIB	rectum cancer	2	https://clinicaltrials.gov/study/NCT02368886
EPHA2	REGORAFENIB	renal cell carcinoma	2	https://clinicaltrials.gov/study/NCT00664326
EPHA2	REGORAFENIB	rhabdomyosarcoma	1	https://clinicaltrials.gov/study/NCT04625907
EPHA2	REGORAFENIB	sarcoma	2	https://clinicaltrials.gov/study/NCT01900743
EPHA2	REGORAFENIB	small cell lung carcinoma	1	https://clinicaltrials.gov/study/NCT01187615
EPHA2	REGORAFENIB	soft tissue sarcoma	2	https://clinicaltrials.gov/study/NCT02048722
EPHA2	REGORAFENIB	thyroid cancer	2	https://clinicaltrials.gov/study/NCT02657551
EPHA2	VANDETANIB	Central Nervous System Neoplasm	1	https://clinicaltrials.gov/study/NCT00472017
EPHA2	VANDETANIB	Paraganglioma	1	https://clinicaltrials.gov/study/NCT01941849
EPHA2	VANDETANIB	Poorly Differentiated Thyroid Gland Carcinoma	2	https://clinicaltrials.gov/study/NCT03630120
EPHA2	VANDETANIB	acute myeloid leukemia	2	https://clinicaltrials.gov/study/NCT02638428
EPHA2	VANDETANIB	adrenal gland pheochromocytoma	1	https://clinicaltrials.gov/study/NCT01941849
EPHA2	VANDETANIB	anaplastic astrocytoma	2	https://clinicaltrials.gov/study/NCT00995007
EPHA2	VANDETANIB	anaplastic oligodendroglioma	2	https://clinicaltrials.gov/study/NCT00995007
EPHA2	VANDETANIB	bladder transitional cell carcinoma	2	https://clinicaltrials.gov/study/NCT00880334
EPHA2	VANDETANIB	breast cancer	2	https://clinicaltrials.gov/study/NCT02299999
EPHA2	VANDETANIB	breast neoplasm	2	https://clinicaltrials.gov/study/NCT00034918
EPHA2	VANDETANIB	cancer	2	https://clinicaltrials.gov/study/NCT00500292
EPHA2	VANDETANIB	carcinoma	1	https://clinicaltrials.gov/study/NCT00506051
EPHA2	VANDETANIB	clear cell renal carcinoma	2	https://clinicaltrials.gov/study/NCT01372813
EPHA2	VANDETANIB	colorectal adenocarcinoma	1	https://clinicaltrials.gov/study/NCT00507091,https://clinicaltrials.gov/study/NCT00499850
EPHA2	VANDETANIB	colorectal cancer	2	https://clinicaltrials.gov/study/NCT00454116
EPHA2	VANDETANIB	colorectal neoplasm	1	https://clinicaltrials.gov/study/NCT00532909
EPHA2	VANDETANIB	diffuse intrinsic pontine glioma	1	https://clinicaltrials.gov/study/NCT00996723
EPHA2	VANDETANIB	esophageal carcinoma	1	https://clinicaltrials.gov/study/NCT01183559
EPHA2	VANDETANIB	fallopian tube cancer	2	https://clinicaltrials.gov/study/NCT00872989
EPHA2	VANDETANIB	follicular thyroid carcinoma	2	https://clinicaltrials.gov/study/NCT03630120
EPHA2	VANDETANIB	gastric cancer	2	https://clinicaltrials.gov/study/NCT00683787

EPHA2	VANDETANIB	gastroesophageal junction adenocarcinoma	1	https://clinicaltrials.gov/study/NCT01183559
EPHA2	VANDETANIB	gastrointestinal stromal tumor	2	https://clinicaltrials.gov/study/NCT02015065
EPHA2	VANDETANIB	glioblastoma multiforme	2	https://clinicaltrials.gov/study/NCT00995007
EPHA2	VANDETANIB	gliosarcoma	2	https://clinicaltrials.gov/study/NCT00995007
EPHA2	VANDETANIB	head and neck malignant neoplasia	2	https://clinicaltrials.gov/study/NCT00720083
EPHA2	VANDETANIB	head and neck squamous cell carcinoma	2	https://clinicaltrials.gov/study/NCT00459043
EPHA2	VANDETANIB	hepatocellular carcinoma	2	https://clinicaltrials.gov/study/NCT00508001
EPHA2	VANDETANIB	kidney cancer	2	https://clinicaltrials.gov/study/NCT00566995
EPHA2	VANDETANIB	lip and oral cavity squamous cell carcinoma	2	https://clinicaltrials.gov/study/NCT01414426
EPHA2	VANDETANIB	low grade glioma	1	https://clinicaltrials.gov/study/NCT00272350
EPHA2	VANDETANIB	lung cancer	3	https://clinicaltrials.gov/study/NCT00312377
EPHA2	VANDETANIB	malignant glioma	1	https://clinicaltrials.gov/study/NCT00272350,https://clinicaltrials.gov/study/NCT00822887
EPHA2	VANDETANIB	medullary thyroid gland carcinoma	4	https://dailymed.nlm.nih.gov/dailymed/drugInfo.cfm?setid=e5721cb8-4185-47b9-bbb3-1c587e558a03
EPHA2	VANDETANIB	mesothelioma	2	https://clinicaltrials.gov/study/NCT00597116
EPHA2	VANDETANIB	metastasis	2	https://clinicaltrials.gov/study/NCT00034918
EPHA2	VANDETANIB	metastatic malignant neoplasm	1	https://clinicaltrials.gov/study/NCT01582191
EPHA2	VANDETANIB	multiple myeloma	2	https://clinicaltrials.gov/study/NCT00047788
EPHA2	VANDETANIB	neoplasm	4	https://www.whocc.no/atc_ddd_index/?code=L01EX04
EPHA2	VANDETANIB	neuroblastoma	1	https://clinicaltrials.gov/study/NCT00533169
EPHA2	VANDETANIB	non-small cell lung carcinoma	3	https://clinicaltrials.gov/study/NCT00418886,https://clinicaltrials.gov/study/NCT00404924,https://clinicaltrials.gov/study/NCT00312377,https://clinicaltrials.gov/study/NCT00364351
EPHA2	VANDETANIB	oligoastrocytoma	2	https://clinicaltrials.gov/study/NCT00995007
EPHA2	VANDETANIB	ovarian cancer	2	https://clinicaltrials.gov/study/NCT00872989
EPHA2	VANDETANIB	pancreatic carcinoma	2	https://clinicaltrials.gov/study/NCT01601808
EPHA2	VANDETANIB	papillary thyroid carcinoma	2	https://clinicaltrials.gov/study/NCT03630120
EPHA2	VANDETANIB	peritoneum cancer	2	https://clinicaltrials.gov/study/NCT00872989
EPHA2	VANDETANIB	prostate cancer	2	https://clinicaltrials.gov/study/NCT00757692,https://clinicaltrials.gov/study/NCT00659438
EPHA2	VANDETANIB	prostate carcinoma	2	https://clinicaltrials.gov/study/NCT00498797
EPHA2	VANDETANIB	rectum cancer	1	https://clinicaltrials.gov/study/NCT00532909

EPHA2	VANDETANIB	refractory malignant neoplasm	1	https://clinicaltrials.gov/study/NCT01582191
EPHA2	VANDETANIB	renal pelvis/ureter urothelial carcinoma	2	https://clinicaltrials.gov/study/NCT01191892
EPHA2	VANDETANIB	small cell lung carcinoma	2	https://clinicaltrials.gov/study/NCT00613626
EPHA2	VANDETANIB	stomach neoplasm	1	https://clinicaltrials.gov/study/NCT01183559
EPHA2	VANDETANIB	thyroid cancer	4	https://clinicaltrials.gov/study/NCT01496313
EPHA2	VANDETANIB	thyroid carcinoma	4	https://dailymed.nlm.nih.gov/dailymed/drugInfo.cfm?setid=4dc7f0af-77fb-4eec-46b9-dd1c2dcb4525
EPHA2	VANDETANIB	thyroid neoplasm	4	https://www.ema.europa.eu/en/medicines/human/EPAR/caprelsa
EPHA2	VANDETANIB	ureter cancer	2	https://clinicaltrials.gov/study/NCT01191892
EPHA2	VANDETANIB	urethra cancer	2	https://clinicaltrials.gov/study/NCT01191892
EPHA2	VANDETANIB	urinary bladder cancer	2	https://clinicaltrials.gov/study/NCT00880334,https://clinicaltrials.gov/study/NCT01191892
EPHA2	VANDETANIB	verrucous carcinoma of oral cavity	2	https://clinicaltrials.gov/study/NCT01414426
EPHB4	JI-101	malignant colon neoplasm	1	https://clinicaltrials.gov/study/NCT01149434
EPHB4	JI-101	neoplasm	1	https://clinicaltrials.gov/study/NCT00842335
EPHB4	JI-101	ovarian cancer	1	https://clinicaltrials.gov/study/NCT01149434
EPHB4	TESEVATINIB	Autosomal dominant polycystic kidney disease	2	https://clinicaltrials.gov/study/NCT02616055
EPHB4	TESEVATINIB	autosomal recessive polycystic kidney disease	1	https://clinicaltrials.gov/study/NCT03096080
EPHB4	TESEVATINIB	brain neoplasm	2	https://clinicaltrials.gov/study/NCT02844439
EPHB4	TESEVATINIB	breast cancer	1	https://clinicaltrials.gov/study/NCT02154529
EPHB4	TESEVATINIB	cancer	1	https://clinicaltrials.gov/study/NCT00086528,https://clinicaltrials.gov/study/NCT00336765
EPHB4	TESEVATINIB	glioblastoma multiforme	2	https://clinicaltrials.gov/study/NCT02844439
EPHB4	TESEVATINIB	metastatic malignant neoplasm	1	https://clinicaltrials.gov/study/NCT02154529
EPHB4	TESEVATINIB	non-small cell lung carcinoma	3	https://clinicaltrials.gov/study/NCT01487174
EPHB4	TG100-801	choroidal neovascularization	2	https://clinicaltrials.gov/study/NCT00509548
EPHB4	TG100-801	diabetic retinopathy	1	https://clinicaltrials.gov/study/NCT00414999
EPHB4	TG100-801	macular degeneration	2	https://clinicaltrials.gov/study/NCT00509548
EPHB4	VANDETANIB	Central Nervous System Neoplasm	1	https://clinicaltrials.gov/study/NCT00472017
EPHB4	VANDETANIB	Paraganglioma	1	https://clinicaltrials.gov/study/NCT01941849
EPHB4	VANDETANIB	Poorly Differentiated Thyroid Gland Carcinoma	2	https://clinicaltrials.gov/study/NCT03630120

EPHB4	VANDETANIB	acute myeloid leukemia	2	https://clinicaltrials.gov/study/NCT02638428
EPHB4	VANDETANIB	adrenal gland pheochromocytoma	1	https://clinicaltrials.gov/study/NCT01941849
EPHB4	VANDETANIB	anaplastic astrocytoma	2	https://clinicaltrials.gov/study/NCT00995007
EPHB4	VANDETANIB	anaplastic oligodendroglioma	2	https://clinicaltrials.gov/study/NCT00995007
EPHB4	VANDETANIB	bladder transitional cell carcinoma	2	https://clinicaltrials.gov/study/NCT00880334
EPHB4	VANDETANIB	breast cancer	2	https://clinicaltrials.gov/study/NCT00494481
EPHB4	VANDETANIB	breast neoplasm	2	https://clinicaltrials.gov/study/NCT00034918
EPHB4	VANDETANIB	cancer	2	https://clinicaltrials.gov/study/NCT00500292
EPHB4	VANDETANIB	carcinoma	1	https://clinicaltrials.gov/study/NCT00506051
EPHB4	VANDETANIB	clear cell renal carcinoma	2	https://clinicaltrials.gov/study/NCT01372813
EPHB4	VANDETANIB	colorectal adenocarcinoma	1	https://clinicaltrials.gov/study/NCT00499850,https://clinicaltrials.gov/study/NCT00507091
EPHB4	VANDETANIB	colorectal cancer	2	https://clinicaltrials.gov/study/NCT00454116
EPHB4	VANDETANIB	colorectal neoplasm	1	https://clinicaltrials.gov/study/NCT00532909
EPHB4	VANDETANIB	diffuse intrinsic pontine glioma	1	https://clinicaltrials.gov/study/NCT00996723
EPHB4	VANDETANIB	esophageal carcinoma	1	https://clinicaltrials.gov/study/NCT01183559
EPHB4	VANDETANIB	fallopian tube cancer	2	https://clinicaltrials.gov/study/NCT00872989
EPHB4	VANDETANIB	follicular thyroid carcinoma	2	https://clinicaltrials.gov/study/NCT03630120
EPHB4	VANDETANIB	gastric cancer	2	https://clinicaltrials.gov/study/NCT00683787
EPHB4	VANDETANIB	gastroesophageal junction adenocarcinoma	1	https://clinicaltrials.gov/study/NCT01183559
EPHB4	VANDETANIB	gastrointestinal stromal tumor	2	https://clinicaltrials.gov/study/NCT02015065
EPHB4	VANDETANIB	glioblastoma multiforme	2	https://clinicaltrials.gov/study/NCT00995007
EPHB4	VANDETANIB	gliosarcoma	2	https://clinicaltrials.gov/study/NCT00995007
EPHB4	VANDETANIB	head and neck malignant neoplasia	2	https://clinicaltrials.gov/study/NCT00720083
EPHB4	VANDETANIB	head and neck squamous cell carcinoma	2	https://clinicaltrials.gov/study/NCT00459043
EPHB4	VANDETANIB	hepatocellular carcinoma	2	https://clinicaltrials.gov/study/NCT00508001
EPHB4	VANDETANIB	kidney cancer	2	https://clinicaltrials.gov/study/NCT00566995
EPHB4	VANDETANIB	lip and oral cavity squamous cell carcinoma	2	https://clinicaltrials.gov/study/NCT01414426
EPHB4	VANDETANIB	low grade glioma	1	https://clinicaltrials.gov/study/NCT00272350
EPHB4	VANDETANIB	lung cancer	3	https://clinicaltrials.gov/study/NCT00312377
EPHB4	VANDETANIB	malignant glioma	1	https://clinicaltrials.gov/study/NCT00822887,https://clinicaltrials.gov/study/NCT00272350

EPHB4	VANDETANIB	medullary thyroid gland carcinoma	4	https://dailymed.nlm.nih.gov/dailymed/drugInfo.cfm?setid=e5721cb8-4185-47b9-bbb3-1c587e558a03
EPHB4	VANDETANIB	mesothelioma	2	https://clinicaltrials.gov/study/NCT00597116
EPHB4	VANDETANIB	metastasis	2	https://clinicaltrials.gov/study/NCT00034918
EPHB4	VANDETANIB	metastatic malignant neoplasm	1	https://clinicaltrials.gov/study/NCT01582191
EPHB4	VANDETANIB	multiple myeloma	2	https://clinicaltrials.gov/study/NCT00047788
EPHB4	VANDETANIB	neoplasm	4	https://www.whocc.no/atc_ddd_index/?code=L01EX04
EPHB4	VANDETANIB	neuroblastoma	1	https://clinicaltrials.gov/study/NCT00533169
EPHB4	VANDETANIB	non-small cell lung carcinoma	3	https://clinicaltrials.gov/study/NCT00312377,https://clinicaltrials.gov/study/NCT00404924,https://clinicaltrials.gov/study/NCT00418886,https://clinicaltrials.gov/study/NCT00364351
EPHB4	VANDETANIB	oligoastrocytoma	2	https://clinicaltrials.gov/study/NCT00995007
EPHB4	VANDETANIB	ovarian cancer	2	https://clinicaltrials.gov/study/NCT00872989
EPHB4	VANDETANIB	pancreatic carcinoma	2	https://clinicaltrials.gov/study/NCT01601808
EPHB4	VANDETANIB	papillary thyroid carcinoma	2	https://clinicaltrials.gov/study/NCT03630120
EPHB4	VANDETANIB	peritoneum cancer	2	https://clinicaltrials.gov/study/NCT00872989
EPHB4	VANDETANIB	prostate cancer	2	https://clinicaltrials.gov/study/NCT00686036
EPHB4	VANDETANIB	prostate carcinoma	2	https://clinicaltrials.gov/study/NCT00498797
EPHB4	VANDETANIB	rectum cancer	1	https://clinicaltrials.gov/study/NCT00532909
EPHB4	VANDETANIB	refractory malignant neoplasm	1	https://clinicaltrials.gov/study/NCT01582191
EPHB4	VANDETANIB	renal pelvis/ureter urothelial carcinoma	2	https://clinicaltrials.gov/study/NCT01191892
EPHB4	VANDETANIB	small cell lung carcinoma	2	https://clinicaltrials.gov/study/NCT00613626
EPHB4	VANDETANIB	stomach neoplasm	1	https://clinicaltrials.gov/study/NCT01183559
EPHB4	VANDETANIB	thyroid cancer	4	https://clinicaltrials.gov/study/NCT01496313
EPHB4	VANDETANIB	thyroid carcinoma	4	https://dailymed.nlm.nih.gov/dailymed/drugInfo.cfm?setid=4dc7f0af-77fb-4eec-46b9-dd1c2dcb4525
EPHB4	VANDETANIB	thyroid neoplasm	4	https://www.ema.europa.eu/en/medicines/human/EPAR/caprelsa
EPHB4	VANDETANIB	ureter cancer	2	https://clinicaltrials.gov/study/NCT01191892
EPHB4	VANDETANIB	urethra cancer	2	https://clinicaltrials.gov/study/NCT01191892
EPHB4	VANDETANIB	urinary bladder cancer	2	https://clinicaltrials.gov/study/NCT00880334,https://clinicaltrials.gov/study/NCT01191892
EPHB4	VANDETANIB	verrucous carcinoma of oral cavity	2	https://clinicaltrials.gov/study/NCT01414426
GFRA1	LIATERMIN	Parkinson disease	1	https://clinicaltrials.gov/study/NCT00115427,https://clinicaltrials.gov/study/NCT00111982

GPNMB	GLEMBATUMUMAB	breast cancer	1	https://clinicaltrials.gov/study/NCT00704158
GPNMB	GLEMBATUMUMAB	melanoma	1	https://clinicaltrials.gov/study/NCT00412828
GPNMB	GLEMBATUMUMAB VEDOTIN	Uveal Melanoma	2	https://clinicaltrials.gov/study/NCT02363283
GPNMB	GLEMBATUMUMAB VEDOTIN	breast cancer	2	https://clinicaltrials.gov/study/NCT01156753,https://clinicaltrials.gov/study/NCT01997333
GPNMB	GLEMBATUMUMAB VEDOTIN	melanoma	2	https://clinicaltrials.gov/study/NCT02302339
GPNMB	GLEMBATUMUMAB VEDOTIN	osteosarcoma	2	https://clinicaltrials.gov/study/NCT02487979
GPNMB	GLEMBATUMUMAB VEDOTIN	squamous cell lung carcinoma	1	https://clinicaltrials.gov/study/NCT02713828
ICAM1	ALICAFORSEN	Crohn's disease	3	https://clinicaltrials.gov/study/NCT00048113,https://clinicaltrials.gov/study/NCT00048295
ICAM1	ALICAFORSEN	inflammatory bowel disease	1	https://clinicaltrials.gov/study/NCT03473626
ICAM1	ALICAFORSEN	pouchitis	3	https://clinicaltrials.gov/study/NCT02525523
ICAM1	ALICAFORSEN	ulcerative colitis	2	https://clinicaltrials.gov/study/NCT00063830,https://clinicaltrials.gov/study/NCT00063414
ICAM1	BI-505	multiple myeloma	2	https://clinicaltrials.gov/study/NCT01838369
IL18R1	IBOCTADEKIN	cancer	1	https://clinicaltrials.gov/study/NCT00085878,https://clinicaltrials.gov/study/NCT00085904
IL18R1	IBOCTADEKIN	lymphoma	1	https://clinicaltrials.gov/study/NCT00085904
IL18R1	IBOCTADEKIN	melanoma	2	https://clinicaltrials.gov/study/NCT00107718
IL18R1	IBOCTADEKIN	non-Hodgkins lymphoma	1	https://clinicaltrials.gov/study/NCT00500058
IL1RL1	ASTEGOLIMAB	COVID-19	2	https://clinicaltrials.gov/study/NCT04386616
IL1RL1	ASTEGOLIMAB	asthma	2	https://clinicaltrials.gov/study/NCT02918019
IL1RL1	ASTEGOLIMAB	atopic eczema	2	https://clinicaltrials.gov/study/NCT03747575
IL1RL1	ASTEGOLIMAB	chronic obstructive pulmonary disease	3	https://clinicaltrials.gov/study/NCT05595642,https://clinicaltrials.gov/study/NCT05878769
LGALS3	BELAPECTIN	liver disease	1	https://clinicaltrials.gov/study/NCT04332432
LGALS3	BELAPECTIN	melanoma	1	https://clinicaltrials.gov/study/NCT02575404
LGALS3	BELAPECTIN	metastatic melanoma	1	https://clinicaltrials.gov/study/NCT02117362
LGALS3	BELAPECTIN	non-alcoholic fatty liver disease	1	https://clinicaltrials.gov/study/NCT01899859
LGALS3	BELAPECTIN	non-alcoholic steatohepatitis	2	https://clinicaltrials.gov/study/NCT02421094
LGALS3	BELAPECTIN	portal hypertension	2	https://clinicaltrials.gov/study/NCT02462967
LGALS3	BELAPECTIN	psoriasis	2	https://clinicaltrials.gov/study/NCT02407041

LGALS3	DAVANAT	bile duct carcinoma	2	https://clinicaltrials.gov/study/NCT00386516
LGALS3	DAVANAT	colorectal cancer	2	https://clinicaltrials.gov/study/NCT00388700
LGALS3	DAVANAT	gallbladder cancer	2	https://clinicaltrials.gov/study/NCT00386516
LGALS3	DAVANAT	metastatic melanoma	1	https://clinicaltrials.gov/study/NCT01723813
LGALS3	OLITIGALTIN	COVID-19	1	https://clinicaltrials.gov/study/NCT04473053
LGALS3	OLITIGALTIN	idiopathic pulmonary fibrosis	2	https://clinicaltrials.gov/study/NCT03832946
LTA4H	ACEBILUSTAT	COVID-19	2	https://clinicaltrials.gov/study/NCT04662060,https://clinicaltrials.gov/study/NCT04662086
LTA4H	ACEBILUSTAT	acne	2	https://clinicaltrials.gov/study/NCT02385760
LTA4H	ACEBILUSTAT	cystic fibrosis	2	https://clinicaltrials.gov/study/NCT02443688
LTA4H	ACEBILUSTAT	lymphedema	2	https://clinicaltrials.gov/study/NCT05203835
MFGE8	BRE-3 90Y	breast cancer	1	https://clinicaltrials.gov/study/NCT00007891
MPO	VERDIPERSTAT	Parkinson disease	2	https://clinicaltrials.gov/study/NCT01527695,https://clinicaltrials.gov/study/NCT01603069
MPO	VERDIPERSTAT	amyotrophic lateral sclerosis	2	https://clinicaltrials.gov/study/NCT04436510
MPO	VERDIPERSTAT	multiple system atrophy	3	https://clinicaltrials.gov/study/NCT03952806
MPO	VERDIPERSTAT	semantic dementia	1	https://clinicaltrials.gov/study/NCT05184569
NCAM1	BB-10901	multiple myeloma	1	https://clinicaltrials.gov/study/NCT00346255
NCAM1	BB-10901	small cell lung carcinoma	1	https://clinicaltrials.gov/study/NCT00065429
NCAM1	LORVOTUZUMAB MERTANSINE	Pleuropulmonary blastoma	2	https://clinicaltrials.gov/study/NCT02452554
NCAM1	LORVOTUZUMAB MERTANSINE	Wilms tumor	2	https://clinicaltrials.gov/study/NCT02452554
NCAM1	LORVOTUZUMAB MERTANSINE	leukemia	2	https://clinicaltrials.gov/study/NCT02420873
NCAM1	LORVOTUZUMAB MERTANSINE	malignant peripheral nerve sheath tumor	2	https://clinicaltrials.gov/study/NCT02452554
NCAM1	LORVOTUZUMAB MERTANSINE	multiple myeloma	1	https://clinicaltrials.gov/study/NCT00991562
NCAM1	LORVOTUZUMAB MERTANSINE	neuroblastoma	2	https://clinicaltrials.gov/study/NCT02452554
NCAM1	LORVOTUZUMAB MERTANSINE	rhabdomyosarcoma	2	https://clinicaltrials.gov/study/NCT02452554
NCAM1	LORVOTUZUMAB MERTANSINE	small cell lung carcinoma	1	https://clinicaltrials.gov/study/NCT01237678
NCAM1	LORVOTUZUMAB MERTANSINE	synovial sarcoma	2	https://clinicaltrials.gov/study/NCT02452554

SEMA4D	PEPINEMAB	Alzheimer disease	1	https://clinicaltrials.gov/study/NCT04381468
SEMA4D	PEPINEMAB	Huntington disease	2	https://clinicaltrials.gov/study/NCT02481674
SEMA4D	PEPINEMAB	cutaneous melanoma	1	https://clinicaltrials.gov/study/NCT03769155
SEMA4D	PEPINEMAB	head and neck squamous cell carcinoma	1	https://clinicaltrials.gov/study/NCT04815720,https://clinicaltrials.gov/study/NCT03690986
SEMA4D	PEPINEMAB	multiple sclerosis	1	https://clinicaltrials.gov/study/NCT01764737
SEMA4D	PEPINEMAB	neoplasm	1	https://clinicaltrials.gov/study/NCT01313065
SEMA4D	PEPINEMAB	non-small cell lung carcinoma	1	https://clinicaltrials.gov/study/NCT03268057
SEMA4D	PEPINEMAB	osteosarcoma	1	https://clinicaltrials.gov/study/NCT03320330
SEMA4D	PEPINEMAB	pancreatic adenocarcinoma	1	https://clinicaltrials.gov/study/NCT05102721
TNFRSF1A	GSK-1995057	respiratory system disease	1	https://clinicaltrials.gov/study/NCT01587807
TNFSF12	BIIB-023	lupus nephritis	2	https://clinicaltrials.gov/study/NCT01499355,https://clinicaltrials.gov/study/NCT01930890
TNFSF12	BIIB-023	rheumatoid arthritis	1	https://clinicaltrials.gov/study/NCT00771329
TNFSF12	RO-5458640	neoplasm	1	https://clinicaltrials.gov/study/NCT01383733
TNFSF13B	ATACICEPT	IGA glomerulonephritis	3	https://clinicaltrials.gov/study/NCT04716231
TNFSF13B	ATACICEPT	lupus nephritis	3	https://clinicaltrials.gov/study/NCT05609812
TNFSF13B	ATACICEPT	multiple sclerosis	2	https://clinicaltrials.gov/study/NCT00853762,https://clinicaltrials.gov/study/NCT00642902
TNFSF13B	ATACICEPT	optic neuritis	2	https://clinicaltrials.gov/study/NCT00624468
TNFSF13B	ATACICEPT	rheumatoid arthritis	2	https://clinicaltrials.gov/study/NCT00664521,https://clinicaltrials.gov/study/NCT00595413,https://clinicaltrials.gov/study/NCT00430495
TNFSF13B	ATACICEPT	systemic lupus erythematosus	2	https://clinicaltrials.gov/study/NCT01440231
TNFSF13B	BELIMUMAB	Sjogren syndrome	2	https://clinicaltrials.gov/study/NCT02631538,https://clinicaltrials.gov/study/NCT01160666
TNFSF13B	BELIMUMAB	autoimmune polyendocrinopathy	2	https://clinicaltrials.gov/study/NCT05020782
TNFSF13B	BELIMUMAB	autoimmune thrombocytopenic purpura	3	https://clinicaltrials.gov/study/NCT05338190
TNFSF13B	BELIMUMAB	chronic lymphocytic leukemia	2	https://clinicaltrials.gov/study/NCT05069051
TNFSF13B	BELIMUMAB	emphysema	2	https://clinicaltrials.gov/study/NCT03244059
TNFSF13B	BELIMUMAB	graft versus host disease	1	https://clinicaltrials.gov/study/NCT03207958
TNFSF13B	BELIMUMAB	immune system disease	4	https://www.whocc.no/atc_ddd_index/?code=L04AA26
TNFSF13B	BELIMUMAB	kidney disease	2	https://clinicaltrials.gov/study/NCT03949855
TNFSF13B	BELIMUMAB	lupus nephritis	4	https://dailymed.nlm.nih.gov/dailymed/drugInfo.cfm?setid=2fa3c528-1777-4628-8a55-a69dae2381a3

TNFSF13B	BELIMUMAB	lymphopenia	1	https://clinicaltrials.gov/study/NCT04097561
TNFSF13B	BELIMUMAB	membranous glomerulonephritis	2	https://clinicaltrials.gov/study/NCT01762852
TNFSF13B	BELIMUMAB	multiple sclerosis	2	https://clinicaltrials.gov/study/NCT04767698
TNFSF13B	BELIMUMAB	myositis	2	https://clinicaltrials.gov/study/NCT02347891
TNFSF13B	BELIMUMAB	nephrotic syndrome	2	https://clinicaltrials.gov/study/NCT03949855
TNFSF13B	BELIMUMAB	neuromyelitis optica	1	https://clinicaltrials.gov/study/NCT05154734
TNFSF13B	BELIMUMAB	rheumatoid arthritis	2	https://clinicaltrials.gov/study/NCT00071812
TNFSF13B	BELIMUMAB	systemic lupus erythematosus	4	https://clinicaltrials.gov/study/NCT02270970
TNFSF13B	BELIMUMAB	systemic scleroderma	2	https://clinicaltrials.gov/study/NCT01670565
TNFSF13B	BELIMUMAB	vasculitis	3	https://clinicaltrials.gov/study/NCT01663623
TNFSF13B	BLISIBIMOD	Granulomatosis with Polyangiitis	2	https://clinicaltrials.gov/study/NCT01598857
TNFSF13B	BLISIBIMOD	IGA glomerulonephritis	3	https://clinicaltrials.gov/study/NCT02052219
TNFSF13B	BLISIBIMOD	autoimmune thrombocytopenic purpura	2	https://clinicaltrials.gov/study/NCT01609452
TNFSF13B	BLISIBIMOD	microscopic polyangiitis	2	https://clinicaltrials.gov/study/NCT01598857
TNFSF13B	BLISIBIMOD	systemic lupus erythematosus	3	https://clinicaltrials.gov/study/NCT01395745
TNFSF13B	TABALUMAB	chronic renal failure syndrome	2	https://clinicaltrials.gov/study/NCT01200290
TNFSF13B	TABALUMAB	multiple myeloma	2	https://clinicaltrials.gov/study/NCT01602224
TNFSF13B	TABALUMAB	relapsing-remitting multiple sclerosis	2	https://clinicaltrials.gov/study/NCT00882999
TNFSF13B	TABALUMAB	rheumatoid arthritis	3	https://clinicaltrials.gov/study/NCT01202760
TNFSF13B	TABALUMAB	systemic lupus erythematosus	3	https://clinicaltrials.gov/study/NCT02041091,https://clinicaltrials.gov/study/NCT01488708
TNFSF13B	TIBULIZUMAB	Sjogren syndrome	2	https://clinicaltrials.gov/study/NCT04563195
TNFSF13B	TIBULIZUMAB	rheumatoid arthritis	1	https://clinicaltrials.gov/study/NCT01925157

Supplementary Table 9. Comparison of AUC for each single-trait PRS and the $PRS_{FEV1+FEV1/FVC}$ from Moll et al. with the PRS_{multi}. Multivariable models were trained for either COPD or frequent exacerbations (≥ 2 per year) and compared with DeLong p-values. All models were adjusted for clinical factors (age, sex, smoking) and PCs of genetic ancestry.

**Model**	**AUC (COPD)**	**DeLong pval vs. PRSmulti (COPD)**	**AUC (Frequent Exacerbation)**	**DeLong pval vs. PRSmulti (Frequent Exacerbation)**
PRS multi	0.8	ref	0.612	ref
PRS FEV1/FVC	0.79	1.35E-06	0.609	0.00138
PRS FEV1	0.782	3.02E-12	0.607	2.75E-05
PRS DLspiro	0.779	5.84E-14	0.607	0.000361
PRS smoking	0.759	2.08E-24	0.606	0.000969
PRS IPF	0.76	9.00E-24	0.603	3.91E-06
PRS BMI	0.761	1.01E-21	0.607	0.0105
PRS CRP	0.759	6.07E-24	0.604	5.31E-05
PRS FEV1 + FEV1/FVC	0.796	0.000463	0.61	0.000166

Supplementary Table 10. Associations of PRSs in PRSmix+ and composite FEV1 and FEV1/FVC PRS with additional outcomes in COPDGene non-Hispanic White. Cox regression for all mortality and COPD related mortality and negative binomial regression for severe exacerbation and antibiotics or steroid use. Blue shading indicates the highest performing PRS in each outcome. Models were adjusted for age, sex, current smoking status, pack-years of smoking, prior exacerbations, and principal components of genetic ancestry.

PRS	All mortality				Respiratory-specific mortality				Severe exacerbation			Antibiotics or steroids		
	N samples	*N events*	*HR (95% CI)*	*adj. p-value*	*N samples*	*N events*	*HR (95% CI)*	*adj. p-value*	*N samples*	*beta (95% CI)*	*adj. p-value*	*N samples*	*beta (95% CI)*	*adj. p-value*
PRS FEV1/FVC	6096	982	1.088 (1.021, 1.159)	0.0213	6006	369	1.234 (1.114, 1.368)	0.000173	6129	0.205 (0.125, 0.286)	2.01E-08	6129	0.185 (0.136, 0.235)	1.35E-15
PRS FEV1	6096	982	1.057 (0.994, 1.125)	0.0987	6006	369	1.057 (0.956, 1.168)	0.281	6129	0.223 (0.146, 0.301)	1.21E-09	6129	0.136 (0.087, 0.185)	4.49E-09
PRS DLspiro	6096	982	1.062 (0.996, 1.133)	0.0987	6006	369	1.246 (1.121, 1.386)	0.000173	6129	0.189 (0.110, 0.268)	2.55E-07	6129	0.138 (0.089, 0.186)	3.93E-09
PRS smoking	6096	982	1.041 (0.976, 1.111)	0.252	6006	369	1.069 (0.963, 1.186)	0.273	6129	0.055 (-0.028, 0.138)	0.15	6129	0.079 (0.027, 0.131)	0.00115
PRS IPF	6096	982	0.968 (0.909, 1.031)	0.307	6006	369	0.944 (0.852, 1.045)	0.281	6129	-0.056 (-0.135, 0.022)	0.134	6129	0.010 (-0.039, 0.059)	0.672
PRS BMI	6096	982	1.157 (1.083, 1.236)	0.000143	6006	369	1.213 (1.089, 1.350)	0.001	6129	0.252 (0.168, 0.335)	2.96E-10	6129	0.146 (0.094, 0.198)	5.17E-09
PRS CRP	6096	982	1.073 (1.008, 1.143)	0.0492	6006	369	1.117 (1.009, 1.238)	0.0496	6129	0.088 (0.009, 0.167)	0.018	6129	0.102 (0.053, 0.150)	9.63E-06
PRS multi	6096	982	1.106 (1.038, 1.179)	0.00805	6006	369	1.238 (1.117, 1.373)	0.000173	6129	0.273 (0.194, 0.351)	3.26E-13	6129	0.215 (0.166, 0.264)	3.77E-20
PRS FEV1 + FEV1/FVC	6096	982	1.087 (1.021, 1.157)	0.0213	6006	369	1.177 (1.064, 1.303)	0.0028	6129	0.256 (0.176, 0.335)	5.29E-12	6129	0.192 (0.143, 0.241)	1.71E-16

Supplementary Table 11. Number of significant proteins (FDR < 0.1) at each step of the filtering process.

Trait	n significant in metaxcan	n significant in COPDGene	n significant in UKB	n significant in both	n concordant
FEV1/FVC	224	84	60	30	26
FEV1	214	90	65	34	30
DL spirometry	59	26	17	11	10
Smoking	7	3	2	1	1
IPF	1	0	0	0	0
BMI	304	114	87	33	31
CRP	155	60	49	27	26
